# Supplementary material for: Safety evaluation of the single-dose Ad26.COV2.S vaccine among healthcare workers in the Sisonke study in South Africa: A phase 3b implementation trial
Source: PLoS Med. 2022 Jun 21;19(6):e1004024. doi: 10.1371/journal.pmed.1004024 (PMC9212139; doi:10.1371/journal.pmed.1004024)
Supplement: S1 Appendix — (PDF) [file pmed.1004024.s001.pdf]

PROTOCOL

Sisonke (Together): OPEN LABEL TRIAL

Open-label, single-arm phase 3B implementation study to monitor the effectiveness of the single-dose Ad26.COV2.S COVID-19 vaccine among health care workers in South Africa (VAC31518COV3012)

CLINICAL TRIAL SPONSORED BY

South African Medical Research Council (SAMRC)

INVESTIGATIONAL PRODUCTS PROVIDED BY JANSSEN VACCINES & PREVENTION B.V.

Janssen Vaccines & Prevention B.V. is a Janssen pharmaceutical company of Johnson & Johnson (Company)

29 April 2021

Version 4.4

I, Glenda Gray have read the Department of Health: *Ethics in health research: principles, processes and structures, second edition*, 2015, the *Guidelines for Good Practice in the Conduct of Clinical Trials with Human Participants in South Africa,* Second Edition, 2006, Department of Health, Pretoria, South Africa (where applicable), and the Declaration of Helsinki (2013) and have prepared this proposal with due cognisance of its content. Furthermore, I will adhere to the principles expressed when conducting this proposed research project.

____
XXXXX
29 April 2021

PROTOCOL TEAM

**Glenda Gray MbChB, FC(Paeds) PI**<br>South African Medical Research Council	**Linda-Gail Bekker, MBChB, DTMH, DCH, FCP(SA), PhD.**<br>**Co-PI**<br>The Desmond Tutu HIV Centre<br>University of Cape Town
**Ameena Goga, MbChB, FC(Paeds), MSc, PhD**<br>**Co-PI**<br>SAMRC	**Nigel Garrett, MBBS, MRCP, MSc, PhD**<br>**Co-PI**<br>Centre for the AIDS Programme Of Research in South Africa (CAPRISA), Nelson R Mandela School of Medicine, UKZN
**Nonhlanhla Yende-Zuma, MSc, PhD Protocol Statistician**<br>CAPRISA, Nelson R Mandela School of Medicine, UKZN	**Tarylee Reddy, MSc, PhD**<br>**Statistician**<br>Biostatistics Research Unit, SAMRC
**Simba Takuva, MBChB, MSc, DTM&H**<br>**Safety Physician and Medical Monitor**<br>Hutchinson Center Research Institute of South Africa (HCRISA)	**Azwi Takalani, MBChB, MMed, FCPHM**<br>**Safety Physician**<br>Hutchinson Center Research Institute of South Africa (HCRISA)
**Fatima Mayat, B.Pharm Implementation & Oversight Support**<br>Perinatal HIV Research Unit (PHRU)	**Jackline Odhiambo, B. Pharm, PGDRM, MSc**<br>**Implementation & Oversight Support**<br>Hutchinson Center Research<br>Institute of South Africa<br>(HCRISA)
**Koleka Mlisana, MBChB, MMed Micro, PhD**<br>Academic Affairs, Research & Quality Assurance, National Health Laboratory Services,	**Ian Sanne, MBBCh, DTM&H, FCP(SA), Cert (ID)**<br>**Investigator**<br>Right to Care/Wits Health Consortium<br>(Clinical HIV Research Unit)

**Lara Fairall, MBChB, MD**<br>Collaborator<br>The Knowledge Translation Unit<br>UCT Lung Institute<br>University of Cape Town	**Lawrence Corey, MD**<br>**Advisor**<br>Fred Hutchinson Cancer Research Center<br>Seattle, Washington, USA
**Dan Barouch, MD, PhD.**<br>Harvard Medical School<br>Ragon Institute of MGH, MIT, and Harvard<br>Director, Center for Virology and Vaccine Research<br>Beth Israel Deaconess Medical Center	**Barry Jacobson, MB ChB, MMed, PhD, FRCS(Glas)**<br>**Member PSRT and advisor**<br>Department of Haematology, NHLS, Johannesburg Hospital and Head of Surgical Research, University of the Witwatersrand,
**Jonny Peter, MB ChB, MMed, FCP (SA), PhD**<br>**Member of PSRT and advisor**<br>Division of Allergology and Clinical Immunology at Groote Schuur Hospital, University of Cape Town.	Vernon Louw, MBChB (Stell), MMed(Int Med)(Stell), PhD(HPE)(UFS)<br>**Member of PSRT:**<br>Division of Clinical Medicine Department of Medicine, University of Cape Town
**Jessica Opie, MBCHB Hons (UCT) MRCP (UK) FRCPA (Haem Aus)**<br>**Member of PSRT:**<br>Division of Haematology, Department of Pathology, University of Cape Town	

COLLABORATING ORGANIZATIONS

**Collaborating partners**	**Collaborating Organisation and Investigational products provided by: Janssen Vaccines & Prevention B.V.**
National Department of Health of South Africa<br>Dr Lesley Bamford<br>National Department of Health<br><br>National Institute of Communicable Diseases:<br>Professor Penny Moore:<br>University of the Witwatersrand<br>National Institute for Communicable Diseases, a Division of the NHLS Centre for HIV & STIs<br><br>KwaZulu-Natal Research Innovation and Sequencing Platform (KRISP), Tulio de Oliveira, PhD<br>Sub-Investigator<br>KwaZulu-Natal Research Innovation and Sequencing Platform (KRISP), UKZN, Durban, South Africa<br><br>Harry Moultrie, MBBCh, MMedSc<br>University of the Witwatersrand<br>Johannesburg<br><br>Fred Hutchinson Cancer Research Center<br>Seattle, WA 98109-1024<br><br>Hutchinson Center Research Institute of South Africa (HCRISA)<br>Chris Hani Baragwanath Academic Hospital	**Supporting partners**<br>BARC<br>Johannesburg<br><br>Dis-Chem Pharmacy<br>Midrand<br>South Africa<br><br>Biovac<br>Dr Morena Makhoana CEO<br>South Africa<br><br>Biocair South Africa (Pty) Ltd<br>Leonard N. Lazarus | Africa Regional Director<br><br>Right to Care<br>Centurion, 0046<br><br>Clinical Laboratory Services<br>Corner de Korte and Hospital Streets.

**Funding for the trial is provided by:**<br>National Department of Health<br>South African Medical Research Council (SAMRC)<br>Bill & Melinda Gates Foundation<br>National Department of Science and Innovation<br>ELMA Foundation<br>Dell Foundation	
**STUDY MONITOR**	
Hutchinson Center Research Institute of South Africa<br>Chris Hani Baragwanath Academic Hospital<br>Soweto, 2196 South Africa	
**ETHICS / REGULATORY OVERSIGHT**	
**UKZN Biomedical Research Ethics Committee**	**South African Health Products Regulatory Authority (SAHPRA)**
**UCT Health Science Faculty Research Committee**<br><br>**Sefako Makgatho University Research Ethics Committee (SMUREC)**	**SAMRC Human Research Ethics Committee**<br><br>**University of Stellenbosch Health Research Ethics**

**Pharma-Ethics (Pty) Ltd**	**University of Witwatersrand Human Research Ethics Committee (Medical).**

Study sites and site PIs

City	PI Name	Site Name
Port Elizabeth	**Malan, Daniel**	PHOENIX Pharma (Pty) Ltd
Cape Town, In Groote Schuur	**Kassim, Sheetal**	Desmond Tutu Health Foundation Clinical Trials Unit
Durban	**Naiker, Nivashnee**	CAPRISA eThekwini Clinic
Cape Town (Bellville)	**Diacon, Andreas**	TASK Central
Soweto(Chris Hani Baragwanath Hospital)	**Lazarus, Erica**	Perinatal HIV Research Unit (PHRU)
Worcester	**Barnabas, Shaun**	FAM-CRU (Family Clinical Research Unit)
Mamelodi East	**Kotze, Sheena**	Synexus SA - Stanza Clinical Research Centre
Soweto (Kliptown)	**Lazarus, Erica**	Perinatal HIV Research Unit Kliptown CRS
Pretoria	**Malahleha, Mookho**	Setshaba Research Centre
Durban (situated on the grounds of RK Khan hospital)	**Naidoo, Logashvari**	SA Medical Research Council - Chatsworth
Cape Town, Crossroads/Nyang a	**Bekker, Linda-Gail, Pippa MacDonald**	Desmond Tutu HIV Foundation - **Emavundleni Research Centre**
Johannesburg (Helen Joseph)	**Badal-Faesen, Sharlaa**	Themba Lethu HIV Research Unit **(CHRU) Clinical HIV Research Unit (CHRU)**
Rustenburg	**Brumskine, William**	The Aurum Institute Rustenburg Clinical Research Centre

Hillbrow, JHB	**Patel, Faeezah**	Wits RHI - Shandukani Research
Cape Town, Guinea fowl Road Fishhoek	**Gill, Katherine**	Desmond Tutu HIV Foundation Desmond Tutu Health Foundation (DTHF) **Masiphumelele Clinic**
Klerksdorp	**Innes, Craig**	The Aurum Institute Klerksdorp Clinical Research Centre
Ladysmith	**Kotze, Philippus**	Qhakaza Mbokodo Research Clinic (QM)
Bloemfontein	**Lombaard, Johannes**	Josha Research
Khayelitsha	**Ward, Amy**	Ekhayavac TB Vaccine Trial Unit / Khayelitsha CRS (CIDRI UCT)
Tembisa	**Mngadi, Kathryn**	The Aurum Institute Tembisa Clinical Research Centre
Worcester	**Luabeya, Angelique**	SATVI, Brewelskloof Hospital
Botha's Hill, Kwa-Zulu Natal	**Spooner, Elizabeth**	SA Medical Research Council - Botha's Hill
Tongaat, KwaZulu Natal	**Naicker, Vimla**	SA Medical Research Council - Tongaat
Pretoria, Medunsa	**Nchabeleng, Maphoshan e**	Mecru Clinical Research Unit
Germiston	**Mamba, Musawenko si**	CRISMO Bertha Gxowa Research Centre
Mthatha	**Dubula, Thozama**	Nelson Mandela Academic Clinical Research Unit (NeMACRU)
KwaZulu Natal	**Makhaza, Disebo**	CAPRISA Vulindlela Clinic
Somerset West	**Urbach, Dorothea**	Synexus Helderberg Clinical Research Centre
Pretoria	**van Nieuwenhui zen, Elane**	Synexus SA Watermeyer Clinical Research Centre
Middelburg	**Petrick, Friedrich**	Mzansi Ethical Research Centre
Dennilton, Limpopo	**Maboa, Rebone Molobane**	Ndlovu Research Centre
Somerset West	**Engelbrecht, Johannes**	Dr JM Engelbrecht Clinical Trial Site
Cape Town	**Dawson, Rodney**	University of Cape Town Lung Institute
Benoni	**Hussen, Nazreen**	Worthwhile Clinical Trials
Kempton Park	**Joseph, Natasha**	Peermed CTC (Pty) Ltd T/A MERC

Pretoria	**Grobbelaar, Coert**	The Aurum Institute Clinical Research Centre Pretoria
Paarl	**Hellstrom, Lize**	Be Part -Yoluntu Centre
Parow	**Burgess, Lesley**	Tread Research

TABLE OF CONTENTS

PROTOCOL TEAM **3**

COLLABORATING ORGANIZATIONS **5**

TABLE OF CONTENTS 10

1.0 STUDY SCHEMA **12**

2.0 INTRODUCTION **14**

2.1 Study Background 14
2.2 Study design rationale 15
2.3 Nonclinical Pharmacology 15
2.4 Nonclinical Safety 15
2.5 Clinical Studies 16
2.6 Clinical safety in post-licensure roll out 19
2.7 Clinical safety on Sisonke 3B study to date (12 April 2021) 23
2.8 Clinical Safety Experience with Ad26 for non-COVID-19 vaccines 23

3.0 Objectives & Endpoints **28**

4.0 STUDY DESIGN **29**

4.1 Enrolment plan 30
4.2 Study Population 30
4.3 Eligibility criteria 30

5.0 CLINICAL PROCEDURES **31**

5.1 Recruitment 31
5.2 Screening evaluation 32
5.3 Enrolment Visits 32
5.4 Administration of study product/s 33
5.5 Follow up Procedures 33
5.5.1 Surveillance for hospitalization and breakthrough infections 33
5.5.2 Sub-cohort (volunteer 10 000 HCWs) follow up visits **33**
5.5.3 Clinical research site oversight in Sisonke 35

6.0 LABORATORY PROCEDURES **38**

The following tests will be performed in accordance with the schedule of activities: **38**

7.0 STUDY PRODUCT **39**

7.1 Preparation, Handling and Storage 39
7.2 Study product accountability 39

8.0 CLINICAL SAFETY **40**

9.0 STATISTICAL CONSIDERATIONS **46**

9.1 Study Design 46
9.2 Sample Size 46
9.3 Statistical Analyses 47

10.0 DATA MANAGEMENT **47**

10.1 Data Analyses 48
10.2 Data Sharing 48

11.0 HUMAN SUBJECT PROTECTION AND ETHICAL OBLIGATIONS **488**

11.1 Regulatory and Ethical Approval 4848
11.2 Informed consent 488
11.3 Risks and Benefits 499
11.4 Monitoring 49

References ... **50**

Appendices:

1.0 Visit Schedule For Sisonke Open Cohort ... 51
2.0 Visit Schedule for Sub-cohort ... 51
3.0 Schedule of evaluations: Participants With (Suspected) COVID-19 ... 51
4.0 Screening Form ... 52
5.0 Informed Consent Form (Elecronic, Online) ... 53
6.0 Patient Information Leaflet (English) ... 58
7.0 Printed (In Person) Informed Consent Form Template ... 66
8.0 Follow Up Visit CRF ... 83
9.0 Vaccination Visit Enrollment Visit CRF ... 84

1.0 STUDY SCHEMA

**Purpose**	To monitor the effectiveness of the single dose Ad26.COV2.S COVID-19 vaccine among health care workers (HCW) as compared to the general unvaccinated population in South Africa
**Study design**	Open-label, single-arm phase 3B vaccine implementation study
**Rationale**	South Africa is severely affected by the global COVID-19 epidemic, but currently no vaccine has been rolled out. The recent promising results of the 'ENSEMBLE' trial conducted by Janssen in South Africa, and the availability of a limited amount of research lot vaccine doses, provide the rationale for a cohort study of vaccinated HCWs to inform the larger vaccine rollout.
**Study participants**	Health Care workers age 18 and over working in the South African public and private health care sector (N=500 000)
**Study sites**	Department of Health Vaccine Administration Sites across South Africa supported by the Sisonke (Together) (VAC31518COV3012) Trial Research Site Investigators and Study Staff
**Study duration**	Participants will receive a single dose of vaccine at enrolment; to monitor outcomes the DATCOV surveillance system and NICD line lists will be reviewed for up to 2 years post-vaccination.<br>A sub-cohort (approx. 1000 -1400 people) consisting of special sub-populations will be followed up at approx. 1, 3, 6 weeks and 6 months post vaccination. This group will also have outcomes monitored for up to a further 18 months, bringing their total follow-up duration to 2 years post-vaccination.
**Study products**	Ad26.COV2.S by Janssen administered as a single injection
**Primary objectives**	• To assess the effectiveness of Ad26.COV2.S vaccine on severe COVID, hospitalizations and deaths in HCWs as compared with the general unvaccinated population in South Africa.
**Secondary objectives**	• To estimate the incidence of symptomatic SARS CoV-2 infections in HCW<br>• To estimate vaccine uptake among HCWs in South Africa<br>• To monitor the genetic diversity of breakthrough SARS CoV-2 infections<br>• To monitor immunological responses (neutralising, non-neutralising antibodies and T cell responses) in HCWs with breakthrough infections<br>• To measure baseline SARS CoV-2 antibody testing to evaluate pre-existing immunity in up to 100 000 HCWs.<br>• In a subgroup of participants (approx. 1000 -1400 people) including representative sub-populations of interest, e.g. elderly, immune compromised:

	- To compare serum neutralization and immune responses before and after vaccination at 0, 6 weeks and up to 6 months. - To explore clotting parameters post vaccination at weeks 0, 1, 3. - To monitor for asymptomatic infection
**Exploratory objectives**	• To set up a comprehensive safety desk and establish a link between the national pharmacovigilance system to assist with monitoring safety and any unexpected adverse effects

2.0 INTRODUCTION

2.1 Study Background

The global COVID-19 pandemic has had a devastating effect on South Africa. As of 04 February 2021, there have been more than 1.4 million recorded cases and 45,344 deaths. In addition, dramatic increases in hospitalizations and pressure on the health care system during the first and second waves, has led to excess deaths estimated to be at least twice as high as those reported. The second wave was fuelled by a variant virus, which has increased transmissibility by about 50%.

Nevertheless, South African researchers and policy makers have led the way in contributing to the international COVID-19 response by conducting several vaccine trials and informing global understanding of the importance of new viral variants. These include the B.1.351 lineage that was first identified in South Africa and is now circulating inside and outside the country. The B.1.351 lineage, also known as 501Y.V2 variant and 20H/501Y.V2, is a variant of SARS-CoV-2. This variant is now appearing in almost all regions of the world where genetic surveillance of SARS-CoV-2 is being undertaken. One serious concern with this and other new variants is that they may have less sensitivity to the vaccines currently in production. *In vitro* testing has shown reductions in antibody titre of up to 4-6 fold for certain vaccines.

Recently, an international randomized, double-blind, placebo-controlled phase 3 study (ENSEMBLE trial) that assessed the efficacy and safety of the Ad26.COV2.S COVID-19 (JnJ) vaccine in adults was conducted across four continents. Ad26.COV2.S is a monovalent vaccine composed of a recombinant, replication-incompetent adenovirus type 26 (Ad26) vector, constructed to encode the severe acute respiratory syndrome coronavirus-2 (SARS-CoV-2) spike (S) protein. South African trial sites contributed almost 7,000 participants to this trial of approximately 44,000 enrolled participants globally. The study is being conducted under the sponsorship of Janssen (Janssen Vaccines & Prevention B.V) in collaboration with Operation Warp Speed (OWS), which also encompasses the Biomedical Advanced Research and Development Authority (BARDA), the National Institutes of Health (NIH), and the COVID-19 Prevention Trials Network (COVPN) in the US.

The single-dose vaccine candidate demonstrated 66% effectiveness overall in preventing moderate and severe COVID-19 disease, 28 days post-vaccination (72% in the USA; 64% in South Africa). It was 85% effective overall in preventing severe disease, and there were no COVID-19 related hospitalizations and deaths, including in South Africa. Importantly, there was a high level of protection observed against severe disease cause by the SARS-CoV-2 variant from the B.1.351 variant lineage observed in South Africa (89% as of 28 days). These results are important in that they demonstrate vaccine efficacy *in vivo* against the new variant strains. There were no safety issues in this trial and similar vaccines using the same platform have been used in >200 000 people to date. Updates on safety are included in this amended protocol V4.1.

This single-dose vaccine candidate is estimated to remain stable for two years at -20°C, at least three months of which can be at temperatures of 2-8°C. This permits seamless distribution using existing vaccine supply chain channels in South Africa without the need for transportation and storage in highly specialized freezers. This is an important logistical consideration for rapid vaccine roll out.

The South African Government Covid-19 Vaccination Strategy aims to immunize 40 million individuals by the end of 2021, starting with Phase 1 with 1.5 million vaccine doses for frontline health care workers in February and March 2021. Phases 2 and 3 of the vaccination plan will commence as soon as more vaccines become available. Such a large-scale vaccination programme, delivered in a very short time frame with a substantial financial investment, is

unparalleled. Given these extraordinary investments and the need to deliver and administer vaccines safely, efficiently, and as broadly as possible, it is critical that the Phase 1 of the COVID-19 vaccine roll-out is monitored and well understood.

In this open-label, single-arm phase 3B vaccine implementation study, the overall goal will be to monitor the effectiveness of the single dose Ad26.COV2.S COVID-19 vaccine on severe COVID, hospitalizations and deaths among health care workers (HCW) in South Africa. To achieve this, the following will be measured: (1) the occurrence of hospitalizations and deaths (2) the incidence of severe SARS CoV-2 infections, (3) the diversity of breakthrough infections and (4) evaluate vaccine uptake among HCWs in South Africa. The incidence of asymptomatic infection, and immune responses to vaccination will be additionally assessed in a subset of HCW.

Stakeholder Engagement
Recent news and social media activity regarding vaccination and COVID vaccination in general suggest that a great deal of factual, effective and robust engagement in all sectors of society globally are urgently required. Although South Africa has had a good profile for vaccination willingness but we have seen an increase in vaccine hesitancy and even denialism (the so called antivax ideology) in recent months**.** This has also created some issues of trust and confidence in the study. For **these reasons** we believe there needs to be public engagement that includes:

- Inclusion the President and Deputy president of the Republic of South Africa.
- Robust communication of good information- a web site has been created for resources
- A series of webinars are being run in each province
- Unions are also being engaged and will contribute to information dissemination
- Provincial teams made up of researchers, public and private sector are contributing to information dissemination from numerous forums.

2.2 Rationale
HCW provide essential services, particularly with regard to the COVID-19 pandemic. As frontline workers they risk daily exposure to SARS-CoV-2. Despite the extensive use of non-pharmaceutical interventions, such as personal protective equipment (PPE), HCW continue to contract SARS-CoV-2, with a number of HCW developing severe disease resulting in hospitalisation or death. Even HCW who remain asymptomatic or only develop mild disease are forced to isolate – this has exacerbated staff shortages and undermined the ability of the health sector to respond to the high demand for hospital based care due to the ongoing pandemic. The South African Government Covid-19 Vaccination Strategy has already prioritised the vaccination of HCW in phase 1 of the vaccine rollout, underscoring the national agreement that HCW constitute a priority group. The proposed study will be conducted in collaboration with the National Department of Health. Implementation lessons learnt with vaccine rollout to HCW will be used to inform vaccine rollout to the public in subsequent phases.

2.3 Nonclinical Pharmacology
Nonclinical studies were performed to test the immunogenicity of different vaccine candidates, leading to the selection of the current vaccine for this development program. In addition, vaccine efficacy (VE) of Ad26.COV2-S has been shown in Syrian hamsters and NHP. Details are provided in the Investigational Brochure (1, 2).

2.4 Nonclinical Safety

Biodistribution
To assess distribution, persistence, and clearance of the Ad26 viral vector platform, intramuscular (IM) biodistribution studies have been conducted in rabbits using an Ad26-based HIV vaccine, Ad26.ENVA.01, and an Ad26-based RSV vaccine, Ad26.RSV.preF. In the available biodistribution studies, the Ad26 vector did not widely distribute following IM administration in

rabbits. Ad26 vector deoxyribonucleic acid (DNA) was primarily detected at the site of injection, draining lymph nodes and (to a lesser extent) the spleen. Clearance of the Ad26 vector from the tissues was observed. Both Ad26 vectors showed a comparable biodistribution despite carrying different antigen transgenes. These data further indicate that the Ad26 vector does not replicate and/or persist in the tissues following IM injection. These platform data are considered sufficient to inform on the biodistribution profile of Ad26.COV2.S for which the same Ad26 vector backbone is used.

Toxicology
The Company has significant nonclinical experience with Ad26-vectored vaccines using various transgenes encoding HIV, RSV, Ebola virus, filovirus, human papilloma virus, Zika, influenza (universal flu [Uniflu]), and malaria antigens. To date, more than 10 Good Laboratory Practice (GLP) combined repeated dose toxicology and local tolerance studies have been performed in rabbits (and 1 study in rats), testing the nonclinical safety of various homologous and heterologous regimens with Ad26-based vaccines at full human doses up to 1.2×1011 vp. No adverse effects have been observed in these studies. The vaccine-related effects observed were similar across studies, considered to be reflective of a physiological response to the vaccines administered, and seem to be independent of the antigen transgene. Overall, there were no safety signals detected in any of the available GLP toxicology studies with Ad26-based vaccines up to the highest dose tested (1.2×1011 vp). In a combined embryo-foetal and pre- and postnatal development GLP study in female rabbits with another Ad26-based vaccine (Ad26.ZEBOV, encoding an Ebola virus antigen), there was no maternal or developmental toxicity observed following maternal exposure during the premating and gestation period. A repeated dose and local tolerance GLP study, and a combined embryo-foetal and pre- and postnatal development GLP study with Ad26.COV2.S are planned to run in parallel with study VAC31518COV1001.

2.5 Clinical Studies

<u>*FIH VAC31518COV1001(Phase1-2a)*</u>
As of September 2020, a single injection of Ad26.COV2.S had been administered to 805 adult participants, aged 18 and older in a phase 1-2a study at centres in Belgium and USA .

The FIH VAC31518COV1001 study is a phase 1-2a trial of healthy adults between the ages of 18 and 55 years (cohort 1) and those 65 years of age or older (cohort 3) to receive the Ad26.COV2.S vaccine at a dose of 5×10^{10} viral particles (low dose) or 1×10^{11} viral particles (high dose) per ml or placebo in a single-dose or two-dose schedule. Cohort 2 collected longer-term data comparing the single -dose regimen with the two-dose regimen (3). The primary end points were the safety and reactogenicity of each dose schedule.

Table 1: VAC31518COV1001 study design

**Cohort 1a (Adults ≥18 to ≤55 years)**			
**Group**	**N**	**Day 1 (Vaccination 1)**	**Day 57 (Vaccination 2)**
1	75	Ad26.COV2.S 5×10^{10} vp	Ad26.COV2.S 5×10^{10} vp
2	75	Ad26.COV2.S 5×10^{10} vp	Placebo
3	75	Ad26.COV2.S 1×10^{11} vp	Ad26.COV2.S 1×10^{11} vp
4	75	Ad26.COV2.S 1×10^{11} vp	Placebo
5	75	Placebo	Placebo
**Cohort 1b (Adults ≥18 to ≤55 years)**			
**Group**	**N**	**Day 1 (Vaccination 1)**	**Day 57 (Vaccination 2)**
1	5	Ad26.COV2.S 5×10^{10} vp	Ad26.COV2.S 5×10^{10} vp
2	5	Ad26.COV2.S 5×10^{10} vp	Placebo

3	5	Ad26.COV2.S 1×10^{11} vp	Ad26.COV2.S 1×10^{11} vp
4	5	Ad26.COV2.S 1×10^{11} vp	Placebo
5	5	Placebo	Placebo
**Cohort 2a (Adults ≥18 to ≤55 years)**			
**Group**	**N**	**Day 1 (Vaccination 1)**	**Day 57**
1-4	120	Ad26.COV2.S 5×10^{10} vp[c]	No vaccination
5	15	Placebo	No vaccination
**Cohort 2b (Adults ≥18 to ≤55 years)**			
**Group**	**N**	**Day 1 (Vaccination 1)**	**Day 57 (Vaccination 2)**
1-4	120	Ad26.COV2.S 5×10^{10} vp	Ad26.COV2.S 5×10^{10} vp
5	15	Placebo	Placebo
**Cohort 3 (Adults ≥65 years)**			
**Group**	**N**	**Day 1 (Vaccination 1)**	**Day 57 (Vaccination 2)**
1	75	Ad26.COV2.S 5×10^{10} vp	Ad26.COV2.S 5×10^{10} vp
2	75	Ad26.COV2.S 5×10^{10} vp	Placebo
3	75	Ad26.COV2.S 1×10^{11} vp	Ad26.COV2.S 1×10^{11} vp
4	75	Ad26.COV2.S 1×10^{11} vp	Placebo
5	75	Placebo	Placebo
**Total**	**1,045**		

In the preliminary report of cohort 1 and 3, for the 805 participants receiving the first dose, frequent solicited adverse events were headache, fatigue, myalgia and injection site pain. Fever occurred more commonly amongst the systemic symptoms. Systemic adverse events were lower in cohort 3 vs. cohort 1 and a similar picture was observed in those receiving a lower dose compared to higher dose. Reactogenicity was lower following the second dose.

In at least 90% of participants, neutralization against the wild type virus was demonstrated on day 29 post-vaccination dose (geometric mean titre [GMT], 224 to 354). This was regardless of age group or dose of vaccine. These titres increase by day and reached 100% by day 57 with additional increase in titres in cohort 1a (GMT, 288 to 488). Titres remained stable until at least day 71. Administration of the second dose, resulted in 2.6 to 2.9 fold increases of titre (GMT 827 TO 1266).

There was no difference between spike-binding antibody responses and neutralizing antibody responses. CD4+ T-cell responses were detected in 76-83% of cohort 1 participants and in 60-70% of those in cohort 3 in day 14. There was skewing toward the type 1 helper T cells. Overall, CD8+ T-cell responses were robust with some attenuation in cohort 3.

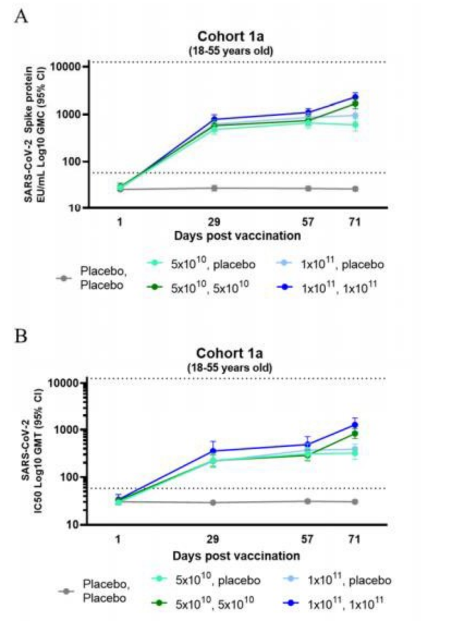

Figure 1. Humoral immunogenicity in cohort 1a

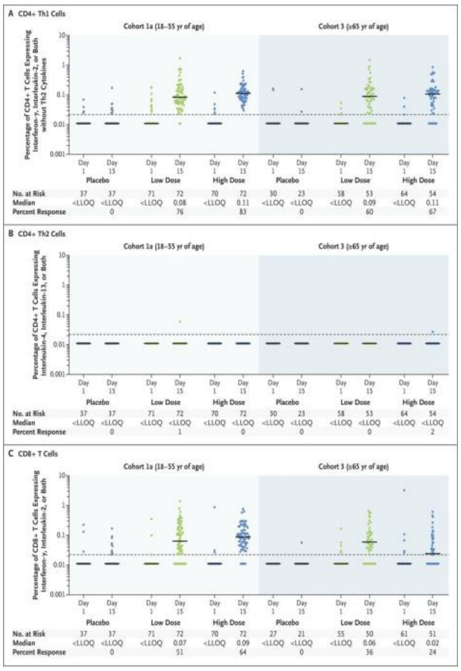

Figure 2. Cellular immunogenicity of Ad26.COV2.S

The single dose of Ad26.COV2.S elicited strong humoral responses in most of the vaccine recipients, including the presence of S-binding and neutralizing antibodies in at least 90% of the participants regardless of age or dose. The increasing and stabilizing antibody titres further point to a durable immune response. These findings, including that of an acceptable safety profile, supported the decision to proceed with two phase 3 trials (Ensemble and Ensemble 2) to evaluate the efficacy of either a single-dose or two-dose regimen of the lower dose (5×10^{10} viral particles) of Ad26.COV2.S.

VAC31518COV3001 – Ensemble (Phase 3)

As of January 2021, approximately 44,000 adult participants had received a single-dose of Ad26.COV2.S in the Ensemble Phase 3 trial conducted across four continents (approximately 6,600 in South Africa). Ensemble is a randomized, double-blind, placebo-controlled phase 3 study to assess the efficacy and safety of Ad26.COV2.S for the prevention of SARS-CoV-2-mediated COVID-19 in adults aged 18 years and older.

Participants were randomized in parallel in a 1:1 ratio to receive intramuscular (IM) injections of Ad26.COV2.S or placebo. Ad26.COV2.S was administered at a dose level of 5×10^{10} vp. The trial is fully enrolled.

The primary objective is to demonstrate the efficacy of Ad26.COV2.S in the prevention of molecularly confirmed, moderate to severe/critical coronavirus disease-2019, as compared to placebo, in SARS-CoV-2 seronegative adults. For the primary objective, all moderate to severe/critical COVID-19 cases are considered. As a secondary objective, vaccine efficacy in the prevention of asymptomatic SARS-CoV-2 infection and mild COVID-19 is analysed. An immunologic test for SARS-CoV-2 seroconversion (ELISA and/or SARSCoV-2 immunoglobulin assay) based on SARS-CoV-2 N protein, is being performed to identify cases of asymptomatic infection. This assay is performed on samples obtained at Day 1 (pre-vaccination), Day 71, 6 months, and 1 year after vaccination.

A total of approximately 400 participants form the Immunogenicity Subset (i.e., 400 participants at sites with access to appropriate processing facilities), blood is collected for analysis of humoral immune responses at Day 1 (pre-vaccination), Day 29, Day 71, 6 months, 1 year, 18 months, and 2 years after vaccination.

The first 2,000 participants in each of the two age groups form the part of the safety subset and remained under observation at the study site for at least 30 minutes post-vaccination to monitor for the development of acute reactions.
The trial is ongoing and at the time of writing, preliminary results showed the single-dose vaccine candidate had an acceptable safety profile and was found to demonstrate 66% effectiveness overall against in preventing moderate and severe COVID -19 disease, as of 28 days after vaccination globally (72% in the USA and 57% in South Africa). It was 85% effective overall in preventing severe disease, and there were no COVID-19 related hospitalizations and deaths, including in South Africa. Importantly, there was a high level of protection observed against severe disease caused by the SARS-CoV-2 variant from the B.1.351 variant lineage observed in South Africa (89% as of 28 days). This single - dose vaccine candidate is estimated to remain stable for two years at -20°C, at least three months of which can be at temperatures of 2-8°C.

VAC31518COV3009 Ensemble 2 (Phase 3)

This is a multi-centre, randomized, double-blind, placebo-controlled, Phase 3, pivotal efficacy and safety study in adults ≥18 years of age. The efficacy, safety, and immunogenicity of Ad26.CoV2.S is being evaluated in participants living in, or going to, locations with high risk for acquisition of SARS-CoV-2 infection after administration of 2 doses of study vaccine.
Even though a single-dose regimen in study VAC31518COV1001 showed good immunogenicity that may already protect against COVID-19, data from other vaccines using the Ad26 platform suggest that a second vaccination may potentially result in an increased and more durable immune response, providing justification for the evaluation of a 2-dose regimen in this study.
Participants will be randomized in parallel in a 1:1 ratio to receive IM injections of Ad26.COV2.S or placebo as shown in the table below. Ad26.COV2.S will be administered at a dose level of 5×10^{10} vp. Endpoints are largely similar to the 1 dose Ensemble trial.

Table 2: Vaccination Schedule VAC31518COV3009

Group	N	Day 1	Day 57
1	15,000	Ad26.COV2.S (5×10^{10} vp)	Ad26.COV2.S (5×10^{10} vp)
2	15,000	Placebo	Placebo

Note: It is intended that a minimum of approximately 30% of recruited participants will be ≥60 years of age and approximately 20% of recruited participants will be ≥18 to <40 years of age
At the time of writing this protocol, the trial is currently ongoing.

2.6 Clinical safety in post-licensure roll out.

On the 13 April 2021 the US based Food and Drug Administration and the Centre for Disease Control issued a recommendation to pause the national roll out of the JnJ vaccine. It was stressed in a briefing (An archive of the briefing can be found here) that the pause was recommended out of an abundance of caution. While it was reiterated that there have only been 6 cases out of nearly 7 million doses of JnJ vaccine administered, the individualized treatment approach required and the similarities to the AstraZeneca COVID19 vaccine (Vaxevria) adverse events previously reported (see below) led to the decision to request a pause.
All 6 cases have occurred in young women under the age of 50 years.
Detail of cases:

- Median age 33 years (18-48 years)
- Median time to symptom onset: 8 days (6-13 days)
- All cases occurred in white females
- Current oestrogen/progesterone usage (n=1)
- Nil pregnant or post-partum
- Pre-existing condition:
- Obesity(3); hypothyroidism (1); Hypertension (1); Asthma (1); hypercoagulation disorder (0)

Initial and late symptoms among 6 CVST patients post JnJ vaccination:

	Initial features	Late features
Patient 1	Headaches, lethargy	Severe headache, left-sided weakness, vomiting
Patient 2	Headaches	Severe headache, aphasia
Patient 3	Headaches, vomiting, fever	Left arm weakness, right gaze deviation, left neglect
Patient 4	Headaches, chills, myalgias	Severe abdominal pain and fever
Patient 5	Headache, chills, dyspnea, fever	Bruising, unilateral leg swelling, loss of consciousness
Patient 6	Back pain, bruising	Headache, abdominal pain

*All were hospitalized and admitted to the intensive care unit

17

This prompted a similar cautionary pause in the Sisonke 3B study as recommended by human research ethics committees at UCT and UKZN and the SAHPRA, and announced publcally by the Minister of Health. The Sisonke leadership initiated a study pause at 20:00 hours on the 13th of April 2021. This was done to allow further collection of information on the 6 reported cases and to create and submit an amended Sisonke protocol to SAHPRA and the related human research ethics committees. This amendment also includes updates to the ICF, PIS and a dear participant letter (DPL).

Safety information on other Covid19 vaccines:

In a decision on 7 April, the European Medicine Authority's safety committee (PRAC) concluded that a syndrome of unusual blood clots with low blood platelets should be listed as very rare side effects of Vaxzevria (formerly COVID-19 Vaccine AstraZeneca). Despite this exceedingly rare complication, they also confirmed that the overall benefit-risk ratio remained positive in favour of vaccination.

European Medicines Agency Statement (20 April 2021)

At its meeting of 20 April 2021, EMA's safety committee (PRAC) concluded that a warning about unusual blood clots with low blood platelets should be added to the product information for COVID-19 Vaccine Janssen. PRACalso concluded that these events should be listed as very rare side effects of the vaccine.

In reaching its conclusion, the Committee took into consideration all currently available evidence including eight reports from the United States of serious cases of unusual blood clots associated with low levels of blood platelets, one of which had a fatal outcome. As of 13 April 2021, over 7 million people had received Janssen's vaccine in the United States.

All cases occurred in people under 60 years of age within three weeks after vaccination, the majority in women. Based on the currently available evidence, specific risk factors have not been confirmed.

PRAC noted that the blood clots occurred mostly at unusual sites such as in veins in the brain (cerebral venous sinus thrombosis, CVST) and the abdomen (splanchnic vein thrombosis) and in arteries, together with low levels of blood platelets and sometimes bleeding. The cases reviewed were very similar to the cases that occurred with the COVID-19 vaccine developed by AstraZeneca, Vaxzevria.

Healthcare professionals and people who will receive the vaccine should be aware of the possibility of very rare cases of blood clots combined with low levels of blood platelets occurring within three weeks of vaccination.

COVID-19 is associated with a risk of hospitalisation and death. The reported combination of blood clots and low blood platelets is very rare, and the overall benefits of COVID-19 Vaccine Janssen in preventing COVID-19 outweigh the risks of side effects.

EMA's scientific assessment underpins the safe and effective use of COVID-19 vaccines. Use of the vaccine during vaccination campaigns at national level will take into account the pandemic situation and vaccine availability in individual Member States.

One plausible explanation for the combination of blood clots and low blood platelets is an immune response, leading to a condition similar to one seen sometimes in patients treated with heparin called heparin induced thrombocytopenia, HIT.

PRAC emphasises the importance of prompt specialist medical treatment. By recognising the signs of bloods clots and low blood platelets and treating them early, healthcare professionals can help those affected in their recovery and avoid complications. Thrombosis in combination with thrombocytopenia requires specialised clinical management. Healthcare professionals should consult applicable guidance and/or consult specialists (e.g., haematologists, specialists in coagulation) to diagnose and treat this condition.

As for all vaccines, EMA will continue to monitor the vaccine's safety and effectiveness and provide the public with the latest information.

This phenomenon has now been published (NEJM April2021) and further information is available. The clinical picture of moderate-to-severe thrombocytopenia and thrombotic complications at unusual sites beginning approximately 1 to 2 weeks (4-20 days with a median of 9 days) after vaccination against SARS-CoV-2 with ChAdOx1 nCov-19 suggests a disorder that clinically resembles severe heparin-induced thrombocytopenia, a rare but well-known prothrombotic disorder caused by platelet-activating auto-antibodies that recognize multimolecular complexes between cationic PF4 and anionic heparin.
However, unlike the usual situation in heparin-induced thrombocytopenia, these vaccinated patients have not received any heparin to explain the subsequent occurrence of thrombosis and thrombocytopenia.

In recent years, it has been recognized that triggers other than heparin can cause a prothrombotic disorder that strongly resembles heparin-induced thrombocytopenia on both clinical and serologic grounds, including certain polyanionic drugs (e.g., pentosan polysulfate, antiangiogenic agent PI-88, and hypersulfated chondroitin sulfate). Such a prothrombotic syndrome has also been observed in the absence of preceding exposure to any polyanionic medication, such as after both viral and bacterial infections and knee-replacement surgery. These various clinical scenarios with apparent nonpharmacologic triggers have been classified under the term autoimmune heparin-induced thrombocytopenia.

Unlike patients with classic heparin-induced thrombocytopenia, patients with autoimmune heparin-induced thrombocytopenia have unusually severe thrombocytopenia, an increased frequency of disseminated intravascular coagulation, and atypical thrombotic events. When these unusual antibodies are observed in patients who have thrombocytopenia without preceding heparin exposure, the term "spontaneous" heparin-induced thrombocytopenia syndrome has been used.

Clinical features that resemble those of autoimmune heparin-induced thrombocytopenia were observed in the patients with vaccine-induced immune thrombotic thrombocytopenia (VITT). The serum from these cases usually showed strong reactivity on the PF4–heparin ELISA. More strikingly, most serum showed inhibition, rather than increased activation, in the presence of low-dose low-molecular-weight heparin (0.2 U per millilitre of anti–factor Xa). Enhancement of platelet activation by PF4 is also a feature of heparin-induced thrombocytopenia and has been used to enhance detection of platelet-activating antibodies in diagnostic testing for this adverse drug reaction. Whether these antibodies are autoantibodies against PF4 induced by the strong inflammatory stimulus of vaccination or antibodies induced by the vaccine that cross-react with PF4 and platelets requires further study. In addition, which component of the vaccine which may

be causing this clinical phenomenon is also not yet understood. Interactions between the vaccine and platelets or between the vaccine and PF4 could play a role in pathogenesis. One possible trigger of these PF4-reactive antibodies could be free DNA in the vaccine, alternatively it is possible the vector may play a role or indeed other incipients.

The recent NEJM paper concludes: It is now known that venous or arterial thrombosis can develop at unusual sites such as the brain or abdomen, which becomes clinically apparent approximately 5 to 20 days after Astra-Zeneca vaccination. If such a reaction is accompanied by thrombocytopenia, it can represent an adverse effect of the preceding Astra-Zeneca Covid-19 vaccination. ELISA to detect PF4–heparin antibodies in patients with heparin-induced thrombocytopenia is widely available and can be used to investigate patients for potential postvaccination thrombocytopenia or thrombosis associated with antibodies against PF4.

So far, most of the cases reported with AZ vaccine have occurred in women under 60 years of age within 2 weeks of vaccination. Based on the currently available evidence, specific risk factors had not been confirmed; and traditional risk factors for thrombosis e.g., smoking, oral contraceptive use do not seem to be relevant to VITT. The PRAC of the EMA noted that the blood clots occurred in veins in the brain (cerebral venous sinus thrombosis, CVST) and the abdomen (splanchnic vein thrombosis, SVT) and in arteries, together with low levels of blood platelets and sometimes bleeding. The Committee carried out an in-depth review of 62 cases of cerebral venous sinus thrombosis and 24 cases of splanchnic vein thrombosis reported in the EU drug safety database (EudraVigilance) as of 22 March 2021, 18 of which were fatal. The cases came mainly from spontaneous reporting systems of the EEA and the UK, where around 25 million people had received the vaccine.

Prompt specialist care is required early in people with these adverse events.

Diagnosis and management:

Recipients should be aware of the possible association and seek immediate care for signs and symptoms suggestive of thrombocytopenia or thrombotic complications:

- Unremitting, severe headache, occurring during this time frame
- focal neurologic symptoms such as weakness in legs, blurred vision and new onset seizures
- New onset, persistent and unexplained abdominal pain,
- Chest pain, shortness of breath and/or leg pains.

There does not appear to be an association between the vaccine and thromboembolic disorders overall (e.g., pulmonary embolism and deep vein thrombosis).

A full blood count, coagulation screen, and blood film for any patient presenting with acute thrombosis or new-onset thrombocytopenia within 28 days of receiving a COVID-19 vaccination should be ordered. Typical laboratory features include a platelet count $<150 \times 10^9/L$, raised D-dimer levels above the level expected for venous thromboembolism, and inappropriately low fibrinogen.
Antibodies to platelet factor 4 may also be identified.

Avoid platelet transfusions and all forms of heparin, including heparin-based flushes. Correct fibrinogen if needed and then anticoagulated with a non-heparin-based anticoagulant. Corticosteroids and plasma exchange may be required. Intravenous immunoglobulin has also been recommended if available. Consult a haematologist for guidance on diagnosis and management of this condition.

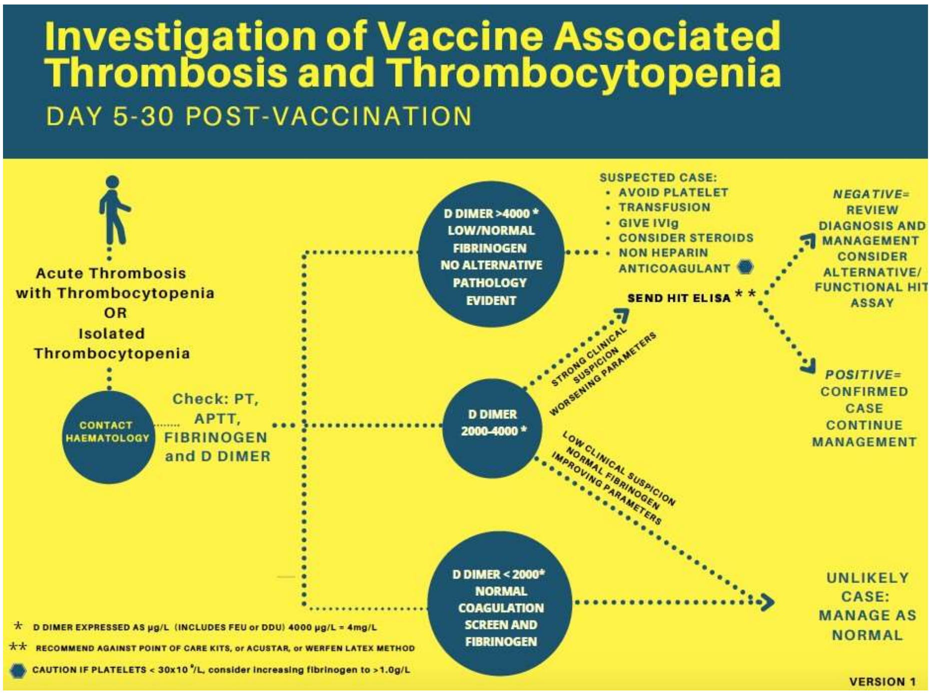

Guidance from the South African Society for Thrombosis and Haemostasis has also now beein published in the SAMJ (19 April 2021) and is available on line..

2.7 Clinical safety on Sisonke 3B study to date (12 April 2021)

At present, we have not seen any cases of unusual clotting associated with low platelet counts, central venous thrombosis, SVT or other major hypercoagulable state among the 289 787 health care workers who have received the JnJ vaccine as of end of Monday 12 April. Thus far 2.2% of healthcare workers who received the JnJ vaccine as part of the Sisonke study reported side-effects or an adverse health event following vaccination. Only 134 people were referred for further evaluation at an emergency room or hospital. Most of these events have been minor, local or systemic reactions. One person experienced a severe allergic reaction that met the international diagnostic criteria for anaphylaxis but has since made a swift and complete recovery.

We have noted some thromboembolic events (Cerebro-vascular accidents and pulmonary emboli) but none of these have been associated with the features described in the clinical syndrome of VITT. All have been investigated, reviewed by the PSRT and additional exeprts and have been deemed unrelated to vaccination.

2.8 Clinical Safety Experience with Ad26 for non-COVID-19 vaccines

As described above, replication-incompetent Ad26 is being used as a vector in the development of vaccine candidates against diseases such as malaria, RSV, HIV, Ebola virus, Zika virus, and filovirus.

As of 01 July 2020, Ad26-based vaccines had been administered to approximately 90,000 participants in ongoing and completed studies, including more than 76,000 participants in an ongoing Ebola vaccine study in the Democratic Republic of the Congo (VAC52150EBL3008/DRC-EB-001) and an ongoing immunization campaign in Rwanda (UMURINZI Ebola Vaccine Study campaign).

The Company's clinical AdVac® safety database report (V5.0, dated 10 April 2020, cut-off date 20 December 2019) describes integrated safety data from 26 completed clinical studies using Ad26-based vaccines for which the database was locked for final analysis. In these 26 studies, 4,224 adult participants were vaccinated with an Ad26-based vaccine and 938 adult participants

received a placebo. A total of 6,004 Ad26-based vaccine doses were administered to adults. Most adult participants (3,557 out of 4,224; 84.2%) received Ad26-based vaccine at a dose level of 5×1010 vp, while 284 adult participants (6.7%) received Ad26-based vaccine at the 1×1011 vp dose level (the highest dose level tested).

As of 01 July 2020, more than 85,000 participants were enrolled in ongoing studies and the ongoing immunization campaign in Rwanda (UMURINZI Ebola Vaccine Study campaign). However, their safety data were not included in the AdVac® safety database report V5.0 because either the studies were still blinded, or the studies were unblinded but their analysis took place after the AdVac® safety database report cut-off date, or the study data were not integrated in the Ad26-based vaccine database used for the report.

Overall, the Ad26-based vaccines were well tolerated irrespective of the antigen transgene, without significant safety issues identified to date.

Ad26-based Vaccines in Adults Aged 60 Years and Older

In the RSV vaccine clinical development program, Ad26.RSV.preF has been evaluated in studies in participants aged ≥60 years, including the Phase 1 studies VAC18193RSV1003 and VAC18193RSV1005, Phase 1/2a study VAC18193RSV1004, Phase 2a study VAC18193RSV2003, and Phase 2b study VAC18193RSV2001. Up to a cut-off date of 24 April 2020, approximately 3,700 participants aged ≥60 years have received an Ad26.RSV.preF-based regimen in completed and ongoing studies. An acceptable safety and reactogenicity profile in participants aged ≥60 years has been reported for the Ad26.RSV.preF-based regimens assessed in these studies, and no safety concerns have been raised to date.

Th1/Th2 Profile of Ad26-based Vaccines in Clinical Studies

In the 1960s, a formalin-inactivated RSV vaccine was associated with enhanced respiratory disease (ERD) in young children, characterized by an increased rate of RSV-mediated, severe lower respiratory tract infection in the vaccinated individuals compared with the control group (4-7). Although the mechanisms for ERD are not fully understood, it is thought that FI-RSV may have:

1) Failed to induce adequate neutralizing antibody titres;
2) Led to an overproduction of binding antibodies promoting immune complex deposition and hypersensitivity reactions;
3) Failed to induce adequate numbers of memory CD8+ T cells important for viral clearance; and
4) Induced Th2-skewed type T-cell response (8).

Vaccine-induced ERD has also been described for SARS-CoV and MERS-CoV in animal models (2), but proof of human SARS-CoV or MERS-CoV vaccine-associated enhanced disease does not exist as these candidate vaccines were never tested for efficacy nor used in outbreak situations. For SARS and MERS, the mechanism of enhanced disease observed in mice has been associated with a Th2-mediated eosinophilic infiltration in the lung, which is reminiscent of ERD effects observed after RSV infection of mice immunized with FI RSV. Similar to RSV vaccines, enhanced disease has been shown for whole-inactivated SARS-CoV vaccines, as well as subunit vaccines inducing a Th2-type immune response, which can be rescued by formulating vaccines in Th1-skewing adjuvants. In addition to a Th1-biased immune response, also induction of a high proportion of neutralizing antibodies compared with virus binding antibodies is desirable to prevent predisposition to enhanced disease as observed for RSV vaccines. While vaccine-associated enhanced disease was observed in nonclinical studies with experimental SARS and MERS vaccines, it is not a given that the same risk applies to COVID-19 vaccines. To the Company's knowledge, antibody-related COVID-19 disease enhancement has not been observed in nonclinical models yet. Antibodies against the receptor-binding domain of SARS-CoV-2 were shown not to enhance in vitro infectivity. Repeated SARS-CoV-2 challenge of NHP or NHP studies with Th2 biasing COVID-19 vaccines that would be expected to predispose to enhanced disease did not show any signs of enhanced disease. In addition, disease enhancement was not

observed in NHP immunized with ChAdOx1 encoding SARS-CoV-2 S protein prior to challenge with SARS-CoV-2.
The immunogenicity profile of adenoviral vectors, with particular emphasis on Th1 responses, is illustrated by data obtained from immunization of adults with Ad26-vectored HIV vaccines (Ad26.ENVA.01 and Ad26.Mos.HIV) and Ad26-vectored Ebola vaccine (Ad26.ZEBOV). These data show predominantly IFN-γ and TNF-α production in $CD4^+$ and $CD8^+$ T cells (9-11). In the RSV vaccine clinical development program, Ad26.RSV.preF is being evaluated in healthy RSV-seropositive toddlers aged 12 to 24 months (Phase 1/2a study VAC18194RSV2001). Safety data from the PA at 28 days after the second study vaccination revealed no safety concerns following Ad26.RSV.preF dosing at 5×10^{10} vp or a placebo. The immunogenicity of a single immunization with Ad26.RSV.preF in RSV-seropositive toddlers aged 12 to 24 months, including favourable Th1 bias, was confirmed. In a further study of Ad26.RSV.preF in RSV-seronegative toddlers aged 12 to 24 months (Phase 1/2a study VAC18194RSV2002), initial safety data have not revealed concerns after Ad26.RSV.preF dosing.

Ad26.COV2.S and pregnant women.

Rationale for inclusion of Pregnant Women in 2nd and 3rd Trimester (from week 16- 34 weeks as reported by antenatal care provider)

There is an increased risk of severe COVID-19 disease during pregnancy, as well as an increased risk of adverse birth outcomes. (Allotey J, BMJ 2021; Ellington MMWR 2020; Collin 2020; Delahoy MMWR 2020, Zambrano MMWR 2020). An ongoing systematic review (Allotey J), details an analysis of the results of 192 studies into the impact of COVID-19 on pregnant women and their babies. The review found that one in 10 pregnant and recently pregnant women attending or admitted to hospital for any reason were diagnosed with confirmed COVID-19. Overall, 339 pregnant women with confirmed COVID-19 died from any cause (0.02% of a total 41,664 women involved in 59 studies). In this review, the overall rates of stillbirth and neonatal death were low in women with suspected or confirmed COVID-19.

The most common clinical symptoms of COVID-19 in pregnant women were fever (40%) and cough (41%), although compared to non-pregnant women of reproductive age, pregnant and recently pregnant women with COVID-19 were more likely to be asymptomatic. Increased maternal age, high body mass index, non-white ethnicity, and pre-existing comorbidity including chronic hypertension and diabetes were identified as risk factors for pregnant women developing severe COVID-19. While there is emerging evidence from the review that pregnancy specific conditions such as pre-eclampsia and gestational diabetes may be associated with severe covid-19, more data are needed to robustly assess the association between pregnancy specific risk factors and COVID-19 related outcomes.

These data confirm that pregnant women should be considered a high risk group, particularly those identified to have risk factors for severe COVID-19. Although pregnancy was initially an exclusion for participation in the Sisonke 3B study, these data suggest that the eligibility for Sisonke 3B should be amended to allow inclusion of pregnant women. Further data to support this is given below:

Summary of the preclinical reproduction and Developmental Toxicity findings:

1. **Fertility and Embryonic Development**

Stand alone fertility evaluations are not routinely required for vaccines. Histopathology data from a (repeated dose) toxicity study are considered to provide sufficient relevant information concerning a possible impact of the vaccine regimen on the integrity of the reproductive organs.

The histopathology data from the repeat-dose GLP toxicity study with Ad26.COV2.S in rabbits do not raise any concerns that the vaccine adversely affects male or female reproductive organs. In addition, the available biodistribution studies did not show distribution and / or persistence of the Ad26 vector in the gonads (testes, ovaries) following IM injection. Female fertility was evaluated as part of the available embryo-fetal and pre- and postnatal development (EF-PPND) toxicity study with Ad26.COV2.S via the premating vaccine administration, and did not show any adverse effects. Based on the above, further dedicated male or female fertility studies are not deemed necessary.

2. **Embryo-Fetal Development**

A GLP-compliant EF-PPND toxicity study was conducted in female, pregnant NZW rabbits to evaluate potential fetal and developmental toxicity following maternal exposure to Ad26.COV2.S on three occasions. Rabbits were injected IM with a control solution (0.9% sodium chloride) or a first dose of 1×10^{11} vp Ad26.COV2.S seven days prior to mating (i.e, Day 1) with untreated males to ensure induction of a maternal immune response during mating and early gestation. In order to evaluate potential direct embryotoxic effects of the components of the vaccine formulation and to ensure high antibody titers during early gestation, a second vaccine dose was administered 6 days after mating (i.e., on Gestation Day [GD] 6), corresponding to the start of organogenesis, around implantation in rabbits. This was followed by a third vaccination on GD20, in order to assess the (direct) effects of the vaccine during late gestation and to ensure a sustained maternal immune response during late gestation as well as during lactation. The study included 2 subgroups: one group consisting of females that were necropsied on GD29 and had a uterine and fetal examination (external, visceral, and skeletal exams), and one group consisting of females that were allowed to give birth and in which the survival and development of the kits were evaluated through Lactation Day (LD) 28. An immunogenicity assessment of maternal and fetal/kit serum was performed to confirm responsiveness of the animals to the vaccine and to assess possible maternal transfer of antibodies.

The vaccine dose, the applied dose volume (1 mL) and the number of doses administered (3) cover the clinical regimen of the vaccine (5×10^{10} vp, administered as a single dose in 0.5 mL). In addition, the EF-PPND study was conducted with the Phase 1/2a clinical Ad26.COV2.S material which is representative for the material that is used in the (Phase 3) clinical studies, as well as for commercial batches. No Ad26.COV2.S related mortality was observed throughout the study. No adverse effects of Ad26.COV2.S were seen on clinical observations, dermal scoring, body weight, body weight gain, food consumption, reproductive performance, fertility, ovarian and uterine examinations, parturition, or macroscopic evaluations in the parental females. In addition, no adverse effects of treatment were seen on fetal body weights, external, visceral, or skeletal evaluations on GD29, or F1 kit evaluations from LD0-28 (sex ratios, survival, body weights, clinical findings, developmental evaluations, and macroscopic evaluations).

All animals immunized with 1×10^{11} vp Ad26.COV2.S on Days 1, GD6 and GD20 and their foetuses had high SARS-CoV-2 S protein-specific antibody titers as measured in serum samples taken on GD29 (in gestation phase subgroup), indicating that maternal antibodies were transferred to the fetuses. Antibody titers of does and fetuses were comparable. Titers measured in does on LD28 and GD28 were comparable (in lactation phase subgroup). Kits also exhibited antibody binding titers on LD28, with the kit titers being 1.3-fold lower than the does.

In conclusion, Ad26.COV2.S administered on three occasions (7 days prior to mating, GD6 and GD20) at 1×10^{11} vp/dose did not induce maternal or developmental toxicity following maternal exposure during the premating and gestation period.

3. **Prenatal and Postnatal Development, Including Maternal Function**

Assessment of pre- and postnatal development was included in the combined EF-PPND toxicity study. In that study, a subgroup of females was administered a first vaccination 7 days prior to mating with untreated male rabbits followed by a second and third vaccination 6 and 20 days after mating (i.e., GD6 and GD20). They were allowed to give birth and raise their offspring

until weaning. The survival and development of the kits were evaluated through LD28 and did not show any adverse vaccine-related effects.

4. Studies in Which the Offspring Are Dosed

Studies in juvenile animals were not performed. The available repeated dose toxicity study with Ad26.COV2.S is considered to provide sufficient assurance of safety regarding possible effects associated with an immune response to the vaccine in infants/children/adolescents. There were no findings in this study that would indicate a concern for the use of the vaccine in infants/children/adolescents. Therefore, no further nonclinical (safety) studies are deemed necessary to support the paediatric development.

5. Local Tolerance

Separate studies to determine local tolerability were not performed. The local tolerance of Ad26.COV2.S was evaluated as part of the pivotal repeated dose toxicity and reproductive toxicity studies. In these toxicology studies, the vaccine formulation was well tolerated when administered at 1×10^{11} vp in 1 mL. No adverse local reactions at the administration sites were noted.

The study designs of planned and ongoing clinical studies in pregnant women:

Phase 2 Open-label clinical studyHealthy pregnant (2nd and/or 3rd trimester of pregnancy) participants ≥18 to ≤45 years of age. To assess the safety and reactogenicity of a single dose level (5×10^{10} vp) of Ad26.COV2.S, administered intramuscularly (IM) as a 2-dose schedule, in adult participants during the 2nd and/or 3rd trimester of pregnancy. The study will assess the humoral immune response in peripheral blood of adult participants, to Ad26.COV2.S at a single dose level (5×10^{10} vp), administered IM as a 2-dose schedule during the 2nd and/or 3rd trimester of pregnancy, 28 days after vaccination.

Breastfeeding: COVID-19 vaccines are not able to replicate in the human body, disintegrate within 2-3 days of vaccination, and do not pass into breastmilk. Breastfeeding women have been included in all trials of the JnJ vaccine thus far, with no safety concerns reported in mothers or their infants.

Further developments to date regarding pregnancy:

Combined developmental and reproductive toxicity studies in rabbits have been reviewed by the FDA, who concluded that Ad26.COV.S given prior to mating and during gestation periods at doses of 1 $X10^{11}$ VP (2 times the human dose) did not have any adverse effects on female reproduction, fetal/embryonal development or postnatal development, further justifies the inclusion of pregnant women in the Sisonke study.

The American college of Obstetricians and Gynecologists' Immunization, Infectious Disease and public Health Preparedness Expert Work Group, the USA Center for Disease Control and the WHO have recommended the JNJ vaccines for use in pregnant and lactating women.
They have concluded that pregnant women and lactating women in the USA can receive the Ad 26.COV2.S vaccine. Replication incompetent or replication defective virus vaccines are not contra-indicated in pregnancy. The same type of vaccine has been authorized for use in Ebola and has been studied extensively for other illnesses.

Although there is an ongoing Phase 2 study that assesses the safety and reactogenicity of the Ad26.COV2.S administered IM as part of a 2 dose schedule (28 days apart) in pregnant women in their 2nd or 3rd trimester (NCT04765384), the FDA EUA allows for its use in pregnancy.

Pregnancy has been reported in 8 participants in Ensemble 1 (4 vaccines and 4 placebo). Vaccination was within 30 days of last menstrual period. Outcomes include spontaneous abortion

(1 vaccine; 0 placebo); incomplete abortion (0 vaccine; 1 placebo); elective abortion (0 vaccine; 2 placebo) and ectopic pregnancy (1 vaccine; 0 placebo).

A recent publication of real world surveillance data in pregnant women receiving the mRNA vaccines has reported safety overall. A total of 35,691 v-safe participants 16 to 54 years of age identified as pregnant. Injection-site pain was reported more frequently among pregnant persons than among nonpregnant women, whereas headache, myalgia, chills, and fever were reported less frequently. Among 3958 participants enrolled in the v-safe pregnancy registry, 827 had a completed pregnancy, of which 115 (13.9%) resulted in a pregnancy loss and 712 (86.1%) resulted in a live birth (mostly among participants with vaccination in the third trimester). Adverse neonatal outcomes included preterm birth (in 9.4%) and small size for gestational age (in 3.2%); no neonatal deaths were reported. Although not directly comparable, calculated proportions of adverse pregnancy and neonatal outcomes in persons vaccinated against Covid-19 who had a completed pregnancy were similar to incidences reported in studies involving pregnant women that were conducted before the Covid-19 pandemic. Among 221 pregnancy-related adverse events reported to the VAERS, the most frequently reported event was spontaneous abortion (46 cases). (NEJM April 21,2021,DOI: 10.1056/NEJMoa2104983)

We acknowledge that safety data for COVID-19 vaccines in pregnancy and breastfeeding are still accumulating. International and local experts and groups such as the College of Obstetricians and Gynaecologists of South Africa, the World Health Organization, the International Federation of Obstetrics and Gynaecologists, the US CDC, the United Kingdom's Joint Committee on Vaccination and Immunisation, the American College of Obstetrics and Gynaecology and the Royal College of Obstetricians and Gynaecologists have remained united in strongly recommending vaccination for pregnant and breastfeeding women given large amounts of safety data for similar ''non-live' vaccines. Pregnancy exposure safety data for >100,000 exposed pregnancies for other 'non-live' vaccines including seasonal influenza and tetanus, diphtheria, pertussis and poliomyelitis polyvalent vaccines show no safety concerns. Animal studies for mRNA and the JnJ vaccine showed no concerns, and the Ebola vaccine, which uses the same adenovirus vector as the JnJ vaccine, has been used widely including pregnant women with no concerns. It is therefore considered highly unlikely that such vaccines would be harmful if administered in pregnancy or breastfeeding.

The Sisonke study will evaluate the number of participants with pregnancy outcomes (including live-births, live preterm birth, still born or abortion). We will also evaluate the number of participants with pregnancy related AEs. For neonates/infants born will be followed up passively through the safety desk.

Pregant women will be eligible to participate in the Sisonke Study after they have consulted with their treating obstetrician or doctor, and after evaluation by the research staff for any underlying illnesses or allergies.

3.0 OBJECTIVES AND ENDPOINTS

Overall aim: To monitor the effectiveness of the single dose Ad26.COV2.S COVID-19 vaccine among health care workers in South Africa.

Objectives	Endpoints
**Primary**	
To assess the effectiveness of Ad26.COV2.S vaccine on severe COVID, hospitalizations and deaths in HCWs as compared to the general unvaccinated population in South Africa	Rates of hospitalizations and deaths among vaccinated HCWs versus general unvaccinated population in South Africa

**Secondary**	
To estimate the incidence of symptomatic and severe SARS CoV-2 infections among vaccinated HCWs	Incidence rate of SARS CoV-2 infection as indicated by self-report and validation in national laboratory records.<br>Rates of severe disease in HCW who are found to be RT-PCR positive at any time up to 2 years post vaccination
To monitor the genetic diversity of breakthrough SARS CoV-2 infections	Genetic diversity of breakthrough infection virus as determined by whole genome sequencing. This will be recovered from national laboratories.
To monitor pre-existing COVID19 immune status in up to 100 000 HCWs at baseline prior to vaccination to measure pre-existing immunity.	Prevalence of SARS CoV-2 seropositivity at baseline
To monitor the immunological responses of HCWs who experience breakthrough SARS CoV-2 infections	Measure the neutralising antibodies, non-neutralising antibodies and T cell immunity in the blood samples of health care workers who have breakthrough COVID-19 infection. This will be measured as soon as possible at the time of infection.
To measure serum neutralization , T-cell responses and certain clotting parameters among vaccinee groups of interest.(approximate sub-set of 250 vaccinees aged 20-55; 250 vaccinees aged >55 years; 250 stable HIV positive vaccinees; 250 with comorbidities, including chronic clotting disorders, 200 pregnant HCW; 200 breastfeeding HCW)	Adverse events, neutralization titres, T cell assays and clotting parameters (DDimers, FBC, Differential, platelets) among vaccinees in groups of interest before and after vaccination
To monitor for asymptomatic infection in a sub-set of HCWs	Rates of asymptomatic infection at baseline and follow up using SARS CoV-2 virus and antibody testing.
To estimate vaccine uptake among HCWs in South Africa	Proportion of HCWs approached for study participation taking part in the study and receiving the vaccine
**Exploratory**	
To conduct pharmacovigilance to monitor for safety and any unexpected adverse effects of the vaccine administration	Numbers of safety events and/or unexpected adverse effects reported to the study team<br>Monitor pregnancies and pregnancy outcomes reported to safety desk.

4.0 STUDY DESIGN

This is multi-centre open-label, single-arm phase 3B implementation study in HCW in South Africa at least 18 years of age. This study will be conducted by Sisonke (VAC31518COV3012) sites in collaboration (where appropriate) with routine National Department of Health vaccination centres in South Africa. All HCWs who register on the National Vaccination Registry will be eligible for enrolment. Participants will receive appointments for vaccination using the registry. Vaccination will be overseen by trained personnel linked to ENSEMBLE trial sites with a follow-up duration of up to 2 years for surveillance. The sub-cohort will be followed up at Month 3 and

6, and will continue surveillance up to 2 years post-vaccination If a participant is unable to complete the study, but has not withdrawn consent, an early exit visit will be conducted. The end-of-study will be considered as at least 6 months and up to 2 years of follow up for the last participant enrolled in the study. Participants will receive IM injection of Ad26.COV2.S at enrolment at a dose level of 5×10^{10} vp. Surveillance for effectiveness may continue for up to 2 years post vaccination.

4.1 Enrolment plan

Considering the large number of participants, the study teams will work in collaboration with the Department of Health (DOH) teams at designated COVID-19 vaccine administration centres. It is anticipated that approximately 500,000 volunteers will be vaccinated within 3 months. Volunteers already registered on the DOH vaccine registration system will be invited to make an appointment, offered to join the study, and sign an electronic informed consent before receiving the vaccine dose. Participants may also approach Sisonke (VAC31518COV3012) sites directly to participate, however they will still be required to register on the national register prior to enrolment.

4.2 Study population

Approximately 500,000 health care workers in the public and private South African health care system. Participants will be 18 years and older, and can be with or without comorbidities including HIV co-infection. Pregnant and lactating women are allowed under specific conditions (see above and below).

Investigators should always use good clinical judgment in considering a volunteer's overall fitness for trial participation. Acute illness will be a reason to defer enrolment. Although unlikely for functioning frontline health workers, some volunteers may not be appropriate for enrolment even if they meet all inclusion/exclusion criteria. Medical, psychiatric, occupational, or other conditions may make evaluation of safety difficult, or may make volunteers unable to comply with the requirements of participation.

4.3 Eligibility criteria

<u>Inclusion criteria</u>

- Age 18 and older
- Health care worker in the private or public service
- Pregnant women between 16 weeks and 34 weeks gestation who have a letter from their provider or antenatal care service.
- Participants who report breastfeeding at the time of enrolment may be included.
- The President and Deputy President of South Africa *
- Willingness and ability to comply vaccination plan and other study procedures.
- Capable of giving electronic or personal signed informed consent as described in Appendix 5, which includes compliance with the requirements in this protocol.

Inclusion criteria for the sub-cohort

- Age 18 and older
- Health care worker in the private or public service
- Pregnant women who are between 16-34 weeks gestation, have a letter from their provider or antenatal care service and have signed specific consent
- Participants who report breastfeeding at the time of enrolment may be included.
- Willingness and ability to comply with all scheduled visits, vaccination plan, laboratory tests, and other study procedures, with follow-up at an ENSEMBLE site.
- Capable of giving electronic or personal signed informed consent as described in Appendix 5, which includes compliance with the requirements in this protocol.

Exclusion criteria

- Any significant acute or chronic medical condition, situation or circumstance that in the opinion of the PI/designee makes the participant unsuitable for participation in the study, or jeopardises the safety or rights of the participant
- Participants who report being pregnant <16 weeks gestation at time of enrolment, planning conception within 3 months, or beyond 34 weeks gestation.
- Current participation in any other research studies that would interfere with the objectives of this study. The determination of whether participation in another study would be exclusionary for a given participant will be made by the PI/designee.
- History of severe adverse reaction associated with a vaccine and/or severe allergic reaction (e.g., anaphylaxis) to any component of the vaccine.
- Participants who have experienced major venous and arterial thrombosis occurring with thrombocytopenia following vaccination with any COVID-19 vaccine should not enrol in Sisonke.
- Participants with a history of heparin-induced thrombocytopenia.

Note:

- Vaccination within 14-90 days with other COVID19 or non-specific vaccines are not exclusionary but should be discussed with study PI or designee.

Conditions of interest:
We note that international reports of VITT have not identified a risk factor, nor does there appear to be any prothrombotic state that indicates a risk factor for this immune response.
Nevertheless, the Sisonke study will enrol participants with chronic history of severe clotting disorders **only** after consultation and approval of the study Protocol Safety Review Team (PSRT). (See process below).

We have identified certain specific conditions of special interest such as:

- cerebral venous sinus thrombosis,
- antiphospholipid syndrome
- Individuals on therapeutic anticoagulants for current or previous arterial or venous thrombosis or embolism.

*The President and Deputy President of South Africa have been included in the protocol to address the issue of vaccine hesitancy.

5.0 CLINICAL PROCEDURES

Research staff who worked on the ENSEMBLE study (Clinical Protocol VAC31518COV3001) will work in collaboration with local routine designated vaccination sites. Sisonke (VAC31518COV3012) study staff will support and train staff at designated vaccination sites on conduct study procedures in a standardised manner as specified in the study specific procedures (SSP) manual and standard operating procedures (SOPs) documents. Assessment of safety will include evaluation of medical history, a physical assessment only if required and laboratory if appropriate. The study schedules are provided in Appendix 1, 2 and 3. The total blood volume drawn from a subset of participants will comply with regulatory guidelines. The protocol is designed to be "light touch" and as near to real-world vaccination roll out as possible.

5.1 Recruitment

Recruitment will take place through the National Department of Health's electronic vaccine data system (EVDS). Health care workers who voluntarily register on the EVDS will be given a date and time for vaccination at the designated vaccination facility. If possible, an eligibility assessment, patient information leaflet and consent form will be shared with each potential study participant before their appointment date, for completion and submission to the study team. Where electronic informed consent is not possible, a hard copy will be available and will be completed

by the potential study participant when they present for their appointment.

5.1.1 Participants of special concern regarding VITT:

We note that international reports of this condition have not identified a risk factor for the Vaccine Induced Thrombocytopenic Thrombosis (VITT) condition, nor does there appear to be any prothrombotic state that indicates a risk factor for this immune response. Nevertheless, the Sisonke study will do the following to identify participants at high risk of blood clotting disorders:

a. Extensive public messaging pamphlets and posters will be made available at the vaccination sites and will also be available on social media platforms and the SAMRC website.
b. The study Participant Information Sheet (PIS) contains information designed to create awareness about VITT and requests all participants to identify any pre-existing condition that may indicate high risk for clotting disorders.
c. At the time of vaccination, the re-trained vaccinators will focus on asking participants if they have a history of key risk factors, namely high risk for severe allergies (the other key condition of interest) and clotting disorders.
d. These individuals will be referred by the **site Principal Investigator** (PI) associated with the vaccine centre to the Protocol Safety Review Team (PSRT) who will evaluate the participant prior to vaccination.
e. Where needed, the PSRT will seek further information from the site PI or other experts on the protocol team.
f. An email will be sent to the site PI on the decision of the PSRT and the participant will be recalled once a vaccination plan is in place.

We have identified certain specific conditions of special interest such as:

- cerebral venous sinus thrombosis,
- antiphospholipid syndrome
- chronic therapeutic anticoagulation.

These individuals will be offered vaccination and will be closely monitored by the site PI and team under the guidance of the PSRT. Depending on the post vaccination plan, the research site staff will make contact with the participant regularly in the first month and provide participants with a letter giving guidance to possible signs and symptoms as well as emergency contact numbers. We have an undertaking from the Southern African Society of Haemostasis and Thrombosis to support the management of these participants. Please refer to our Standard Operating Procedure for the referral and management of suspected VITT, which is included as an attachment to the V4.1 protocol.

5.2 Screening evaluations

All screening evaluations will take place when the potential participant presents for their vaccination visit. Screening will assess whether the potential participant meets the inclusion criteria for the study. Eligibility will already have been confirmed.

5.3 Enrolment Visit

In this study, enrolment is defined as the receipt of the single-dose vaccination. The participant will be assigned to a participant identification number (PID) per site which will be the national ID or passport number. This will enable a log of participants per site and per province. All clinical procedures and sample collections will take place as per the schedule of evaluations (SOE). Eligibility to enrol will be assessed at the vaccination visit to confirm eligibility to qualify for study vaccine administration. Day 0 will be defined as the day of study vaccine administration. All vaccinated participants will be entered into a national COVID-19 vaccination register.

5.4 Administration of study product/s

The single-dose vaccine will be administered to all participants. The vaccine will be administered either in the left or right deltoid as a IM injection.

5.5 Follow-up procedures

5.5.1 Surveillance for hospitalization and breakthrough infections

All COVID-19 hospitalisations nationally will be linked with the vaccination register fortnightly to identify whether vaccinated participants have had breakthrough infections. Each breakthrough infection will be investigated to determine the viral genome, severity of infection and outcome. This will be done by saving the original nasal swab specimen and shipping the swab to the national laboratories who conduct these tests. Medical records and routine laboratory results will be reviewed.

5.5.2 Sub-cohort (approximately 1000-1400 volunteer HCWs) follow up visits

All follow up visits will take place as per the SOE – there will be one scheduled study follow-up visit at 6 weeks and 6 months in a sub-sample of participants including key sub-populations (approximately 250 vaccinees aged 20-55; 250 vaccinees aged >55 years; 250 stable HIV positive vaccinees; 250 people with co-morbidities 200 pregnant HCW; 200 breastfeeding HCW).

Participants will be encouraged to contact the vaccination centre in the case of any adverse events. A subset (approximately 1000 -1400) HCWs will participate in the sub-study. This will include a blood draw at vaccination for SARS-CoV-2 antibodies at baseline and at follow up visits. These individuals will also be asked about vaccine tolerability and side effects related to vaccination.

Unscheduled follow-up visits or contact,eg. in hospitalised participants will be triggered when a participant has any symptoms of COVID-19. This includes any of the following, as defined in the ENSEMBLE protocol:

New onset or worsening of any 1 of the symptoms, which lasts for at least 24 hours, not otherwise explained:

- Headache
- Malaise (appetite loss, generally unwell, fatigue, physical weakness)
- Myalgia (muscle pain)
- Chest congestion
- Cough
- Runny nose
- Shortness of breath or difficulty breathing (resting or on exertion)
- Sore throat
- Wheezing
- Eye irritation or discharge
- Chills
- Fever (≥38.0°C or ≥100.4°F)
- Pulse oximetry value ≤95%, which is a decrease from baseline
- Heart rate ≥90 beats/minute at rest, which is an increase from baseline
- Gastrointestinal symptoms (diarrhoea, vomiting, nausea, abdominal pain)
- Neurologic symptoms (numbness, difficulty forming or understanding speech)
- Red or bruised looking toes
- Skin rash
- Taste loss or new/changing sense of smell
- Symptoms of blood clots: pain/cramping, swelling or redness in your legs/calves

- Confusion
- Bluish lips or face
- Clinical suspicion/judgement by investigator of symptoms suggestive for COVID-19
- During unscheduled follow-up visits, a swab will be taken to detect SARS-CoV-2 as per routine protocol from the National Institute of Communicable Diseases/National Health Laboratory Services (see schedule of events and specimen collection).

If they are unable to return to the site with any symptoms or confirmed COVID19 diagnosis they will be encouraged to see their own health care practitioners for care, but are also requested to let the relevant research site know. They may also be contacted by the Sisonke safety team via telephone or SMS to encourage feedback on side effects, COVID19 infection, hospitalisation or related issues. They will be approached for a history of clinical symptoms and signs as well as blood and nasal swab specimens.

Outcomes will be defined as per FDA guidelines (accessible at https://www.fda.gov/media/137926/download)

Symptomatic COVID-19

- Positive testing by standard RT-PCR assay or Antigen or equivalent testing with symptoms.
- No clinical signs indicative of severe or critical severity

Severe COVID-19

- Positive testing by standard RT-PCR assay or an equivalent test including antigen test and possibly viral load.
- Symptoms suggestive of severe systemic illness with COVID-19, which could include any symptom of moderate illness or shortness of breath at rest, or respiratory distress
- Clinical signs indicative of severe systemic illness with COVID-19, such as respiratory rate ≥ 30 per minute, heart rate ≥ 125 per minute, SpO2 ≤ 93% on room air at sea level or PaO2/FiO2 < 300
- No criteria for Critical Severity

Critical COVID-19

- Positive testing by standard RT-PCR assay or equivalent test including antigen test and possibly viral load.
- Evidence of critical illness, defined by at least one of the following:
    - Respiratory failure defined based on resource utilization requiring at least one of the following:
        - Endotracheal intubation and mechanical ventilation, oxygen delivered by high flow nasal cannula (heated, humidified, oxygen delivered via reinforced nasal cannula at flow rates > 20 L/min with fraction of delivered oxygen ≥ 0.5), non-invasive positive pressure ventilation, ECMO, or clinical diagnosis of respiratory failure (i.e., clinical need for one of the preceding therapies, but preceding therapies not able to be administered in setting of resource limitation)
    - Shock (defined by systolic blood pressure < 90 mm Hg, or diastolic blood pressure < 60 mm Hg or requiring vasopressors)
    - Multi-organ dysfunction/failure

Safety events/unexpected adverse events will be monitored passively through several systems:

- a vaccination card will be provided to participants, with a telephone number. Participants will be asked to call the number of they experience any adverse events

- the EVDS and other systems will be used to track safety events / adverse events, through a linked pharmacovigilance system (see Clinical Safety section)
- a patient alert card will be provided to participants separately or as part of the vaccination card, with a helpline number on the card for reporting safety /adverse events
- Volunteers will be sent SMSs on Day 2, 7 and 14 as reminders to notify side effects, hospitalisations or COVID 19 infection (see safety section as well).

5.5.3 Clinical research site oversight in Sisonke

- The Clinical research sites approved to conduct the Sisonke Open label study retain overall responsibility for the receipt, management and handling of the investigational product, namely the Ad26.COV2.S Covid19 vaccine.
- Each clinical research site PI manages a site team which is delegated to ensure the study is run according to protocol.
- The Sisonke clinical trial sites have inadequate staff on their own to vaccinate the number of health care workers that must be reached urgently to meet the objectives of the study. For this reason, the Sisonke 3B study is embedded within the national vaccine roll out study and a robust partnership exists between the clinical research site teams and the designated vaccine centers.
- Each vaccine centre has an associated clinical research site, with a site PI who takes overall responsibility for the conduct of Sisonke 3B study at that site.
- The sites approved by SAHPRA have been carefully mapped geographically to link with a series of pre-identified vaccine centres. In the first tranche of vaccine deliveries, vaccine centres were opened in only big sites in metros in each of the 9 provinces.

Research Sites	Province
Aurum Institute Klerksdorp CRS	North West
Aurum Institute Rustenburg CRS	North West
CAPRISA eThekwini CRS	KwaZulu-Natal
Chatsworth CRS	KwaZulu-Natal
Clinical HIV Research Unit (CHRU)	Gauteng
Elandsdoorn CRS	Limpopo
FAM-CRU (Family Clinical research Unit)	Western Cape
Groote Schuur HIV CRS	Western Cape
Josha Research CRS	Free State
Mzansi Ethical Research Centre	Mpumalanga
Nelson Mandela Academic Research Unit	Eastern Cape
Phoenix Pharma	Eastern Cape
Qhakaza Mbokodo Research Clinic CRS	KwaZulu-Natal
Soweto HVTN CRS	Gauteng
Synexus SA – Watermeyer	Gauteng
Wits RHI Shandukani Research Centre	Gauteng

Eighteen (18) public sector hospitals/vaccination centres were identified for the first two-week period and an additional 32 sites for weeks 3 and 4.
Sisonke (Ensemble 1 and 2) Research sites were approved as Primary Distribution Sites receiving product from Biovac. The Vaccination centres were chosen based on proximity to the identified Clinical Research Sites. Some Research sites were either within the grounds of the public sector hospital complex or within 1km.

Subsequently, further vaccination centres have been added but always with due regard for the Clinical research sites, in some cases vaccination centres have been phased out or are operating on a periodic rostered system to ensure sufficient oversight and input from the CRSs. In most cases a CRS is managing one or two vaccination centres at any time. Currently 51 sits are actively engaged with associated clinical research sites and research teams.

Full list of currently operational vaccination centres and associated clinical research sites to date:

**Vac Site Province**	**Vaccination Site**	**CRS**
Eastern Cape	Cecilia Makiwane Hospital	CHRU (CMH)
Eastern Cape	ec Livingstone Hospital	Phoenix Pharma
Eastern Cape	ec Dora Nginza Hospital	Phoenix Pharma
Eastern Cape	Netcare Greenacres	Phoenix Pharma
Eastern Cape	ec Sir Henry Elliot Hospital	NeMACRU
Free State	FS + Josha have agreed on a consolidated plan in both urban & rural settings	Josha Research
Free State	Tokollo Hospital	CRISMO (Tokollo)
Free State	Parys Hospital	CRISMO (Parys)
Free State	Hospitals: Fezi Ngumbentombi Provincial, Boitumelo Regional, Mafube	
Gauteng	Ahmed Kathrada Private Hospital (Lenmed)	PHRU
Gauteng	gp Chris Hani Baragwanath Hospital (to vaccinate Sebokeng & Leratong)	PHRU
Gauteng	Mediclinic Medforum	Synexus Watermeyer
Gauteng	Netcare Milpark Hospital	CHRU
Gauteng	Helen Joseph Hospital	CHRU
Gauteng	gp Charlotte Maxeke Hospital (includes doses for Tembisa/Pholosong/Bertha Gxowa)	WRHI
Gauteng	gp Dr George Mukhari Hospital	MeCRU
KwaZulu-Natal	kz Ladysmith Hospital	Qhakaza Mbokodo
KwaZulu-Natal	kz Dundee Hospital (in	Qhakaza Mbokodo
KwaZulu-Natal	Vreyheid Hosp	Qhakaza Mbokodo
KwaZulu-Natal	Mediclinic Pietermaritzburg Hosp	Vulindlela Botha's Hill
KwaZulu-Natal	Netcare St. Augustine's Hospital	CAPRISA eThekwini
KwaZulu-Natal	Ngwelezane Hospital	MRC Tongaat
KwaZulu-Natal	Hlabisa Hosp	MRC Tongaat
KwaZulu-Natal	For a public hospital in KZN	MRC Chatsworth
Limpopo	Decide allocations locally	Elandsdoorn
Mpumalanga	Decide allocations locally	MERC
North West	Decide allocations locally	Aurum Rustenburg
North West	Joe Morolong Memorial Hospital	Aurum Klerksdorp (Vryburg)
North West	nw Mahikeng Provincial Hospital	Aurum Klerksdorp

Northern Cape	nc Robert Mangaliso Sobukwe Hospital	CHRU-Kimberley
Northern Cape	Kuruman Lenmed Hospital	CHRU-Kathu
Northern Cape	nc Dr Harry Surtie Hospital	CHRU-Upington
Western Cape	Gatesville Medical Centre	DTHF Emavundleni
Western Cape	wc Worcester Hospital	SATVI
Western Cape	wc George Hospital	TASK Eden
Western Cape	wc Groote Schuur Hospital	Groote Schuur DTHC
Western Cape	wc Khayelitsha Hospital	Khayelitsha CRS / CIDRI
Western Cape	wc Paarl Hospital	TASK Central (Paarl)
Western Cape	wc Caledon	Synexus Helderberg
Western Cape	wc Tygerberg Hospital	FAM-CRU
Western Cape	wc Mitchells Plain District Hospital	DTHF Emavundleni

In each of the above, there is a designated CRS PI who takes overall responsibility for the vaccination centre in their jurisdiction. They have dedicated teams and staff who work at the vaccination centres or thw CRS sites to conduct Sisonke/.
This is managed with regular communication with the provincial vaccination team and in partnership with the facility manager/vaccination centre manager at the VC.
The Sisonke leadership have a weekly team meeting with all site PIs and senior investigators.
The pharmacists have regular calls with the Sisonke pahramcy team.
When required, a site PI will join the PSRT calls and have regular interactions with the Sisonke safety team.

Vaccination centre (VC) initiation:
The CRS team meets with the VC team and ensures that all the necessary components are in place for an operational VC including trained administrators, trained and registered vaccinators, IT hardware, adequate spacing to ensure social distancing and queueing and adequate space for post vaccination observation space and resuscitation equipment. If vaccine has to be stored on the vaccine site (e.g. due to distances to travel between CRS and VC) , the pharmacist of record ensures the infrastructure to do so safely is met, e.g. refridgeration, refridgeration electricity back up and security.

Vaccine administration:

- Only adequately trained and approved staff may handle the vaccine or fill the vaccine syringes (see below).
- Research oversight is maintained at the vaccine centre daily through the following actions
- A senior research site person is identifiable and at the vaccine centre daily
- The filled syringes are brought in under carefully controlled conditions (temp controlled, chain of custody). Vaccine doses are quality assured before being handed out to trained and registered vaccinators.

- Vaccinees/participants are screened at the entrance to the vaccine centre for acute COVID and whether they have been registered on the Electronic vaccine data system, have e-Consented to Sisonke and have a vaccine voucher. They are also asked for some other identification as health care worker. They are confirmed electronically on the system. Participants have already been notified through the PIS to the concern around pre-existing conditions of interest.
- **They are then further screened by the vaccinator for any clinical or other eligibility concerns. Should any be raised the CRS researcher is notified. This is a critical step for identifying any chronic conditions of special interest. The notified investigator will then adjudicate the eligibility to enrol the participant or escalate the decision to a central counsel with the PSRT.**
- If none, the participant is vaccinated. After the vaccinator has confirmed the volume in the syringe. The Vaccine voucher number is recorded against that dose of vaccine and vaccine name and lot is recorded on a vaccination card. The person is issued their card, told again what to expect and given details about reactogenicity and the safety number to call if needed. They are also directed to an online adverse event reporting line (see safety plan).
- They are asked to remain for 15 minutes or longer if there are concerns of prior allergy history.
- **Any adverse events are managed by the vaccination staff and the clinical research site staff and further notification is submitted to the safety desk. Any adverse events coming in through the EVDS are also picked up and followed up by the CRS staff and the Sisonke safety desk staff together with the PSRT.**

6.0 LABORATORY PROCEDURES

The following Protocol-Required Laboratory Assessments will be performed in accordance with the schedule of activities in the sub-cohort:

Laboratory Assessments	Parameters	Time points
Testing done	(1) Nasal swabs for virology testing (molecular confirmation Of SARS-CoV-2)	At time of break through infection
	(2) Sub-set of participants (approx. 1000-1400 vaccinees) Blood samples for neutralization assays and immune responses at week 0, 6 and month 6; as well as clotting parameters(DDimers, PF4, FBC +Diff platelets) at weeks 0, 1 and 3. A breastmilk sample will also be taken from breastfeeding HCW at weeks 0, 6 and 6 months	On day of vaccination and follow up at 1,3, 6 weeks and 6 months<br>• 250 healthy 18-60 HCW<br>• 250 HIV infected HCWs<br>• 250 HCW over 60 years<br>• 250 HCW with co-morbidities<br>• 200 pregnant HCWs<br>• 200 breastfeeding HCWs
	(3) RNAseq blood sample for exploration of biomarkers correlating with SARS-CoV-2 infection and COVID-19 severity in participants with breakthrough infection (PAXgene tubes, whole blood)	As soon as possible after identification of the breakthrough infection<br>Samples collected during a confirmed COVID-19 episode. Viral load testing in vaccinees with confirmed SARS CoV-2S infection
	(4) Nasal swab SARS CoV-2 PCR and	Samples collected during a confirmed COVID-19 episode. Viral load testing in

	quantitative viral load SARSCoV2 testing<br><br>(5) Baseline SARS CoV-2S antibody testing in up to 100 000 vaccines Blood samples for neutralization assays and immune responses	vaccinees with confirmed SARS CoV-2S infection<br><br>At Baseline

7.0 STUDY PRODUCT

Ad26.COV2.S will be supplied at a concentration of $2x10^{11}$ vp/ml in vials with an extractable volume of 0.5 ml, and dosed at $5×10^{10}$ vp. Each vial contains 2 doses. (p.4 of IMPA (Investigational Material Packaging Agreement VAC31518COV3012_V1 update_FD-WR.docx). Study vaccine will be administered by IM injection into the deltoid muscle, preferably of the non-dominant arm. If an injection cannot be given in the deltoids due to a medical or other contraindication use alternative locations such as the hip, thigh or buttocks (to be avoided in overweight participants). Study vaccine administration must be captured in the EVDS. Ad26.COV2.S will be manufactured and provided under the responsibility of the Company. Refer to the IB for a list of excipients (2).

Refer to the study site Investigational Product Preparation Instructions/Instructions for Use (IPPI/IFU) for additional guidance on study vaccine administration.

7.1 Preparation, Handling and Storage

All study vaccine must be stored in a secured location with no access for unauthorized personnel and at controlled temperatures as indicated on the clinical labels. If study vaccine is exposed to temperatures outside the specified temperature range, all relevant data will be sent to the Company to determine if the affected supplies can be used or will be replaced. The affected study vaccine must be quarantined and not used until further instruction from the Company is received.

Refer to the study IPPI for additional guidance on study vaccine preparation, handling, and storage.

A study-site pharmacist, or other qualified individual will prepare the appropriate vials and syringes, labelled with the participant's identification number, and provide the filled syringes for the study to the vaccine administrator (a trained and qualified study nurse, medical doctor, otherwise qualified HCP) who will perform the injection.

7.2 Study product accountability

The investigator is responsible for ensuring that all study vaccine received at the site is inventoried and accounted for throughout the study. The study vaccine administered to the participant must be documented on the vaccine accountability form. All study vaccine will be stored and disposed of according to the Company's instructions. Study-site personnel must not combine contents of the study vaccine containers.

Study vaccine must be handled in strict accordance with the protocol and the container label and must be stored at the study site in a limited-access area or in a locked cabinet under appropriate environmental conditions.

Potentially hazardous materials containing hazardous liquids, such as needles and syringes should be disposed of immediately in a safe manner and therefore will not be retained for vaccine accountability purposes.

Study vaccine should be dispensed under the supervision of the investigator or a qualified member

of the study site personnel, or by a hospital/clinic pharmacist. Study vaccine will be administered only to participants participating in the study. Returned study vaccine must not be dispensed again, even to the same participant. Further guidance and information for the final disposition of unused study vaccine are as provided by the Company.

8.0 CLINICAL SAFETY

Phase 1/2a and Phase 3 studies have shown that this vaccine is generally safe to be administered including in the South African population. This cohort study will gather as much data as possible on adverse events by both active and passive means.

Pre-vaccination safety:

Potential participants who identify themselves as high risk for events (previous severe clotting disorders or history of severe allergy or anaphylaxis) should report to the study research site associated with their vaccine centre. This is requested at the time of consenting and is also publicised in a number of patient facing materials. E.g. Informed Consent Form (ICF) and PIS. We have specifically added chronic medical conditions associated with clotting disorders. Once self-identified, these potential participants will be referred to the associated site investigator for assessment of eligibility. These cases will be also be referred via the site PI to the PSRT for central counsel to decide on the following:

- Whether the individual is fit for vaccination
- Where the vaccination should be administered
- The specific conditions under which the vaccine should be administered and
- Specific monitoring post vaccination that may be indicated.

The individual will only be considered eligible for enrolment with the explicit approval of the PSRT. This will be documented via an email exchange with the PSRT.

Post vaccination safety:

1. All participants are encouraged to report their adverse events at the Sisonke desk at vaccination visit, in response to SMSs and on invitation at back of vaccination card. The 24/7 call number appears on the ICF/PIS/Back of the vaccination card and in widespread public announcements and posters/leaflets.
2. An SMS is also sent out at the time of vaccination to remind participants and to link participants to the adverse event online reporting system.
3. Vaccinators and site staff will also use the EVDS or paper based adverse event reporting system to log any adverse events at the time of vaccination.
4. As part of this amendment, additional SMS messages will be sent out weekly twice more to further solicit any adverse events.
5. Participants are asked to report to the safety desk should any event up to 2 years after vaccination occur. (see below for more details)
6. Any suspected VITT case will be referred to the PSRT immediately.
7. Any other case meeting the definition of serious adverse event will be notified to PSRT within 24 hours.
8. PSRT may be convened at any time urgently
9. PSRT meet weekly at a minimum.
10. Hospitalisations and deaths are tracked through the ongoing surveillance platforms of hospital admissions and death registries through the DATCOV system of registered

hospitals and using the unique identifier. Line listings from this database are checked daily.

11. A subset of participants (1000-1400) will be followed up at weeks 1,3 and 6 and 6 months for a targeted assessment. Those with a diagnosis of SARS-CoV-2 are encouraged to contact the study team and will be assessed further. The study team will also link in with the government pharmacovigilance team who will track reported adverse medical events suspected to be related to the vaccine administration.

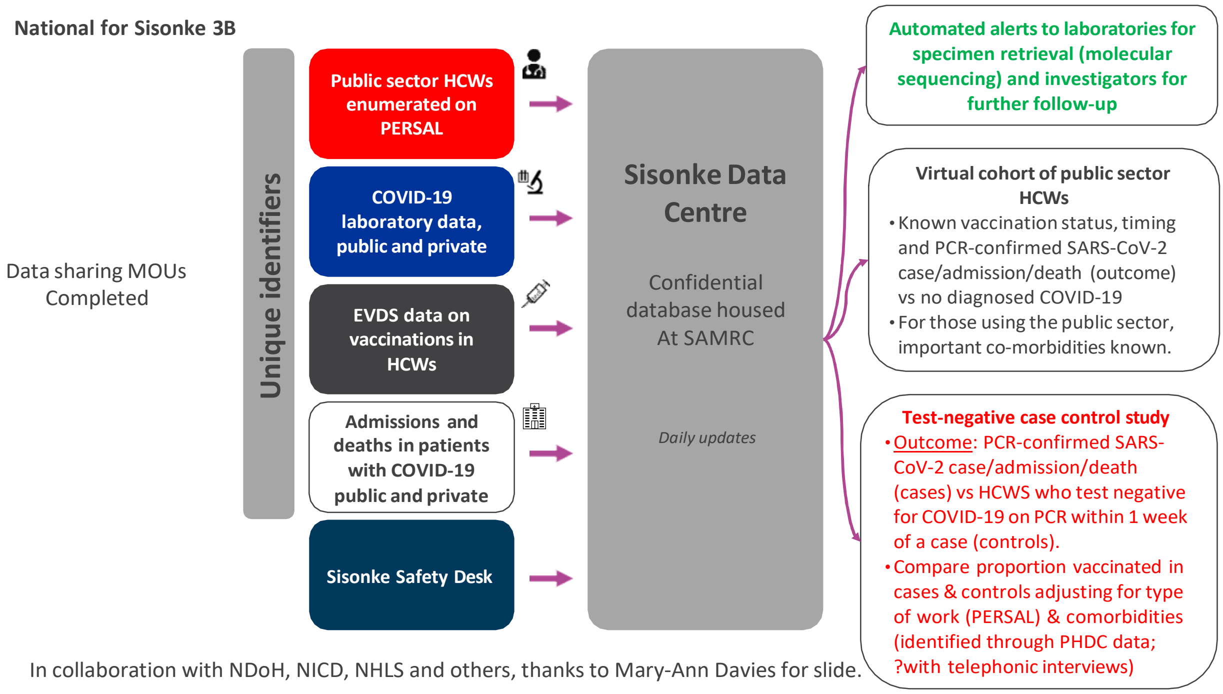

Severe allergic reactions such as anaphylaxis have been rare. The nature and extent of any immediate reactions will be recorded at sites and via the EVDS.
Participants will also be able to contact via the Sisonke desk which provides a 24/7 service or return to the site if any adverse reactions occur later.

Participants who have been flagged as high risk will also be followed up carefully for up to 28 days post vaccination through approximately weekly contact, information and the provision of an emergency number.

All pregnant HCW will be reported to Sisonke safety desk. Pregnant HCW will be followed up telephonically for adverse events and birth outcomes. Breastfeeding HCW will be followd up telephonically for adverse events in mother and baby. Additionally, breastfeeding HCW will also be asked for provide breastmilk samples at weeks 0, 6 and month 6 if still breastfeeding.

Amendments to patient facing material regarding new safety data on VITT:

All participants will be made aware of the rare risk of VITT on numerous occasions including in the ICF and PIS (see below for amendments), at the time of enrolment and vaccination, and will be asked to monitor for symptoms as described above 4-20 days post vaccination. Weekly SMSs will occur weekly up to 3 weeks to remind all vaccinees of the Sisonke desk number and the online event reporting system.

Participants who are already on the EVDS system will receive an SMS to link them to the Dear Participants letter. Those who have not yet consented will receive an amended ICF and PIS. Widespread education about this medical condition will be undertaken.

Safety Reporting:
To ensure alignment and comparability of safety data, the Brighton Collaboration and Safety Platform for Emergency vaccines (SPEAC) Project definitions for adverse events of special interest (AESI) have been applied.

8.1 Overview of safety monitoring

AE data flows through to the study database via five sources (Figure 1):

i) Electronic AE reporting CRF: After vaccination every vaccinee receives a text message with the AE reporting link. The link takes the vaccinee to an electronic reporting form that mirrors the paper based AEFI form. They are invited to complete this if they have AEs to report. About 95% of the total reports received are via this data stream. One of the text messages also includes messaging on common signs and symptoms of reactogenicity. This message will be repeated at week 2 and week 3 post vaccination to remind participants to notify the Sisonke desk of adverse events.

ii) Paper-based AE reports: AE information is captured via the standard AEFI reporting forms. This is predominantly for AEs reported at the facility/vaccination centre. These forms are emailed/scanned to the AEFI programme and / or the Sisonke safety desk email alias (which is included on the forms). The AEFI programme also forwards forms to the Sisonke safety team. These are then captured in the AE database. To date, just above 100 forms have been reported through this channel. These events are captured either by the CRS research site staff linked to the vaccine centre or the vaccine centre staff.

iii) Safety Desk Telephonic Contact: Contact details for the safety desk are available on the vaccination cards and are also included in the SMS sent. Vaccinees are reminded of this facility after their vaccination and are again invited to call in to the safety desk if they have a reportable event/ side effect. There they are attended to by pharmacovigilance nurses who capture AEs in the database; respond to generic safety concerns and advise management and/or escalate specific queries to study safety physicians.

iv) Spontaneous Case Reports: Unsolicited communication by healthcare professionals or similar. The cases will be followed up and data captured on the AE database.

v) Linkage with National Disease Databases: linkage of Sisonke study data with the NHLS Corporate Datawarehouse / COVID-19 PCR line listing, DATCOV (COVID-19 related hospitalizations) list and MRC Burden of Disease Unit/Home Affairs death registration list. This aims to identify vaccinees with the study disease outcomes, namely SARS CoV-2 PCR positivity, COVID-19 related hospitalisation and COVID-related deaths in near real-time. Linkage will run daily.

Strengthen Monitoring and Surveillance

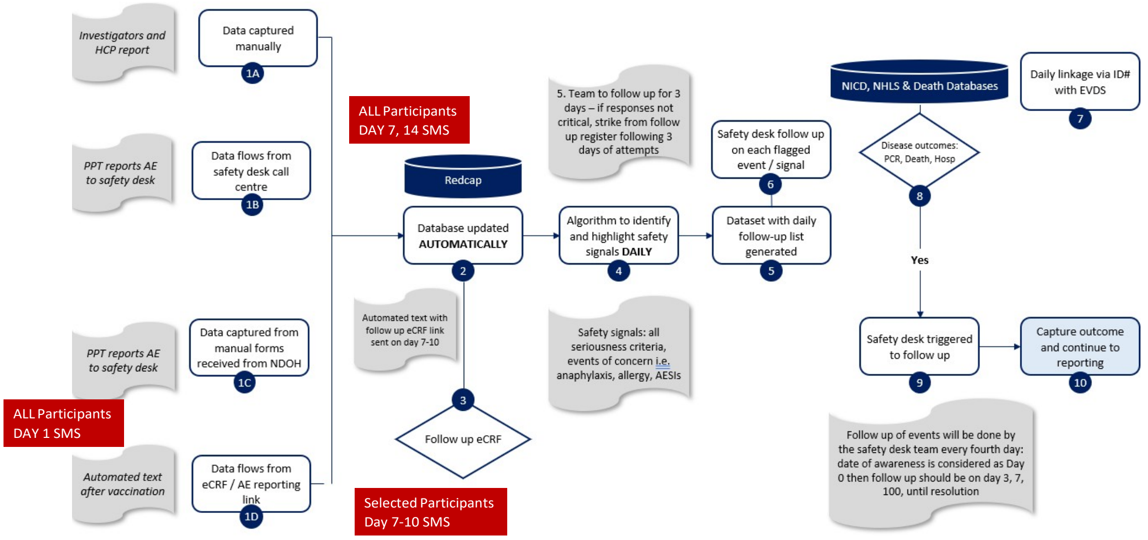

Figure 1 Overview of Sisonke pharmacovigilance and adverse event *reporting process*

Adverse events in the database are reviewed daily and mined for potential safety signals: hospitalisations, potentially life-threatening events, and events of medical concern (including pre-defined adverse events of special interest). These are flagged and depending on the nature of the event – pharmacovigilance nurses or the safety physicians follow a prescribed schedule to follow up these participants.

8.2 Follow-up: Seven to 10 days post vaccination, every participant who reported an AE receives a follow-up text which takes them to a short AE follow-up electronic form/questionnaire. Participants with ongoing symptoms and those whose conditions are worsening are also followed by the clinical safety staff.

8.3 Roles and responsibilities in safety monitoring

Safety Monitoring Committee

The safety monitoring committee is a group of independent experts with cumulative experience in ethics, infectious diseases, COVID-19, vaccine clinical trials and epidemiology/biostatistics who will provide independent oversight of the study. They will convene frequently in open forum.

Members of the Safety Monitoring Committee :

Chris Beyrer ;USA(Epidemiology and vaccinology)
Francesca Conradie ;RSA(Infectious disease)
Sipho Dlamini ; RSA(Infectious disease and vaccinology)
Jeremy Nel ; RSA(Infectious disease)
Yunus Moosa; RSA(infectious disease

Protocol Safety Review Team

Safety monitoring and oversight will be provided by the Sisonke protocol safety review team (PSRT). The PSRT is composed of the following members:
- PI and co-PIs (five members),
- HCRISA safety physicians/medical monitors (two members)
- Immunologist and Aller ologist (1 member)
- Clinical Haematology (three members)

The members of the Sisonke PSRT are responsible for decisions related to participant safety. Other protocol team members, site investigators, external experts may also be included in PSRT meetings. The PSRT will review cumulative clinical safety data on a weekly basis.

Sisonke (HCRISA) Safety	Co-opted expert members	Investigators
Simba Takuva<br><br>Azwi Takalani	Jonny Peters (Clinical Immunologist and Aller ologist)<br>Barry Jacobson (Clinical Haematology – Head Wits)<br>Vernon J Louw (Clinical Haematology – Head UCT)<br>Jessica Opie (Clinical Haematology (NHLS)	Glenda Gray<br><br>Linda-Gail Bekker<br><br>Ameena Goga<br><br>Ian Sanne<br><br>Nigel Garrett

Clinical Safety staff
Clinical safety staff at the HCRISA safety desk comprise safety physicians/medical monitors and a team of pharmacovigilance specialists (registered nurses and/or clinical associates). The roles and responsibilities of the HCRISA safety desk in relation to safety monitoring include:
- Daily monitoring and follow-up of safety clinical concerns via a centralized telephone service (Toll-free/Please Call Me text facility)
- Monitoring of data for safety and adverse events
- Liaison between clinical research sites and the protocol PSRT / Company on clinical safety issues and provide support to the PSRT
- Notifying clinical research sites and other groups of important safety concerns
- Daily linkage with EVDS, NHLS CDW database and DATCOV via unique identifiers to track safety events
- Management of safety reporting to the PSRT, IECs, PIs and SAHPRA

Telephone numbers and email addresses for contacting the safety desk are found on the protocol webpage, the relevant SOPs will also be provided to investigators and participants.

Research sites
Research sites will ensure vaccination venues align to GCP and safety requirements. i.e. presence of on-site emergency equipment i.e. emergency trolley is required for vaccinating sites and availability of trained personal for medical emergencies.. The research sites will be required to submit any safety data to the protocol clinical safety team (via an AE CRF). The clinical safety team will liaise with site investigators to obtain medical records from health facilities.
All known incidents of COVID19 will be followed up by site research staff for subsequent "breakthrough infection" visits.

8.4 Safety reporting elements

The Collaborator and Investigator will provide safety information arising from the Study to the Company on serious adverse events (SAEs), special situations including pregnancies and product quality complaints as defined within the contract. This safety information will be documented by the Investigator and reported as described in the contract from the time a subject has signed and dated an Informed Consent Form (ICF) until 28 days after the last dose of the Janssen Product(s) Under Study. The safety physician/medical monitor and the PSRT will ensure all safety events that require notification are reported to SAHPRA and the Company according to the applicable regulatory requirements.
The data elements collected during safety monitoring will include:

- adverse events, serious adverse events (SAEs) until 28 days after the last dose of the study product
- adverse events of special interest (per Brighton Collaboration and SPEAC Project definitions)
- Pregnancies throughout the study or until 3 months after last vaccination.
    - If a subject becomes pregnant during the study, in addition to reporting the pregnancy to the Sisonke safety desk within 24 hours , a determination must be made by the investigator in alignment with the protocol inclusion/exclusion criteria and in consultation with the reference safety information. Pregnancy outcomes will be monitored telephonically by the study and safety team. Follow-up information regarding the outcome of the pregnancy and any postnatal sequelae in the infant will be required.
- All HCW will receive an sms asking them to report their breastfeeding status into the Safety desk, if have not enrolled into the sub-study as a breastfeeding HCW. The routine AE form will be used to document breastfeeding status and adverse events amongst these HCW.
- SAEs beyond 28 days after the receipt of study product shall be collected/reported if the investigator/primary care provider/clinical safety staff consider the SAE to be causally related to the use of the product.
- Disease-related events and outcomes per self-report or as identified via periodic linkage to the following databases: These include COVID-19 infection, COVID-19 related disease, hospitalisations and deaths.
    - Routine and diagnostic specimen data from the national laboratory management information system are archived in the Corporate Data Warehouse (CDW) of the NHLS and COVID-19 related hospitalisations are monitored via the Daily Hospital Surveillance system (DATCOV). The databases will be scanned daily for potential new COVID-19 infections or severe adverse events/hospitalisations and evidence of COVID-19 like disease as per HCW permission. All known incidents of COVID19 will be followed up by site research staff for subsequent "breakthrough infection" visits. This will include a blood draw for immunology and nasal swab specimens for viral genetic testing. Genetic samples will be stored at BARC and moved to CAPRISA or the SAMRC biorepository when appropriate.
    - Deaths will be ascertained via family or health facility/hospital report, active tracing and the SAMRC Burden of Disease linkage with the South African National Vital Registration Infrastructure Initiative.

8.5 Frequency of safety reporting

The PSRT will review weekly cumulative safety data. SAHPRA will be provided with regular periodic reports per regulatory requirements (now amended to 2 weekly) including safety and futility reports and regular progress reports.
Response: In addition to two weekly safety monitoring and futility reports, the decisions made by the PSRT and/or the SMC will be reported to SAHPRA within 24 hours.

9.0 STATISTICAL CONSIDERATIONS

9.1 Study design

This is an open-label, single-arm phase 3B vaccine implementation study.

9.2 Sample size

In the interim analyses of the phase 3 ENSEMBLE trial, it was shown that 468 symptomatic cases of COVID-19 were detected from 43,783 adult volunteers in the USA, Latin America and South Africa. This translates to an overall attack rate of 1.1%; approximately 0.54% and 1.59% in the intervention and placebo arm, respectively, providing a vaccine efficacy of 66% against mild to severe Covid-19. Noteworthy, these estimates were driven by the force of SARS-CoV-2 infection and will vary depending on whether there is a resurgence or not. Given that vaccine efficacy was 57% in South Africa, we assume that the incidence of symptomatic SARS CoV-2 infections in this cohort might be slightly higher than 0.5%.
Based on these assumptions, an exact binomial test with a nominal 5% two-sided significance level will have >90% power to detect the difference between the null hypothesis proportion, of 0.01 (i.e., 1.0%) and the alternative proportion, of 0.007 (i.e., 0.7%) when the sample size is 11 000. **Table 3** provides scenarios for various estimates.

Table 3: Number of participants to have 90% power to detect a primary endpoint

Incidence of symptomatic SARS CoV-2 infections in the placebo arm of ENSMBLE	1.0%	1.0%	1.0%	1.3%	1.3%	1.3%	1.5%	1.5%	1.5%
Incidence of symptomatic SARS CoV-2 infections among vaccinated HCWs	0.70%	0.60%	0.50%	0.70%	0.60%	0.50%	0.70%	0.60%	0.50%
Sample Size	11000	6000	3500	3200	2103	1525	2000	1404	1200

This trial is designed such that it can generate more safety data and also identify any unexpected (i.e., rare) adverse effects of the vaccine administration, while also providing sufficient efficacy data. Therefore, a relatively large sample size is needed to detect such rare events so that more precise estimates can be obtained, and this is very critical at this stage of the vaccine life cycle.
If we target 500 000 healthcare workers, the ability of the study to detect rare safety events is shown in **Table 4**.

Table 4: Probability of observing no events, at least 1 event, or at least 2 events, for a range of estimated true event rates (N=500 000).

True event rate (%)	0 events	1+ events	2+ events
1%	<0.001	>0.99	>0.99
0.1%	<0.001	>0.99	>0.99
0.01%	<0.001	>0.99	>0.99

0.05%	<0.001	>0.99	>0.99

These probabilities in Table 4 highlight the likelihood of the study to detect either a very low or moderate safety events. Particularly, there is a very low chance (<0.001% probability) of observing no safety events if the true event rate is 0.05% or more. Moreover, the chance of observing at least one or two events is >99% if the true event rate is 0.05% or more.

9.3 Statistical analyses

Analyses for primary endpoint(s) and some of the secondary endpoints will be performed using SAS version 9.4 (Statistical Analysis Software, North Carolina, USA) and R statistical software. All HCWs will be included in the analyses aimed at measuring vaccine uptake. However, the incidence of symptomatic SARS-CoV-2 will be assessed on a sub-cohort of HCWs who will be randomly selected for further follow-up.
All deviations to be made to the statistical considerations in this protocol will be documented in the detailed statistical analyses plan (SAP) together with a detailed analysis plan for secondary objectives.

Participant demographics and baseline clinical data
Demographic and clinical data of all participants enrolled in the study will be summarized using descriptive statistics.

Incidence of symptomatic SARS CoV-2 infections
This analysis will include HCWs from the sub-cohort at their vaccination visit. The proportion of HCWs with breakthrough infections will be reported and the confidence interval of the estimate will be calculated using the score test method. This estimate will be compared to that from the placebo arm of ENSEMBLE using one sample binomial test.

Safety data (hospitalizations and/or deaths)
The number and the proportion of hospitalized or died due to COVID-19 will be reported and where necessary these results will be stratified by province, age, gender and co-morbidity status.

10.0 DATA MANAGEMENT

All data management activities will be undertaken under the applicable regulatory frameworks. This includes the U.S Food and Drug Administration (FDA) regulations, European Medicines Agency (EMA) regulations and the SAHPRA regulations. All studies will also abide by the Research Ethics Committee regulations of participating institutions. The Data management systems at the Centre for the AIDS Programme of South Africa (CAPRISA) and the SAMRC meet FDA requirements as they are CFR Part 11 and POPIA compliant. The Data Management standard processes are aligned with the Good Clinical Data Management Processes (GCDMP).

Data will be collected on electronic questionnaires which will be developed and managed by the study team. All site study staff will be trained on the correct completion of the questionnaires. If data entered on the questionnaires are taken from an external source (e.g., laboratory reports, participant records), the source documents will be maintained in the participant's medical chart or study file at the site and will be available for review. The questionnaire will then be captured into the electronic data management system.

All data will be captured on a Web-based Electronic Data Capture System and will be accessible to the study staff and the statistician via controlled password access. The data management team will have read write-access, with audit logging controlled by passwords and access validation levels. Study staff who have access to the data on the computer systems will be trained on how to access the system.

The electronic data management system will use prescribed API (Application Programming Interface) which can be set with conditional access for all inter operable integrations and data sharing as and when required.

The system will be housed in a secure data centre with restricted access control and all relevant environmental measures to ensure the integrity of the Servers. Hourly and Daily backups are created and stored in various locations in accordance to the CAPRISA Disaster Recovery (DR) plan. Physical access to the Servers and Operating systems are maintained by Information Technology department.

10.1 Data analyses

Surveillance: Automated near real-time deterministic and probabilistic linkage algorithms will be performed to link three routine COVID-19 datasets:

i. Notifiable Medical Conditions Sentinel Surveillance (NMCSS) database of SARS-CoV-2 cases
ii. DATCOV hospital database of reported confirmed COVID-19 hospital admissions
iii. The national SARS-CoV-2 vaccine registry

Breakthrough infections, defined as a positive SARS-CoV-2 PCR or antigen test ≥7 days after completion of vaccination, and hospitalisations will be identified through linking the national vaccine registry with NMCSS and DATCOV databases, respectively. Analysis of breakthrough infection frequencies and spatial distributions will provide an early warning signal of potential challenges with vaccine storage, cold chain maintenance, and administration. Breakthrough infection rates by age, sex, and vaccine priority group will be assessed to identify potential groups requiring further investigation. Samples from the first 100 breakthrough infections in each calendar quarter together with incidence-density controls will be sequenced to assess viral evolution.

10.2 Data Sharing

All study team scientists will disseminate the trial results as broadly as possible. Data will be shared with JNJ prior to release to the public. The study data will be published consistent with normal scientific practices. Research data that document, support, and validate research findings will be made available after the main findings from the final research dataset have been accepted for publication. Such research data will be modified to prevent the disclosure of personal identifiers to remain in compliance with the Protection of Human Subjects. The research team will attend conferences periodically and present trial results to a multidisciplinary scientific community. The results from this research may also be disseminated through presentations at scientific institutions/ meetings, and/or publication in scientific journals. All publications will be uploaded to a publication repository. After sharing the results with study participants, they will be presented to communities from which participants are recruited, following Good Participatory Practice guidelines. The results will also be shared with global and local policy makers. Summary results of the trial will be made publicly available through the clinical trial registry. Any datasets used for analysis in publications can be requested by investigators via an online request to the organisation. Measures will be taken to protect identifiable information in the datasets.

11.0 HUMAN SUBJECT PROTECTION AND ETHICAL OBLIGATIONS

11.1 Regulatory and Ethical Approval

The study will be conducted in accordance with all conditions of approval by the relevant regulatory authorities and ethics committee in South Africa.

11.2 Informed consent

Electronic Informed Consent will be obtained prior to the scheduled vaccination visit. Where this

is not possible written informed consent will be obtained from each study participant.

11.3 Risks and Benefits

Risks of blood drawing

Blood drawing may cause pain and bruising and, infrequently, may cause a feeling of light-headedness or fainting. Rarely, it may cause infection at the site where blood is taken. Problems from blood drawing are generally mild and may include pain, bruising, minor swelling or bleeding at the injection site and rarely, infection, vein irritation (called phlebitis), or blood clot.

Risks of nasal swabbing

Collection of a nasal swab sample may cause a nosebleed.

Subject confidentiality

Every effort will be made to protect participant privacy and confidentiality to the extent permitted by law.

Study discontinuation

This study may be discontinued at any time if approval for the study is withdrawn by regulatory authorities and ethics committees.

11.4 Monitoring

Site monitoring is conducted by Hutchinson Centre Research Institute of South Africa (HCRISA) to ensure that the rights and well-being of trial participants are protected, that the reported trial data are accurate, complete, and verifiable, and that the conduct of the trial is in compliance with the currently approved protocol/amendment(s), Declaration of Helsinki, Guidelines for Good Practice in the Conduct of Clinical Trials in Human Participants in South Africa, and with applicable regulatory requirement(s). Refer to Sisonke Phase 3b Open Label Clinical Trial Monitoring Plan for more information about monitoring schedule, reports, virtual and onsite support.

REFERENCES

1. Investigator's Brochure: Ad26COVS1 (VAC31518), Edition 1.0. Addendum 1. Janssen Vaccines & Prevention B.V. (July 2020).
2. Investigator's Brochure: Ad26COVS1 (VAC31518), Edition 1.0. Janssen Vaccines & Prevention B.V. (June 2020).
3. Sadoff J, Le Gars M, Shukarev G, Heerwegh D, Truyers C, de Groot AM, Stoop J, Tete S, Van Damme W, Leroux-Roels I, Berghmans PJ, Kimmel M, Van Damme P, de Hoon J, Smith W, Stephenson KE, De Rosa SC, Cohen KW, McElrath MJ, Cormier E, Scheper G, Barouch DH, Hendriks J, Struyf F, Douoguih M, Van Hoof J, Schuitemaker H. Interim Results of a Phase 1-2a Trial of Ad26.COV2.S Covid-19 Vaccine. N Engl J Med. 2021 Jan 13:NEJMoa2034201. doi: 10.1056/NEJMoa2034201. Epub ahead of print. PMID: 33440088; PMCID: PMC7821985.
4. Chin J, Magoffin RL, Shearer LA, Schieble JH, Lennette EH. Field evaluation of a respiratory syncytial virus vaccine and a trivalent parainfluenza virus vaccine in a pediatric population. Am J Epidemiol. 1969;89(4);449-463.
5. Fulginiti VA, Eller JJ, Sieber OF, Joyner JW, Minamitani M, Meiklejohn G. Respiratory virus immunization. I. A field trial of two inactivated respiratory virus vaccines; an aqueous trivalent parainfluenza virus vaccine and an alum-precipitated respiratory syncytial virus vaccine. Am J Epidemiol. 1969:89(4);435-448.
6. Kapikian AZ, Mitchell RH, Chanock RM, Shvedoff RA, Stewart CE. An epidemiologic study of altered clinical reactivity to respiratory syncytial (RS) virus infection in children previously vaccinated with an inactivated RS virus vaccine. Am J Epidemiol. 1969:89;405-421.
7. Kim HW, Canchola JG, Brandt CD, et al. Respiratory syncytial virus disease in infants despite prior administration of antigenic inactivated vaccine. Am J Epidemiol. 1969:89(4);422-434.
8. Moghaddam A, Olszewska W, Wang B, et al. A potential molecular mechanism for hypersensitivity caused by formalin-inactivated vaccines. Nat Med. 2006:12(8);905-907.
9. Anywaine Z, Whitworth H, Kaleebu P, et al. Safety and Immunogenicity of a 2-Dose Heterologous Vaccination Regimen With Ad26.ZEBOV and MVA-BN-Filo Ebola Vaccines: 12-Month Data From a Phase 1 Randomized Clinical Trial in Uganda and Tanzania. J Infect Dis. 2019;220(1):46-56.
10. Barouch DH, Liu J, Peter L, et al. Characterization of humoral and cellular immune responses elicited by a recombinant adenovirus serotype 26 HIV-1 Env vaccine in healthy adults (IPCAVD 001). J Infect Dis. 2013:207(2);248-256.
11. Barouch DH, Tomaka FL, Wegmann F, et al. Evaluation of a mosaic HIV-1 vaccine in a multicentre, randomised, double-blind, placebo-controlled, phase 1/2a clinical trial (APPROACH) and in rhesus monkeys (NHP 13-19). Lancet. 2018;392(10143):232-243.
12. Fox MP, Brennan A, Maskew M, MacPhail P, Sanne I. Using vital registration data to update mortality among patients lost to follow-up from ART programmes: evidence from the Themba Lethu Clinic, South Africa. TMIH. 2010;15(4):405–13.

13 Schultz N et al, Greinacher et al NEJM April 2021.

APPENDIX 1: VISIT SCHEDULE FOR TOGETHER (VAC31518COV3012) OPEN COHORT (N=500 000)

Visit Type	Screening and vaccination
Informed consent	x
Demographics and locators	x
Eligibility assessment	x
Consent to access medical and laboratory data	x
Pre-vaccination immunity (up to 100 000)	x

APPENDIX 2: Visit Schedule for Sub-cohort (N= approximately 1000-1400)

	Enrolment	Week 1, 3, 6	Month 6
Informed consent	X		
Locator and demographics	X	X	X
Symptom screen	X	X	X
Eligibility assessment	X		
Vaccination	X		
Consent to access medical and laboratory data	X		
Body temperature	X	X	X
Weight	X	X	X
Nasopharyngeal swab for SARS-CoV-2 PCR testing	X	X	X
Blood draw for SARS-CoV-2 antibody, neutralization assays and T-cell responses at week 0, 6 and month 6 and D-Dimers, FBC (Diff) Platelets (approx. 60 mls) at week 0, 1 and 3.	X	X **Antibody and T cell bloods will NOT be performed at week 1 and 3.**	X **Clotting bloods wil not be performed at month (**

APPENDIX 3: Assessment of participants with breakthrough COVID-19 infection at home or health facility

Confirmation of COVID-19 infection	X
Targeted medical history	X
Targeted physical examination including vitals	X
Pulse oximetry by site staff	X
Nasopharyngeal/nasal swab sample	X
Blood draw for SARS-CoV-2 antibody, neutralization assays and T-cell responses, Ddimers, PF4, FBC (Diff) Platelets (approx. 50mls)	X
Biomarker RNAseq blood sample (PAXgene tubes, whole blood)	X

APPENDIX 4: Sisonke (VAC31518COV3012) OPEN LABEL SCREENING FORM

**Criteria**	**Yes (tick)**	**No**
Age 18 years and older		
Health care worker in the private or public service		
Pregnant women between 16 weeks and 34 weeks gestation who have a letter from their provider or antenatal care service.		
Participants who report breastfeeding at the time of enrolment may be included.		
The President and Deputy President of South Africa *		
HCWs willing and able to comply with all scheduled visits, vaccination plan, laboratory tests, and other study procedures (for general cohort) with follow-up at an Sisonke site (for sub-cohort).		
• Capable of giving electronic or personal signed informed consent		
No significant acute or chronic medical condition, situation or circumstance that in the opinion of the PI/designee makes the participant unsuitable for participation in the study, or jeopardises the safety or rights of the participant		
**Not currently participating in any other research studies** that would interfere with the objectives of this study. The determination of whether participation in another study would be exclusionary for a given participant will be made by the PI/designee		
**No history of severe adverse reaction** associated with a vaccine and/or severe allergic reaction (e.g. anaphylaxis) to any component of the vaccine		
No significant acute or chronic medical condition, situation or circumstance that in the opinion of the PI/designee makes the participant unsuitable for participation in the study, or jeopardises the safety or rights of the participant		
Not <16 weeks pregnant at time of enrolment, and not planning conception within 3 months, and not beyond 34 weeks gestation.		
Participant has NOT experienced major venous and arterial thrombosis occurring with thrombocytopenia following vaccination with any COVID-19 vaccine should not enrol in Sisonke.		
Participants DOES NOT with a history of heparin-induced thrombocytopenia.		

Note:

- Vaccination within 14-90 days with other COVID19 or non-specific vaccines are not exclusionary but should be discussed with study PI or designee. Allow a 2-week gap between influenza vaccination and COVID-19 vaccination.

Eligible for enrolment if Yes to all questions

APPENDIX 5: INFORMED CONSENT FORM (ELECTRONIC, ONLINE)

The participant will have read the Participant Information Sheet (PIS) online and will then be asked a series of questions. They will need to answer all with TRUE or FALSE

All questions to be answered correctly in order to access final e-consent sign off.

**CONSENT FORM (SISONKE STUDY)** (VAC31518COV3012)					
Field Number	Field	Field Type	Acquisition	Logic check	Notes
Choose either true or false for each of the following statements to demonstrate that you have understood and give consent to take part in this trial. You must answer each question correctly to proceed.					
	Date	Date<br><br>DD-MON-YYYY	Automatic		Set to 'today'
	I have read and understood the participant information sheet (PIS) for the Sisonke Study either at a research/vaccination site or at **http://sisonke.samrc.ac.za** I have considered the information and any questions I have had have been answered by reading the PIS, Frequently Asked Questions (FAQs), or by contacting the study team.	Radio button<br><br>True | False	Manual	Mandatory<br><br>True	**If give wrong answer the following text will appear:** You must read and ensure you understand the participant information sheet before you can offer informed consent to this study.
	I **do** have to allow the research team conducting the study access to my medical notes for the sake of this study.	Radio button<br><br>True | False	Manual	Mandatory<br><br>False	**If give wrong answer the following text will appear:** It is important that members of the study team are allowed access to your medical records if needed, particularly if you become ill from COVID-19 or develop a side effect of treatment. This will help us understand how effective and safe the vaccine is in this situation.

	Regulatory authorities will be allowed access to my medical and study records. They will maintain confidentiality.	Radio button<br><br>True | False	Manual	Mandatory<br><br>True	**If give wrong answer the following text will appear:** It is important that regulatory authorities have access to your medical and study records. They will want to check that the study is being carried out to the highest standards.
	I will not be asked to provide any nose or throat swab or blood tests as part of this study.	Radio button<br><br>True | False	Manual	Mandatory<br><br>False	**If give wrong answer the following text will appear:** If you think you have COVID19 you will need to follow national guidance and your doctor's advice on nasal testing. You may be part of the study subset who are asked to provide a nasal/nasopharyngeal swab and blood test at the beginning, during (0, 1, 3 and 6 weeks and 6 months) and stored for further testing.
	I allow my personal details including my name, surname, date of birth, SA ID number or passport number to be used to obtain information on my health from my local healthcare provider, the laboratory testing my specimens, the department of health and national healthcare registries.	Radio button<br><br>True | False	Manual	Mandatory<br><br>True	**If give wrong answer the following text will appear:** If you want to take part in this study, it is important that these authorities are allowed to access your personal medical records so we can check drug safety and effectiveness.
	If I am admitted to hospital while taking part in the study, I give	Radio button	Manual	Mandatory	**If give wrong answer the following text will**

	permission for the research team to contact my care providers at that hospital to obtain details relevant to the study, whether that hospital is taking part in the research study or not. I consent to the disclosure of relevant information and medical records by staff at that hospital.	True | False		True	**appear:** If you want to take part in this study, it is important that these authorities are allowed to access your personal medical records so we can check drug safety and effectiveness.
	I understand that the information collected about me will be used to support other research in the future and may be shared with other researchers both within and outside South Africa. I understand the information shared will not include my name or contact details. This research would be approved separately. I understand that the results of these projects are unlikely to have any implications for me personally.	Radio button<br><br>True | False	Manual	Mandator y<br><br>True	**If give wrong answer the following text will appear:** It is important for researchers to learn as much as possible about COVID-19. All the information and any samples that we collect from you in this study may be able to help so it is important that you allow us to share the information and samples with other researchers. All research studies will be ethically approved. We will keep all information about you safe and secure. All other researchers must follow our rules about keeping your information safe.
	I will not be free to withdraw from the trial at any time, without giving any reason and without my medical care or legal rights being affected.	Radio button<br><br>True | False	Manual	Mandator y<br><br>False	**If give wrong answer the following text will appear:** You will be free to withdraw from the trial at any time, without giving any reason and without your medical care or legal rights being affected.
	If I choose to withdraw, I understand that	Radio button	Manual	Mandator y	**If give wrong answer the**

	information already acquired, along with follow-up data from other sources, may still be used for research.	True | False		True	**following text will appear:** If you take part in this program, all the information you give us before you withdraw will continue to be used for research.
	My GP or primary physician or friend/family/colleague may be contacted if necessary, to inform them of my status during the study or to obtain information needed for follow-up.	Radio button<br><br>True | False	Manual	Mandatory<br><br>True	**If give wrong answer the following text will appear:** It is important that we are allowed to contact your GP or primary physician or friend/family/colleague particularly if you become unwell at any time during this study.
	If I become pregnant during the study, I agree to inform the study team who will follow me up to check if I am experiencing any side effects from the vaccination and to monitor the outcome of my pregnancy.	Radio button<br><br>True | False	Manual	Mandatory<br><br>True	**If give wrong answer the following text will appear:** It is important that we are allowed to follow you up more regularly if you are pregnant to ensure that you are not experiencing any side effects from the medication, and to monitor the progress of your pregnancy.
	I agree to take part in the Sisonke study, including any necessary follow-up. I certify that all the information in the page above is correct. I understand that clicking 'Submit' will electronically sign the form and that signing this form electronically is the equivalent of signing a physical document.	Action button<br><br>Submit	Manual	Mandatory	
	Printed First Name	String	Manual	Mandatory	Encrypted in SE Masked to sponsor

	Printed Surname	String	Manual	Mandatory	Encrypted in SE<br>Masked to sponsor<br>Visible to site PI
	<signature box>	Participant signature	Manual	Mandatory	

APPENDIX 6: PATIENT INFORMATION LEAFLET (ENGLISH)

To be placed on the Medical Research Council website.

ADD Study Logo,

MASTER TEMPLATE

Participant Information Sheet

Sisonke Phase 3B open label Study (VAC31518COV3012)

Each **participant** must read and understand this document **before** consenting to join.

This is an open-label, single-arm phase 3B study to monitor the effectiveness of the single dose Ad26.COV2.S COVID-19 vaccine among health care workers in South Africa.

Sponsor: South African Medical Research Council

Francie van Zijl Drive, Parowvallei, Cape Town; 7505 Tygerberg, South Africa

We are inviting you to take part in a research study called SISONKE (Together)

South Africa is severely affected by the global COVID-19 epidemic, but currently no vaccine has been rolled out. The recent promising results of the 'ENSEMBLE' trial conducted by Janssen in South Africa, and the availability of a limited amount of vaccine doses, provide the rationale for a vaccination study of HCWs to inform the larger vaccine rollout.

- Please take time to read the following information carefully. Discuss it with friends and relatives if you wish. Take time to decide whether or not you wish to take part.
- You are free to decide whether or not to take part in this study. If you choose not to take part, this will not affect the care you get from your own doctors in any way.
- You can stop taking part in the study at any time, without giving a reason.

MASTER TEMPLATE
Participant Information Sheet

- Ask us if there is anything that is not clear or if you would like more information.
- Thank you for reading this information. If you decide to take part in the study you will be asked to consent to take part.

Important things that you need to know

- We want to find out two things:
    - the effectiveness of the vaccine to prevent severe disease and death from COVID19 in health care workers
    - The ongoing safety of this COVID vaccine in vaccinated health care workers
- **This is an open label study: that means everyone in this study will receive a single dose of the Ad26.COV2S COVID19 vaccine. There is no placebo.**
- Like all vaccines, the COVID vaccine used in this study, can have unwanted side effects. The most common side effect is that the injection site may be red, swollen and feel sore for a day or two.
- There may also be symptoms of "reactogenicity", e.g. fever, fatigue for 1-2 days.
- The study will fit into your normal schedule, so for most people, there are no hospital visits.
- In a small subset of HCWs only (approx. 1400), you will need to give a nasal or nasopharyngeal swab and a blood sample (about 10-12 teaspoons) before you start, at 6 weeks and at six months. If you are breasteeding we will also ask for approximately 10mL of breastmilk at these time points. (vaccination day, 6 weeks, 6 months).
- You will be in the study for 2 years.

Contents

1 Why are we doing this study?
2 Why am I being asked to take part?
3 What do I need to know about the vaccine used in this study?
4 What will I need to do if I take part?
5 What are the possible side-effects?
6 What are the possible benefits of taking part?
7 What are the possible disadvantages and risks of taking part?
8 More information about taking part
9 Contacts for further information

1 Why are we doing this study?

This study is carried out to see the effectiveness of Ad26 Covid Vaccine to prevent or reduce the severity of COVID-19 in healthcare workers in a "real world" setting.

What is COVID-19?

A new coronavirus pandemic is sweeping the world and is called COVID-19. This is caused by infection with a virus called SARS-CoV-2. The illness is usually mild, but it can cause a severe chest infection (pneumonia) or death in some people. If you want to know more about COVID-19, please look at the World Health Organisation (WHO) website: https://www.who.int/emergencies/diseases/novel-coronavirus-2019/

How is COVID-19 usually prevented?

Although some drugs reduce the severity of COVID-19 in hospital patients, vaccines are just being licensed in some parts of the world and this is the wider scale availability of vaccines in South Africa. Physical distancing, quarantine and infection control measures are the only interventions currently available here in South Africa.

What are we trying to find out?

We want to find out three things:

- Whether healthcare workers who do get infected experience a milder disease if they have received the Ad26 vaccine.
- Whether we see less COVID infections in health care workers who have been vaccinated.
- Ongoing safety of this vaccine

2 Why am I being asked to take part?

You are being asked to take part in the Sisonke study because you are a healthcare worker who may be at high risk of being exposed to COVID-19 and may therefore catch the disease.

Do I have to take part?

No, joining the study is voluntary; it is up to you to decide whether or not to take part. If you decide to take part, you can ask for this information sheet or read it on the web site and you will be asked to consent to the study. A decision to not take part will not be held against you and will not affect the standard of care you receive if you become ill at any time.

3 What do I need to know about the vaccine used in this study?

The Jansen COVID-19 vaccine, Ad26.COV2.S, is being administered under study conditions while the regulatory processes are underway in South Africa. This study has been approved by the regulatory authorities. A single-dose regimen of this vaccine has been shown to be 64% effective overall in South Africa and 85% effective overall in preventing severe disease by Severe Acute Respiratory Syndrome Coronavirus-2 (SARS-CoV-2). This vaccine also demonstrated complete protection against COVID-19-related hospitalisation and deaths as from 28 days after receiving the vaccine.

The vaccine, although protective against severe disease and hospitalisation in all regions of the world, was found to have less impact on other milder forms of disease because of new circulating virus variants, such as 501Y.V2 in South Africa. Because of this, we will be following you up to evaluate this further.

The Ad26.COV2.S study vaccine is made from a type of common cold virus called Adenovirus. The adenovirus used to make this vaccine is thought to be harmless to

people because it has been weakened so it cannot replicate and cause a cold.
The Ad26.COV2.S study vaccine includes genetic material from the SARS-CoV-2 virus. When the vaccine is injected into your body, the genetic material from SARS-CoV-2 gets “translated” to produce so called ‘spike proteins’ which are small bits of protein specific to SARS-CoV-2. Our bodies then make an immune response against these spike proteins. This immune response is our body’s way of fighting the infection. You cannot contract COVID-19 from the vaccine.
J&J is filing for the emergency use of the single dose of the Ad26COV2.S vaccine in various regions of the world, including South Africa. Emergency use approval has now been granted by FDA (USA), the European Authorities and the WHO. This study is being conducted while these processes are ongoing in attempt to offer this as an emergency to health care workers. It will be available once registered later in this year.

4 What will I need to do if I take part?

This is an open-label single arm study. That means that you will receive a single shot of the Ad26 vaccine. There is no placebo. You must be aged 18 years or older, have registered on the national vaccine site, and be willing to attend one of the vaccination centres or research sites to be vaccinated. We would also require your permission to collect hospital and laboratory information from databases.

Can I definitely take part?

Not everyone will be able to take part in this program. We need you to answer some questions first.
You need to be over 18 years and a health care worker.
Women who are pregnant, will be asked to consult their antenatal care provider and bring a recommendation for vaccination. Pregnant women must be between 16 and 34 weeks of their pregnancy in order to enrol.
We recommend that those who intend to get pregnant within three months of entering the trial or those in their 1st trimester or between 34 weeks and delivery defer their vaccination.
Lactating women are eligible to enrol.

There are some previous medical conditions which if you have we would like you to call the number below and inform the vaccine centre where you attend prior to vaccination.
If you have had a very severe reaction to a vaccination before,
You have had a severe clotting disorder for example:

- cerebral venous sinus thrombosis, (clotting in the brain)
- antiphospholipid syndrome (unusual blood disorders)
- you are on chronic anticoagulation medication, e.g. warfarin.

You may **not** participate if you have a history of thrombocytopenic thrombosis (clotting) with a previous COVID19 vaccine or have had heparin-induced thrombocytopenia.
.
If you have a recent infection or are unwell, you may be asked to wait to be vaccinated.
You will be asked to inform the research staff:

- If you have received another vaccination in the previous 30 days. This may not be a contraindication but it is important to let us know. We recommend a 14-28 day window between vaccinations.

You will also need to agree to allow access to your healthcare records to 1) confirm medical data if necessary, or 2) to contact us on the number below if you are admitted to hospital during the study or become ill so we can collect study follow up information.

What if I am immunocompromised or living with HIV?

- If you are uncertain of your HIV status or have not had an HIV test within the past 12 months we would encourage you to test for HIV before joining this study. While Ad26 vaccine is safe for people living with HIV who are not severely immunocompromised, it is important that you know your status prior to joining this trial. If you need information

about where you can get tested for HIV, we will provide you with this information. If you know you live with HIV and are on treatment, please let us know. This is not a contraindication to being vaccinated.

What if I have allergies?

You could have an allergic reaction to a vaccine, including a rash, hives, or difficulty breathing. Some allergic reactions can be life-threatening. The study staff will watch you for at least 15-30 minutes after each injection.

Always tell the study staff if you have ever had a bad reaction to any injection or vaccine. They may give you medicines in the clinic to treat serious allergic reactions. If you think you're having a severe allergic reaction after you leave the study site, contact the emergency number on the vaccination card (given below) and get medical help right away.

What if the questions show I can take part?

Before you can enter the study, you will need to complete a questionnaire about your health to make sure that this study is suitable for you.

If your answers to the questions show you can take part and you agree to join the Sisonke Study, we will ask you to sign an online consent form or a paper consent at a vaccination site. You will not be able to join the study without a signed consent.

What will happen to me during the study?

Once your suitability in the study is confirmed by the local investigator, and you have signed the eConsent/consent you will be given the vaccination. Data will be collected using routine data sources form the National Department of Health by linking your ID/passport national number, name and other locator information for up to 2 years after receiving the vaccination. If you develop COVID-19 and want to take part in a treatment study, you can do it, but we would like you to inform the study team to ensure your safety and best possible care. We may check your medical and laboratory records if we are alerted to possible breakthrough COVID infection. We ask that if you need routine care, you also mention to your provider that you have been on the Sisonke Study.

In a subset of the healthcare worker population enrolled (about 100 000 volunteers) we will also request a blood test (1 teaspoon = 5ml) at the start to check prior exposure to SARsCOV2 infection, i.e. prior immunity. This is voluntary.

In another subset/sub-study of volunteer HCWs (approx. 1000 -1400) we will draw a sample of blood (about 10 teaspoons = 60ml) to check for prior COVID19 exposure at the time of vaccination and at 1 and 6 weeks and 6 months. At that time, we will check on your health status and repeat the blood test to rule out COVID-19 infection at this time, and again at 6 months. At the end of the study we may check your medical and laboratory records. We may also conduct nasal swabs for COVID19 if indicated.

What checks and tests will be done?

It is particularly important that if you have a previous history of cerebral venous sinus thrombosis, antiphospholipid syndrome, are on chronic anticoagulation therapy or have severe allergy following vaccination we would like you to call the number below or ask to speak with the research team associated with your vaccine centre. This is because we would like to have experts consult on whether it is safe for you to be vaccinated and to advise best procedures to do so. You will also be carefully followed up, should you be vaccinated.

-This is for your own safety-

During the study, if you develop symptoms of COVID19, including cough, shortness of breath or difficulty breathing, fever, chills, muscle pains, sore throat, or new loss of taste or smell we advise you to follow the NICD guidance and seek care/ advice from your health care provider.

We will be analysing the information we receive regularly throughout the study. If there is clear evidence that the risks outweigh the benefits, we will stop the study early.

Will I get paid to take part?

There is no reimbursement for joining this study.

5 What are the possible side-effects?

What are the most common side-effects?

Pain, tenderness and redness at the injection site, headache, chills, joint pain, muscle pain, tiredness, generally not feeling well, nausea and fever have been seen with this vaccine. These reactions usually start within 1 to 2 days after the injection and most of the reactions get better within 1 to 3 days.

Are there serious side-effects?

It is rare for anyone to have a serious allergic reaction to a vaccine. If this does happen, it usually happens within minutes. The person who vaccinates you will be trained to deal with allergic reactions and treat them immediately. With fast treatment you will make a good recovery.
Vaccines similar to Ad26.COV2.S (that is, Ad26-based vaccines) have been given to participants in studies designed to prevent RSV (Respiratory Syncytial Virus), HIV (Human Immunodeficiency Virus), Ebola/filovirus, Zika Virus, HPV (Human Papillomavirus) and malaria. As of 04 September 2020, Ad26-based vaccines have been administered to approximately 114,000 participants in ongoing and completed studies, including more than 99,000 participants in an ongoing Ebola vaccine study in the Democratic Republic of the Congo and in an ongoing immunization campaign in Rwanda.

The Ad26.COV2.S has been studied in the test tube and in animals with no vaccine-related adverse effects observed. As of 2nd Feb 2021, a single injection of Ad26.COV2.S has been administered to at least 20,800 participants, aged 18 and older. Following administration of Ad26.COV2.S, fever, muscle aches and headache appear to be more common in younger adults and can be severe. For this reason, we recommend you take a fever reducer or pain reliever if symptoms appear after receiving the vaccination, or upon your study team's recommendation.

RARE CLOTTING DISORDER: Another very rare, possibly related serious side effect has recently come to light through international vaccination programs. This is the unusual medical situation where thrombosis (clotting) occurs in major blood vessels in the presence of a low platelet count (platelets are a part of our blood system that helps with clotting). The condition has become known as vaccine induced thrombocytopenic thrombosis or VITT.
This is exceedingly rare but cases have been documented in association with some COVID19 vaccines including the vaccine you will receive in Sisonke. Cases of the rare clotting syndrome have occurred within 4-20 days of vaccination (median 8 days).
It is important that if you develop any of the following symptoms during this period after vaccination we advise that you seek care **urgently**, advise your doctor that you have recently been vaccinated and **ask them to contact the Sisonke desk on 0800 014 956.**

Symptoms that should prompt you to seek care in this period include:

- new onset seizures; or weakness in a limb
- severe dizziness
- severe unrelenting blinding headaches with vomiting
- severe abdominal pain associated with vomiting
- blurred vision
- breathlessness
- pain in the chest or stomach
- swelling or coldness in a leg,
- after vaccination
- persistent bleeding
- multiple small bruises
- new leg pain reddish and/or purplish spots, or blood blisters under the skin.

When seeking medical care, please ask your practitioner to make immediate contact with the Sisonke desk, send of a blood test to check platelet count and avoid heparin until a diagnosis has been established.

There may be other risks associated with Ad26.COV2.S that we don't know about yet. If we learn new information about the vaccine and risks associated with it, we will tell you.

Please remember that we request you to report any side effects or serious medical conditions that occur after vaccination. The Sisonke safety desk will also send you an SMS after vaccination weekly until 3 weeks post vaccination to remind you.

If you become concerned about any side-effects, please call the number on your vaccination card and listed below as soon as possible.

Pregnancy and Breast Feeding

Animal studies have shown that Janssen's licensed Ad26-based vaccine against Ebola did not raise concerns in preclinical reproductive toxicity studies. These are studies in pregnant animals that received the vaccine, and then delivered animal babies. Therefore, ongoing studies with the Ebola vaccine allow pregnant women and women planning to become pregnant to receive that vaccine. While we gain more understanding about this we would ask you about your pregnancy status at the vaccination visit. If you are pregnant, you may be enrolled and vaccinated if

- You are 16-34 weeks pregnant.
- You bring a letter of recommendation from your antenatal care provider to establish how many weeks pregnant you are and whether they support vaccination for you
- Sign an additional consent to vaccination at site

If you suspect that you have become pregnant within 3 months of receiving the vaccine, please notify the number of your vaccination card. The Sisonke team will collect data about the wellbeing of your pregnancy and baby after birth. If you do not wish to be followed, you can withdraw your consent at any time by informing your doctor.

The vaccine is safe to use in women who are breastfeeding for both mother and baby.

If you are pregnant or breastfeeding we urge you to report how you feel post vaccination through the adverse event link that will be sent to you via sms. When you complete this form please indicate that you are pregnant or breastfeeding (as applicable) when you complete this form.

We will therefore ask you about your pregnancy status at the vaccination visit.

If you suspect that you have become pregnant within 3 months of receiving the vaccine, please notify the number of your vaccination card. The Sisonke team will collect data about the wellbeing of your pregnancy and baby after birth. If you do not wish to be followed, you can withdraw your consent at any time by informing your doctor.

6 What are the possible benefits of taking part in this study?

The information we get from this study will help us understand COVID vaccines and their effectiveness in South Africa.
You will receive access to an emergency use of this vaccine while we are awaiting regulatory approval. The single-dose Ad26.COV2.S vaccine regimen has been shown to be 64% effective overall in South Africa and 85% effective overall in preventing severe disease by Severe Acute Respiratory Syndrome Coronavirus-2 (SARS-CoV-2). This vaccine also demonstrated complete protection against COVID-19-related hospitalisation and deaths as from 28 days after receiving the vaccine.
You can choose to wait until the South African Health Products Regulatory Authority (SAHPRA) approves it for general use.

7 What are the possible disadvantages and risks of taking part?

There are no disadvantages to taking part in this study. Whilst there is a risk of study participation disclosure at the time of vaccination, all data will be anonymised before reporting.

8 More information about taking part

Who is organising and funding the study?

The study is funded by the SA MRC and National Dept of Health. The vaccines have been supplied by Johnson and Johnson.
The principal investigators (PI) are not receiving any money or other payment for asking you to be part. As local representative of the international sponsor, the SA Medical Research Council has overall responsibility for the conduct of the studying South Africa. The SAMRC is responsible for ensuring the study is carried out ethically and in the best interests of the study participants.

What are your choices about how your information is used?

You can stop being part of the study at any time, without giving a reason, but we will keep information about you that we already have. A decision to stop taking part at any time will not affect the standard of care you receive if you become ill.

If you agree to take part in the sub study, you will have the option to take part in future research using your data saved from this study. If you do, we will request and hand signed informed consent for this.

How will we use information about you?

We may need to use information from your medical records, your hospital and/or your healthcare provider/laboratory for this research study. This information will include where appropriate, your ID number, name, date of birth, postcode, contact details and healthcare information related to this study. We are not allowed to gather information about you that does not directly inform this study. People will use this information to do the research or to check our records to make sure that the research is being done properly.

People who do not need to know who you are will not be able to see your name or contact details.

We will keep all information about you safe and secure. Some of your information will be shared with researchers from other countries from across the world. This includes researchers from countries taking part in the study as well as those who might request use of your anonymous study information after the study is complete.

Once we have finished the study, we will keep some of the data so we can check the results. We will write our reports in a way that no-one can work out that you took part in the study.

What will happen to the results of the Sisonke study?

When the study is completed, we will publish a summary of the results.
We will also publish the results in a medical journal, so that other doctors can see them. You can ask the study team for a copy of any publication. Your identity and any personal details will be kept confidential. No named information about you will be published in any report of this study.

Who has reviewed the Sisonke study?

The study has been reviewed by international scientists. It has been approved by all relevant Clinical research committees and written approval has been granted by those committee.
It has been authorised by the South African Health Products Regulatory Authority (SAHPRA). These groups have been involved in the planning and preparation of the study and will not have access to your information.
The study has been structured in accordance with the Declaration of Helsinki (last updated: October 2013), which deals with the recommendations guiding doctors in biomedical research involving human participants. A copy may be obtained from the PI if you wish to review it.

What if new information becomes available during the course of the study?

Sometimes during a study, new information becomes available about the vaccines that are being studied. If this happens, the research team will tell you about it.

What happens if the Sisonke study stops early?

Very occasionally a study is stopped early. If it happens, the reasons will be explained to you.

What if something goes wrong for me?

If you become ill with COVID19 you will be referred to a COVID19 hospital for care. Please tell your treating doctor that you are enrolled in this study. Your treating doctor or you can contact the numbers below for more information.

Every care will be taken in the course of this study. However, in the unlikely event that you are injured by taking part, compensation may be available.

SAMRC insurance will provide compensation for reasonable medical expenses incurred as a result of study-related injury or illness, or death determined according to the guidelines laid down by the Association of the British Pharmaceutical Industry (ABPI Compensation Guidelines Version 2014), and Guidelines for Good Practice in the Conduct of Clinical Trials in Human Participants in South Africa.http://www.abpi.org.uk/media/1607/compensation_guidelines_2014.pdf

Please notify the investigator immediately of any complications, side effects and/or injuries during the study and the nature of the expenses to be covered.

If a research related injury occurs, you have not waived any of the legal rights which you otherwise would have as a participant in this study by signing this form.

The insurance does not cover medical treatment of other injuries or illnesses or injury caused by non-observance of the protocol.

The investigator is indemnified conditional on compliance with the protocol, SAHPRA and related research committees and is not a substitute for medical malpractice insurance.

Please note that if you have a life insurance policy you should enquire whether your insurance company requires notification of your intention to participate in a study like this. Information to date is that it should not affect any life insurance policy taken out. Nevertheless, you are strongly advised to clarify it with the company concerned.

9 Contacts for further information

If you want further information about the Sisonke study, contact:

The Sisonke Study Desk Call: 0800 014 956

More information is also available on this website.

If you have any concerns about the way you have been approached or treated during the study, please talk to the Sisonke desk at 0800 014 956.

After you have consulted your doctor or the ethics committee and if they have not provided you with the answers to your satisfaction, you should write to the South African Health Products Regulatory Authority (SAHPRA):

The Chief Executive Officer
South African Health Products Regulatory Authority (SAHPRA)
Department of Health
Private Bag X828
Pretoria, 0001
E-mail: Boitumelo.Semete@sahpra.org.za
Tel: 012 5010410

Thank you for taking the time to consider taking part in this study.

APPENDIX 7: PRINTED (in person) INFORMED CONSENT FORM TEMPLATE

Sisonke Open Label Study:
Participants Information and Consent Form

An Open-label, Single-arm Phase 3B study to Monitor the Effectiveness of the Single Dose Ad26.COV2.S COVID-19 Vaccine among Health Care Workers in South Africa

Study name: Sisonke [TOGETHER]

Study number: VAC31518COV3012

Study Sponsor: South African Medical Research Council (SAMRC)

Study Doctor (Investigator): [enter name, address and phone]

Dear Health Care Worker:

You are invited to take part in this research study.

Before you agree to take part in this research study, please read this document carefully.

Before you continue reading this document, here are a few key things for you to know:

- Joining this research study is voluntary. It is your choice to participate or not.
- Joining this study is not part of your regular health care.
- If you join, your participation in this study will last for about 24 months.
- If you join, you will be vaccinated
- You may have blood draws, and other laboratory tests if you are part of the substudies.
- You may take an unsigned copy of this form home to re-read and discuss with your doctor/s, family, and friends
- You may ask the study doctor and site staff any questions.
- You may choose to not participate in this study, in which case you will not lose access to any medical care or other benefits already available to you.
- Take your time to decide.

Thank you for taking the time to consider this study. You can read more about Sisonke at: http://sisonke.samrc.ac.za

Why is this open-label study being conducted?

The J&J COVID-19 vaccine, Ad26.COV2.S, is being administered under study conditions while the regulatory processes are underway in South Africa. A single-dose regimen of this vaccine has been shown to be 57% effective overall in South Africa and 85% effective overall

in preventing severe disease by Severe Acute Respiratory Syndrome Coronavirus-2 (SARS-CoV-2). This vaccine also demonstrated complete protection against COVID-19-related hospitalisation and deaths as from 28 days after receiving the vaccine.

Janssen Vaccine is filing for the emergency use of the single dose of the Ad26COV2.S vaccine in various regions of the world, including South Africa. This will happen within the next few months. This study is being conducted while these processes are ongoing in attempt to offer this vaccine as an emergency to health care workers. The vaccine will also be available once registered later in this year.

The vaccine, although protective against severe disease and hospitalisation in all regions of the world, was found to have less impact on other milder forms of disease because of new circulating virus variants, such as 501Y.V2 in South Africa. Because of this, we will be evaluating the effectiveness of the vaccine over the next 2 years.

You will receive a single injection of the Ad26.COV2.S vaccine as a participant in the study. There is no placebo.

General Information the COVID-19 vaccine

If you agree to participate in this open-label study you will receive a single injection of the vaccine and we will follow you up by reviewing your medical records and laboratory results for up to 2 years.

Already more than 43,000 participants around the world have participated in research to evaluate the safety and efficacy of this vaccine. A further 7 million have participated in the post licensure roll out of this vaccine.

You cannot get COVID-19 from the vaccine.

You may choose to not participate in this study, in which case you will not lose access to any other medical care or other benefits already available to you.

This study is funded by the SA MRC, the National Department of Health (NDoH) and the study sponsor is the SA MRC.

WHAT HAPPENS IN THIS OPEN LABEL STUDY?

The study is divided into 3 parts: 1) Scheduling your visits, 2) Vaccination Period, 3) Follow-Up Period.

WHO MAY PARTICIPATE?
You may participate if you are

- Age 18 and older

- Health care worker in the private or public service
- Willingness and ability to comply to study procedures.
- Capable of giving electronic or personal signed informed consent which includes compliance with the requirements in this protocol.
- Pregnant women between 16 weeks and 34 weeks gestation who have a letter from their provider or antenatal care service.

- Participants who report breastfeeding at the time of enrolment may be included.

You may NOT participate

- if you have any significant acute or chronic medical condition, situation or circumstance that in the opinion of the PI/designee makes you unsuitable for participation in the study, or jeopardises the safety or rights of the participant
- Participants who report being pregnant <16 weeks gestation at time of enrolment, planning conception within 3 months, or beyond 34 weeks gestation.
- Your current participation in any other research studies interferes with the objectives of this study. The determination of whether participation in another study would be exclusionary for a given participant will be made by the PI/designee.
- History of major venous and/or arterial thrombosis occurring with thrombocytopenia following vaccination with any COVID-19 vaccine.
- If you have previously been diagnosed with Heparin induced thrombocytopenic thrombosis.

Note well:

- Vaccination within 14-90 days with other COVID19 or non-specific vaccines are not exclusionary but should be discussed with study PI or designee.

IT IS VERY IMPORTANT THAT IF YOU HAVE A PREVIOUS HISTORY OF SEVERE ALLERGY TO VACCINES OR HAVE A HISTORY OF SEVERE CLOTTING DISORDER THAT YOU LET THE SISONKE TEAM KNOW VIA THE NUMBER BELOW OR ASK TO LET THE RESEARCH SITE STAFF KNOW AT YOUR LOCAL VACCINE CENTRE PRIOR TO VACCINATION. THIS IS FOR YOUR OWN SAFETY.

- This includes a history of cerebral venous sinus thrombosis (clotting in the brain), heparin-induced thrombocytopenia, or antiphospholipid syndrome (unusual blood disorders) or if you are on chronic anticoagulation medication, e.g. warfarin.

Some participants will have extra tests and procedures

If you have the above conditions, your enrolment will be discussed by the Sisonke safety team. If deemed to be suitable for enrolment and vaccination, you may need to do so under special precautions with more intensive follow up post vaccination. We will communicate this with you and assist you in this process.

Subgroup for special investigations:

There will be a sub-set of participants (approx. 1000-1400) that will have extra tests and procedures. If you volunteer to be part of the sub-group being followed up at 0.1,3, 6 weeks and 6 months, we will collect a nasal swab to see if you have COVID-19 if you become symptomatic and blood samples (up to 50 ml or 10 teaspoons) at the time of vaccination, at 6 weeks and 6 months from you to evaluate your immune response to the vaccine. If you are breastfeeding a breastmilk sample (approximately 10mL) will also be taken at these time points.

We are also interested in exploring any clotting parameter abnormalities soon after vaccination. This will require a small amount of blood to be taken (10 mls) at weeks 0, 1 and 3. Thus overall, you will attend at enrolment and 1,3 and 6 weeks and then 6 months and give 60 mls of blood.

Pre-existing infections:
In a subset of 100 000 volunteer health care workers, we may obtain a sample of blood (1 teaspoon or 5ml) to check for pre-existing exposure to SARsCOV2 infection prior to vaccination. You may have had SARsCOV2 infection and been asymptomatic. This test will inform us of this.

STUDY RESPONSIBILITIES

To participate in the study, you have responsibilities.

Do

- Give correct information about your health history and health condition.
- Tell the study staff about any health problems you have.
- Report any side effects or health problems that may occur for 24 months post enrolment.

What is the Ad26.COV2.S study vaccine?
The Ad26.COV2.S study vaccine is made from a type of common cold virus called Adenovirus. The adenovirus used to make this vaccine is thought to be harmless to people because it has been weakened so it cannot replicate and cause a cold.

The Ad26.COV2.S study vaccine includes genetic material from the SARS-CoV-2 virus. When the study vaccine is injected into your body, the genetic material from SARS-CoV-2 gets "translated" to produce so called 'spike proteins' which are small bits of protein specific to SARS-CoV-2. Our bodies then make an immune response against these spike proteins. This immune response is our body's way of fighting the infection. You cannot contract COVID-19 from the study vaccine.

How is the vaccine given?
The study vaccine is given by injection. The needle is put into the muscle in your upper arm. When possible, the injection will be given in the arm you use less.

You will remain at the study site for observation for about 15 minutes after receiving the vaccine.

There are currently no registered vaccines for COVID-19 in South Africa. There may be other studies in your area testing different vaccines against COVID-19.

WHAT ARE THE POSSIBLE SIDE EFFECTS AND RISKS OF PARTICIPATING?

Potential Discomforts, Side Effects, and Risks Associated with Ad26.COV2.S
Vaccines similar to Ad26.COV2.S (that is, Ad26-based vaccines) have been given to participants in studies designed to prevent RSV (Respiratory Syncytial Virus), HIV (Human Immunodeficiency Virus), Ebola/filovirus, Zika Virus, HPV (Human Papillomavirus) and malaria. As of 04 September 2020, Ad26-based vaccines have been administered to

approximately 114,000 participants in ongoing and completed studies, including more than 99,000 participants in an ongoing Ebola vaccine study in the Democratic Republic of the Congo and in an ongoing immunization campaign in Rwanda.

Pain, tenderness and redness at the injection site, headache, chills, joint pain, muscle pain, tiredness, generally not feeling well, nausea and fever have been seen with these study vaccines. These reactions usually start within 1 to 2 days after the injection and most of the reactions get better within 1 to 3 days.

The Ad26.COV2.S has been studied in the test tube and in animals with no vaccine-related adverse effects observed. As of 2nd Feb 2021, a single injection of Ad26.COV2.S has been administered to at least 20,800 participants, aged 18 and older. Following administration of Ad26.COV2.S, fever, muscle aches and headache appear to be more common in younger adults and can be severe. For this reason, we recommend you take a fever reducer or pain reliever such as paracetamol if symptoms appear after receiving the vaccination, or upon your study doctor's recommendation.

All vaccines can cause side effects. Problems that are not expected may happen and these may be important. If you have any side effects or problems during this study, please let the research site know immediately.

Risks and possible side effects of vaccines in general

All types of vaccinations can cause:

- Stinging, itching, arm discomfort, pain, soreness, redness, hardness, bruising and swelling where you receive the injection
- Fever
- Chills
- Rash
- Itching in other areas of your body
- Aches and pains
- Muscle and joint pain
- Throwing up and nausea
- Headache
- Dizziness
- Feeling very tired
- Fainting

These side effects usually last 2 to 3 days. So far, very few of these effects have been seen with this vaccine.

Rarely, people may have more severe side effects that limit their normal activities or make them go to the doctor. This occurred rarely with this vaccine in the phase 3 trial.

Allergic reactions

You could have an allergic reaction to a vaccine, including a rash, hives, or difficulty breathing. This is very rare. Some allergic reactions can be life-threatening. The study staff will watch you for at least 15 minutes after each injection. Always tell the study staff if you have ever had a bad reaction to any injection or vaccine. They may give you medicines in the clinic to treat serious allergic reactions. If you think you're having a severe allergic reaction

after you leave the study site, contact the emergency number and get medical help right away. Let the research site know if this occurs.

IMPORTANT NEW INFORMATION: A very rare clotting side effect

Most recently a very rare side effect has been reported in the USA in 6 people out of almost 7 million who have been vaccinated with the JnJ vaccine to date. Although not certain, it is thought this condition may be linked to vaccination through an immune phenomenon whereby auto-antibodies are made post vaccination in the body against some of the parts of the blood system leading to a tendency in the blood to make large clots. This results in the rare occurrence of the onset of clot formation with the simultaneous occurrence of low platelet numbers (platelets are a component of the blood which helps with clotting). To date, in the cases that have developed this complication, the clots have mostly formed in the brain (central vein thrombosis), abdomen (splanchnic vein thrombosis), but also in other parts of the brain. In some cases there has also been bleeding resulting in some blood spots under the skin away from the injection site. This condition has now been called Vaccine Induced Thrombocytopenic Thrombosis, or VITT.

We can reassure participants who have received the JnJ vaccine as part of the Sisonke Phase 3b study in South Africa that we have not seen any cases of the formation of clots associated with low platelet counts among the almost 300 000 people who have received the vaccine as of end of Monday 12 April.

These very rare clotting events (1-4 cases in 1 million vaccinations) have occurred 4-20 days post vaccination with a median time of 8 days post vaccination. Most of the cases to date have included women under the age of 50 years with a mean age of 33 years.

If you develop any of the following symptoms, we advise that you seek care urgently, advise your doctor that you have recently been vaccinated and ask them to contact the Sisonke desk on 0800 014 956. **Symptoms that occur in this 4-28 day period should prompt you to seek care are:**

- new onset seizures; or weakness in a limb
- severe dizziness
- severe unrelenting blinding headaches with vomiting
- severe abdominal pain associated with vomiting
- blurred vision
- breathlessness
- pain in the chest or stomach
- swelling or coldness in a leg,
- after vaccination
- persistent bleeding
- multiple small bruises
- new leg pain reddish and/or purplish spots, or blood blisters under the skin

Contact your doctor, should this condition be suspected, is advised to make immediate contact with the Sisonke desk and **avoid heparin** until a diagnosis has been established. He may also urgently request a blood test to check your platelet count.

It is important to note that thromboembolic events are a common complication of COVID-19 infection. Clotting is also associated with other commonly used medications including certain types of contraception, with comorbidities such as obesity, diabetes and cardiovascular disease as well as smoking. These more typical events do not appear to be more commonly associated with this rare condition known as VITT.

Update on safety in Sisonke:
Thus far 2.2% of healthcare workers who received the JnJ vaccine as part of the Sisonke study reported side-effects or an adverse health event following vaccination. Only 134 people were referred for further evaluation at an emergency room or hospital. Most of these events have been minor, local or systemic reactions. One person experienced a severe allergic reaction that met the international diagnostic criteria for anaphylaxis but has since made a swift and complete recovery. We have noted some thromboembolic events (clotting events where the clot breaks off and travels to another part of the body to block a blood vessel) but none of these have been associated with the features described of a clinical syndrome of thrombosis in the presence of thrombocytopaenia (low platelet counts). The events reported internationally include cerebral venous sinus thrombosis with thrombocytopenia and in some cases widespread bleeding thought to be mediated through the creation of antibodies to platelet factor IV. These very rare events have also been reported following administration of other COVID-19 vaccines, eg AstraZeneca Vaccine.

There may be other risks associated with Ad26.COV2.S that we don't know about yet. If we learn new information about the study vaccine and risks associated with it, we will tell you.

You will receive an SMS at the time of vaccination which will remind you to report any unusual symptoms or side effects and will link you to the online adverse events reporting system. Remember you can call the Sisonke desk at any time. You will also receive an SMS at 2 and 3 weeks post vaccination to remind you to let us know if you become ill at any time.

Risk of testing positive for SARS-CoV-2 antibodies

By receiving the Ad26.COV2.S vaccine, your body may have an immune response to the specific coronavirus proteins that are part of the vaccine. This immune response will not affect any results of COVID-19 tests, whether taken as part of the study or outside of the study, that are obtained from a swab of your nose (or from your throat) as these tests tell you if you currently have COVID-19 virus in your body.
If you become pregnant during or after the study and have antibodies in response to the vaccine, we don't know if the antibodies can be passed to your baby. We do know that antibodies from other vaccines, like tetanus vaccine, do get passed to the baby. For most babies, antibodies passed from the mother last for about six months.

Other potential risks
Blood draws may cause pain, tenderness, bruising, bleeding, dizziness, vasovagal response, Syncope, and rarely, infection at the site where the blood is taken. Collection of a nasal swab sample may cause a nosebleed.

Benefits of Study Participation

You will receive access to an emergency use of this vaccine while we are awaiting regulatory approval. The single-dose Ad26.COV2.S vaccine regimen has been shown to be 64% effective overall in South Africa and 85% effective overall in preventing severe disease by Severe Acute Respiratory Syndrome Coronavirus-2 (SARS-CoV-2). This vaccine also demonstrated complete protection against COVID-19-related hospitalisation and deaths as from 28 days after receiving the vaccine.
You can choose to wait until the South African Health Products Regulatory Authority (SAHPRA) approves it for general use.

Participants may benefit from additional health information and clinical care.

COMMON QUESTIONS ABOUT JOINING THE STUDY

What are the costs of participating?
There are no costs to you to be in the study. The Sponsor and National Department of Health will supply the vaccine and the tests that are part of the study.

Can I change my mind about participating?
Yes. You can agree to be in the study now and change your mind at any time and for any reason. Your decision will not change any regular care that you receive from this clinic. Please talk to your study doctor before changing your mind about participation.

What if I get COVID-19 during the study?
You should contact the number below /on the vaccination card if you have COVID disease. In addition, we will monitor hospitals in RSA for vaccinees who may become ill. If you are admitted or see a doctor, please inform them that you are on the Sisonke Study. If you are one of the subset of 400-450 people who are having more intensive follow up, we will ask you at 6 weeks and 6 months what your experiences have been.
Can I take another vaccine after getting the Ad26 COVID-19 vaccine?
If you take another COVID-19 vaccine after receiving this one, please let your doctor know. We ask that you discuss with the study staff if you are considering receiving another COVID-19 vaccine. We recommend between 14 and 28 days between any vaccination depending on which it is- please discuss with your site staff or vaccinator.

What do I do if I have questions or problems?
If you have questions about this study or any problems that you think may be related to this study, contact the study staff during business hours at the Sisonke Desk on Tel:
Sisonke desk at 0800 014 956.

BIRTH CONTROL, PREGNANCY AND BREASTFEEDING DURING THE STUDY
Animal studies have shown that Janssen's licensed Ad26-based vaccine against Ebola did not raise concerns in preclinical reproductive toxicity studies. These are studies in pregnant animals that received the vaccine, and then delivered animal babies. Therefore, ongoing studies with the Ebola vaccine allow pregnant women and women planning to become pregnant to receive that vaccine. While we understand more about this we would ask you about your pregnancy status at the vaccination visit.

If you are pregnant, you may be enrolled and vaccinated if

- You are between 16-34 weeks pregnant.
- You bring a letter of recommendation from your antenatal care provider to establish how many weeks pregnant you are and whether they support vaccination for you
- Sign an additional consent to vaccination at site

If you are pregnant or breastfeeding we urge you to report how you feel post vaccination through the adverse event link that will be sent to you via sms. When you complete this form please indicate that you are pregnant or breastfeeding (as applicable) when you complete this form,

If you suspect that you have become pregnant during the study, we ask you to notify the Sisonke Desk immediately. The Sisonke desk staff will collect information about your pregnancy and the health of your baby. If you do not wish to be followed, you can withdraw your consent at any time by informing your doctor.

What if something goes wrong for me?

If you become ill with COVID19 you will be referred to a COVID19 hospital for care. Please tell your treating doctor that you are enrolled in this study. Your treating doctor or you can contact the numbers below for more information.
Every care will be taken in the course of this study. However, in the unlikely event that you are injured by taking part, compensation may be available.

SAMRC insurance will provide compensation for reasonable medical expenses incurred as a result of study-related injury or illness, or death determined according to the guidelines laid down by the Association of the British Pharmaceutical Industry (ABPI Compensation Guidelines Version 2014), and Guidelines for Good Practice in the Conduct of Clinical Trials in Human Participants in South Africa.
http://www.abpi.org.uk/media/1607/compensation_guidelines_2014.pdf
Please notify the investigator immediately of any complications, side effects and/or injuries during the study and the nature of the expenses to be covered.
If a research related injury occurs, you have not waived any of the legal rights which you otherwise would have as a participant in this study by signing this form.
The insurance does not cover medical treatment of other injuries or illnesses or injury caused by non-observance of the protocol.
The investigator is indemnified conditional on compliance with the protocol, SAHPRA and related research committees and is not a substitute for medical malpractice insurance.
Please note that if you have a life insurance policy you should enquire whether your insurance company requires notification of your intention to participate in a study like this. Information to date is that it should not affect any life insurance policy taken out. Nevertheless, you are strongly advised to clarify it with the company concerned.

REIMBURSEMENT
There is no cost or reimbursement for you to be in this study. However, you will receive access to an emergency use of the Ad26.COV2.S vaccine while we are awaiting regulatory approval.

EMERGENCY CARE AND HOSPITALISATION:
If you seek emergency care or if hospitalisation is required at any time during the study or up to 24 month/s after receiving this vaccine, please tell the treating doctor that you are/were enrolled in the Sisonke Study and that the Sisonke Safety Desk should be informed.
The number for the Sisonke Desk is: 0800 014 956

ETHICAL APPROVAL
This clinical study protocol has been submitted to the XXX and written approval has been granted by that Committee. The study has been structured in accordance with the Declaration of Helsinki (last updated: October 2013) which deals with the recommendations guiding doctors in biomedical research involving human participants. A copy may be obtained from me should you wish to review it.
If you have any additional questions about your rights as a research participant, you should contact the xxxx who are overseeing the conduct of this study at this clinical research centre. An Ethics Committee is an independent committee established to help protect the rights of research subjects.
{name of HREC chair, Address and Telephone number}

REGULATORY APPROVAL
If you have questions about this study you should first discuss them with the Sisonke Desk, the related site team or the related Ethics Committee. If you have not been provided with answers to your satisfaction, you should write to the South African Health Products Regulatory Authority (SAHPRA) who provides regulatory approval for the study at:

The Chief Executive Officer
South African Health Products Regulatory Authority
Department of Health
Private Bag X828
PRETORIA
0001
E-mail: Boitumelo.Semete@sahpra.org.za
Tel: (012) 501 0410

SAMPLES COLLECTED FOR SCIENTIFIC RESEARCH
What happens to the samples collected from me if I am in one of the smaller subsets for more in depth evaluation?
The Sponsor may use any of your samples collected during this study to

- Understand how the Ad26.COV2.S vaccine works, or why it may cause side effects
- To better understand COVID-19 disease
- To test if you may be infected with other respiratory viruses such as influenza (flu).
- Understand why people may respond differently to the study vaccine
- To better understand vaccines made from adenoviruses
- To develop tests for Ad26.COV2.S vaccine and SARS-CoV-2 infections.

To protect your privacy, your samples will be labelled with the study number and participant number. No personal identifiers are used (such as name, initials, social security number). The scientists doing the research will not know your identity.

Your samples may be sent to the Sponsor and other members of the Johnson & Johnson group of companies and to contractors working for them. Your samples may also be shared with other researchers. Your samples will not be sold or given to any other groups for their use. Researchers working with the Sponsor are not allowed to share samples with anyone who is not authorized by the Sponsor.

You will not be paid for any use of your samples or results, or for inventions made from research on them. You are providing your samples, for use by the Sponsor. The Sponsor (and research partners, where applicable) will own the use of the results, treatments, or inventions that can be made from this research.

Your collected samples will continue to be analysed as described in this form unless you specifically ask for your samples to be destroyed. This is to protect the quality of the study.

Samples Used for Future Research

Any samples remaining after they are used for the main study will be stored for future use for up to 15 years or as defined by local regulations. Testing will depend on the available technology at the time of testing. Additionally, your samples could be used for research on future COVID-19 vaccines or other respiratory viral disease vaccines.

You may opt out of future use of your samples or withdraw your consent at any time by notifying your study doctor. If you withdraw consent for future use of your samples, your samples will be destroyed after they are no longer required for the main study. This will not affect your access to the care, medicine, and equipment you would otherwise be getting. This can be done at any time and for any reason.

The Study Staff and the Sponsor will manage your personal data (information about you) in compliance with South African regulations as described in this consent form.

What personal data will the study staff collect?

If you join this study, the study staff will collect and use your personal data that may include information about your health.

- Demographic information such as your name, your study ID #, home address, e-mail address, telephone/mobile number, date of birth, and gender which will be entered into the Vaccine Register
- Contact information about your emergency contact; and caregiver, if applicable
- The name of your regular doctor and the hospital where you would likely seek care if you become seriously ill with COVID-19
- Information about your physical or mental health or condition
- Information from any forms you are asked to complete

.

How will your personal data be protected?

All information obtained during the course of this study, including hospital records, personal data and research data will be kept strictly confidential. Data that may be reported in scientific journals will not include any information that identifies you as a participant in this study.

Any information uncovered regarding your test results or state of health as a result of your participation in this study will be held in strict confidence. You will be informed of any finding of importance to your health or continued participation in this study, but this information will not be disclosed to any third party in addition to the ones mentioned above without your written permission. The only exception to this rule will be cases of communicable diseases where a legal duty of notification of the Department of Health exists. In this case, you will be informed of our intent to disclose such information to the authorised state agency.

How will Data be used by the Sponsor?
Your data is needed for the Sponsor to learn about Ad26.COV2.S, monitor its safety effectiveness. Therefore, they will be used as planned in this study as well as within related research activities in order to:

- understand how Ad26.COV2.S works in the body
- better understand COVID-19 and associated health problems
- develop diagnostic tests
- learn from past studies to plan new studies or improve scientific analysis methods
- publish research results in scientific journals or use them for educational purposes.

How will Your Coded Data be shared and transferred by the Sponsor?
The Sponsor may share Your Coded Data with its affiliates, health and regulatory authorities, ethics committees, authorized service providers and, with select investigators and scientists conducting scientific research, that is compatible with research related to this study including statistical purposes. Your Coded Data may also be shared with scientific journals so the study results can be reviewed by independent scientists and to ensure the accuracy of results. Your identity will not be revealed in any of these cases. These data will be utilised by them only in connection with carrying out their obligations relating to this clinical study. The Sponsor will protect Your Coded Data as far as the law allows and will keep and supervise the information collected about you only for as long as needed.
Sharing of your anonymized data by the Sponsor
Anonymized means your data and samples will be stripped of your participant number as well as of any other information that could identify you. The anonymized data and samples may be shared only for scientific research as allowed by law.
How long will your personal data be stored by the Sponsor?
Records containing your personal data will be retained at the study site for a period of 15 years. In addition, the Sponsor will retain Your Coded Data for time periods as allowed per applicable laws for the identified use.
What rights do you have concerning your personal data?
If you would like to review, correct, delete, or make other requests about your personal data, you should contact your study doctor at [insert contact details].
You may not be able to review some of the data until after the end of the study and a request to delete your personal data cannot be fulfilled where regulations and laws that apply to clinical research require your personal data to be retained.
You can ask your study doctor to send any questions, concerns or complaints you may have to the Sponsor.

GENERAL STUDY INFORMATION
Who do I contact for information?
If you have any questions about the study, please contact:
Sisonke desk: 0800 014 956 or the Sisonke website at: http://sisonke.samrc.ac.za

If you feel that this study has caused you any harm, please contact:
Sisonke desk: 0800 014 956

If you have any questions about your rights as a research participant, please contact the study doctor/staff or:
[Insert IRB or IEC name and phone number]

In addition, you may contact the
Sisonke Desk: 0800 014 956.

YOUR AGREEMENT TO PARTICIPATE

If you agree to join the study, please read and then sign below.

- I have read and understood this information.
- This study has been explained to me.
- All my questions about the study, the Ad26.COV2.S experimental vaccine, and possible risks and benefits have been answered to my satisfaction.
- I give permission for my personal information to be collected from national and other laboratories as well as other approved data sources and kept in the Sponsor's database and understand that any data shared and used for the study as explained in this consent form will be Coded Data (anonymized).
- I freely agree to participate in this research study as described and understand that I am free to withdraw at any time during the study.
- I understand that I will be given a signed copy of this document to keep.
- If a caregiver is required, I consent to allow my designated caregiver to provide support with my study related activities.

I have been informed that the study doctor/staff may inform my regular doctors (if any) about my participation in this study, and I agree to this. (You may still be in this study even if you do not agree to this.)

Yes ☐ No ☐ Not applicable, I have no other doctors ☐

For the SUBSET participants only: I agree to the use of my samples for future scientific research as described in section "Samples Collected for Scientific Research".

Yes ☐ No ☐

____

Printed name and surname of participant in full

____

Signature of participant Date (dd/mmm/yyyy)

For participants who are unable to read or write, a witness should complete the signature block below:

____

Printed name and surname of witness in full

____

Signature of witness Date (dd/mmm/yyyy)

____

Printed name and surname of person obtaining consent

____

Signature of person obtaining consent Date (dd/mmm/yyyy)

APPENDIX 8: INFORMED CONSENT FOR PREGNANT OR BREASTFEEDING HEALTH CARE WORKERS

PREGNANCY

The American college of Obstetricians and Gynecologists' Immunization, Infectious Disease and public Health Preparedness Expert Work Group, the USA Center for Disease Control and the WHO have recommended the JNJ vaccines for use in pregnant and lactating women. They have concluded that pregnant women and lactating women in the USA can receive the AD 26.COV2.S vaccine. Replication incompetent or replication defective virus vaccines are not contra-indicated in pregnancy. The same type of vaccine has been authorized for use in Ebola and has been studied extensively for other illnesses.
Although there is an ongoing Phase 2 study that assesses the safety and reactogenicity of the Ad26.COV2.S administered IM as part of a 2 dose schedule (28 days apart) in pregnant women in their 2nd or 3rd trimester (NCT04765384), the FDA EUA allows for its use in pregnancy. Pregnancy has been reported in 8 participants in Ensemble 1 (4 vaccines and 4 placebo). Vaccination was within 30 days of last menstrual period. Outcomes include spontaneous abortion (1 vaccine; 0 placebo); incomplete abortion (0 vaccine; 1 placebo); elective abortion (0 vaccine; 2 placebo) and ectopic pregnancy (1 vaccine; 0 placebo).
The Sisonke 3B study has been reviewed and approved by the SAHPRA and related Ethics Committees.
Pregant women will be eligible to participate in the Sisonke Study after they have consulted with their treating obstetrician or doctor, and after evaluation by the research staff for any underlying illnesses or allergies.
Animal studies have shown that Janssen's licensed Ad26-based vaccine against Ebola did not raise concerns in preclinical reproductive toxicity studies. These are studies in pregnant animals that received the vaccine, and then delivered animal babies. Therefore, ongoing studies with the Ebola vaccine allow pregnant women and women planning to become pregnant to receive that vaccine.

The Sisonke study will evaluate the number of participants with pregnancy outcomes (including live-births, live preterm birth, still born or abortion). We will also evaluate the number of participants with pregnancy related AEs. For neonates/infants born will be followed up passively through the safety desk.
If you are pregnant, you may be enrolled and vaccinated if

- You are between 16-34 weeks pregnant.
- You bring a letter of recommendation from your antenatal care provider to establish how many weeks pregnant you are and whether they support vaccination for you
- Sign an additional consent to vaccination at site

BREASTFEEDING

COVID-19 vaccines are not able to replicate in the human body, disintegrate within 2-3 days of vaccination, and do not pass into breastmilk. Breastfeeding women have been included in all trials of the JnJ vaccine thus far, with no safety concerns reported in mothers or their infants. It is considered safe to breastfeed during this study.

Ethical Approval

This study has been reviewed and approved by a committee called (add ethics committee name). If you have questions about your rights as a research participant, or problems or concerns about how you are being treated in this study, contact (add contact details for EC).

The study has been structured in accordance with the Declaration of Helsinki (last updated October 2013) which deals with the recommendations guiding doctors in biomedical research involving human participants, the Ethics in Health Research: Principles, Structures and Processes Second Edition 2015, and Guidelines for Good Practice in the Conduct of Clinical Trials in Human Participants in South Africa. We can provide you with copies of these guidelines if you wish to review them. In addition, the recent Protection of Personal Information Act (POPIA) ensures that all South African institutions conducts themselves in a responsible manner when collecting, processing, storing and sharing another entity's personal information by holding them accountable should they abuse or compromise your personal information in any way.

South African Health Products Regulatory Authority (SAHPRA)

If you have questions about this study, you should first discuss them with your doctor or the ethics committee (contact details as provided on this form). After you have consulted your doctor or the ethics committee and if they have not provided you with answers to your satisfaction, you should write to the South African Health Products Regulatory Authority (SAHPRA) at:

The Chief Executive Officer
Dr Buitumele Semete-Makokotlela
South African Health Products Regulatory Authority
Department of Health
Private Bag X828
PRETORIA
0001
Tel: 012 501 0410/13
e-mail: Boitumelo.Semete@sahpra.org.za

YOUR AGREEMENT TO PARTICIPATE

If you agree to join the study, please read and then sign below.

- I have read and understood this information.
- I am pregnant and confirm I am >16 weeks and less than 34 weeks pregnant or I am breastfeeding.
- If pregnant, I have also consulted with my antenatal care provider and have am happy that vaccination is the right decision for me. I have brought a letter to confirm my dates.
- This informed consent registers that my questions have been answered.
- I also understand that the Sisonke Safety desk staff will collect information about your pregnancy and the health of my baby.
- I freely agree to participate in this research study as described and understand that I am free to withdraw at any time during the study.
- I understand that I will be given a signed copy of this document to keep.
- I consent to allow my designated caregiver to provide support with my study related

activities.

I have been informed that the study doctor/staff may inform my regular doctors (if any) about my participation in this study, and I agree to this. (You may still be in this study even if you do not agree to this.)

Yes ☐ No ☐

____

Printed name and surname of participant in full

____

Signature of participant Date (dd/mmm/yyyy)

____

Printed name and surname of witness in full

____

Signature of witness Date (dd/mmm/yyyy)

____

Printed name and surname of person obtaining consent

____

Signature of person obtaining consent Date (dd/mmm/yyyy)

APPENDIX 8: FOLLOW UP VISIT CRF

Sisonke (VAC31518COV3012) OPEN LABEL STUDY: FOLLOW UP VISIT

CRF for follow-up visit will interface with EVDS as much as possible

Developer instructions in italics	
**Vaccinee ID number**	
**If female: Are you currently pregnant?**	[yes] [no]
**If female: Are you currently breastfeeding?**	[yes] [no]
**Since vaccination have you had a confirmed diagnosis for COVID-19? yes / no**	
**If yes, when did you have COVID-19? month/year**	

Do you have any of the following symptoms now?

Sore throat: yes / no malaise (loss of appetite, generally unwell, fatigue, physical weakness) yes / no
headaches: yes / no muscle pain: yes / no
GI symptoms: yes / no cough yes / no
Chest congestion: yes / no runny nose: yes / no
wheezing yes / no skin rash: yes / no
Chills/rigors yes / no eye irritation or discharge: yes / no
New or changing taste disorders Yes / no red or bruised feet or toes: Yes / no
Skin rash Yes / no

Do you have any new chronic illnesses since the vaccination visit? If yes, indicate below:

Hypertension [1] Yes [2] No	Chronic kidney disease/renal failure [1] Yes [2] No	Chronic Liver Disease [1] Yes [2] No	Neurological/neuromuscular Disease [1] Yes [2] N
Heart failure – on treatment [1] Yes [2] No	Cancer [1] Yes [2] No	Prior TB infection [1] Yes [2] No	HIV [1] Yes [2] No
Asthma [1] Yes [2] No	Chr. Lung Disease/ COPD [1] Yes [2] No	Arthritis [1] Yes [2] No	Diabetes Mellitis [1] Yes [2] N
Stroke [1] Yes [2] No	Heart attack [1] Yes [2] No		
Autoimmune Disease [1] Yes [2] No			

Are you currently taking any new medicines since vaccinations: If yes, indicate below

Anti-inflammatories [1] Yes [2] No	Anti-hypertensives [1] Yes [2] No
Hormonal treatment [1] Yes [2] No	Antibiotics [1] Yes [2] No
Bactrim prophylaxis [1] Yes [2] No	Aspirin / Warfarin / Heparin [1] Yes [2] No
Ivermectin [1] Yes [2] No	ARVs [1] Yes [2] No

Current clinical readings:

Patient temperature	*in degree Celsius – one decimal point (leave blank if not available)*
Current Weight of patient kg	*(rounded off to nearest whole number. Leave blank if not available)*
**Side effect profile**	

Need to add in all the side effects with yes / no	
Saturation >93% [1] Yes [2] No<br>Sats 90-<93% [1] Yes [2] No<br>Sats <90% [1] Yes [2] No<br>Heart rate ≥90 beats/minute [1] Yes [2] No<br>Heart rate ≥125 beats/minute [1] Yes [2] No<br>Fast breathing >20 breaths per minutes [1] Yes [2] No<br>Fast breathing >30 breaths/minute<br>Pneumonia [1] Yes [2] No<br>Deep vein thrombosis [1] Yes [2] No<br>Shock (defined as systolic blood pressure <90 mmHg, diastolic blood pressure <60 mmHg, or requiring vasopressors) [1] Yes [2] No<br>Respiratory failure [1] Yes [2] No<br>Admission to ICU [1] Yes [2] No<br>Death [1] Yes [2] No	
**Blood drawn [yes] [no]** if yes, barcode	
End of CRF	

APPENDIX 9: VACCINATION VISIT ENROLLMENT VISIT CRF

Sisonke (VAC31518COV3012) OPEN LABEL TRIAL: VACCINATION VISIT
CRF for enrolment visit will interface with EVDS as much as possible

Developer instructions in italics						
**Vaccinee ID number**						
**Province: choose from drop down menu**						
**Place of vaccination: choose from drop-down menu**						
**Date of vaccination:**				*dd/mm/yyyy*		
**Date of birth**				dd/mm/yyyy		
**Gender**	Male	Female			Other:	
**If female: Are you currently pregnant?**					[yes] [no] IF pregnant, triggers safety reporting	
**If female: Are you currently breastfeeding?**					[yes] [no]	
**Ethnicity**	Black African:	Indian:	Coloured:	White:	Other:	
**Current address (needed for tracing purposes)**						
**Cell phone number:**						
**Alternate contact number**						
**Facility of employment (choose from drop down menu)**						
**Have you had a confirmed diagnosis for COVID-19 before? yes / no**						
**If yes, when did you have COVID-19? month/year**						
**Do you have any of the following symptoms now?**<br>Sore throat: yes / no malaise (loss of appetite, generally unwell, fatigue, physical weakness) yes / no<br>headaches: yes / no muscle pain: yes / no<br>GI symptoms: yes / no cough yes / no<br>Chest congestion: yes / no runny nose: yes / no<br>wheezing yes / no skin rash: yes / no						

Chills/rigors yes / no eye irritation or discharge: yes / no<br>New or changing taste disorders Yes / no red or bruised feet or toes: Yes / no<br>Skin rash Yes / no			
**Do you have any of the following chronic illnesses:**			
Hypertension<br>[1] Yes [2] No	Chronic kidney disease/renal failure<br>[1] Yes [2] No	Chronic Liver Disease<br>[1] Yes [2] No	Neurological/neuromuscular Disease<br>[1] Yes [2] No
Heart failure – on treatment<br>[1] Yes [2] No	Cancer<br>[1] Yes [2] No	Prior TB infection<br>[1] Yes [2] No	HIV<br>[1] Yes [2] No
Asthma<br>[1] Yes [2] No	Chr. Lung Disease/ COPD<br>[1] Yes [2] No	Arthritis<br>[1] Yes [2] No	Diabetes Mellits<br>[1] Yes [2] No
Stroke<br>[1] Yes [2] No	Heart attack<br>[1] Yes [2] No		
Autoimmune Disease [1] Yes [2] No			
**Are you currently taking any of the following medications NOW:**			
Anti-inflammatories [1] Yes [2] No		Anti-hypertensives [1] Yes [2] No	
Hormonal treatment [1] Yes [2] No		Antibiotics [1] Yes [2] No	
Bactrim prophylaxis [1] Yes [2] No		Aspirin / Warfarin / Heparin [1] Yes [2] No	
Ivermectin [1] Yes [2] No		ARVs [1] Yes [2] No	
**Current clinical readings:**			
Patient temperature *in degree Celsius – one decimal point (leave blank if not available)*			
Current Weight of patient kg *(rounded off to nearest whole number. Leave blank if not available)*			
**Vaccinated? [] yes [] no**			
**Lot number of vaccine**		**Blood drawn [yes] [no]** if yes, barcode	
**If selected for follow-up, date of follow-up: dd/mm/yyyy**			
End of CRF			
