# Supplementary material for: Safety evaluation of the single-dose Ad26.COV2.S vaccine among healthcare workers in the Sisonke study in South Africa: A phase 3b implementation trial
Source: PLoS Med. 2022 Jun 21;19(6):e1004024. doi: 10.1371/journal.pmed.1004024 (PMC9212139; doi:10.1371/journal.pmed.1004024)
Supplement: S2 Appendix — (PDF) [file pmed.1004024.s002.pdf]

Sisonke Adverse Event Following Immunisation

Please complete the details of the event below

To report a COVID-19 infection please use this link: https://is.gd/sisonke_bti

Thank you for taking time to collect this form. Remember to enter an accurate ID or Passport number. Your information is confidential.

Section A: Vaccine Recipient Identifying Information	
1. Reporting Date	____________ (dd-mm-yyyy)
2. Identification Method	◯ South African National Identification Number<br>◯ Passport Number
2.1. South African National Identification Number	____________
2.2. Passport Number	____________
3. Name	____________
4. Surname	____________
5. Date of birth	____________ (dd-mm-yyyy)
6. Province<br>(This refers to the place of residence within 28 days post vaccination)	◯ Eastern Cape<br>◯ Free State<br>◯ Gauteng<br>◯ KwaZulu-Natal<br>◯ Limpopo<br>◯ Mpumalanga<br>◯ Northern Cape<br>◯ North West<br>◯ Western Cape<br>◯ Other
6.1. Other area	____________
7. Mobile Number	____________
8. Sex	◯ Male ◯ Female ◯ Other
8.1. Do you have child bearing potential?	◯ Yes ◯ No
8.1. Pregnant	◯ Yes ◯ No

8.1.1. Last Menstrual Period	-- (dd-mm-yyyy)
8.1.2. Estimated Delivery Date	-- (dd-mm-yyyy)
8.2. Breastfeeding	◯ Yes ◯ No
Section B: Vaccine Administered	
1. Vaccine Name	◯ Ad26.COV2.S (JnJ COVID vaccine)
2. Date of vaccination	--
3. Vaccination Centre	--
Section C: Trigger Event - Minor Reactions	
1. Date of Onset	--
2. Did symptoms started after vaccination	◯ Yes ◯ No
2.1. Did symptoms worsen after vaccination?	◯ Yes ◯ No
3. Do you have minor injection site reactions	◯ Yes ◯ No<br>(Please click "Yes" to review the options)
3.1. Minor injection site reactions	☐ Swelling < 5cm<br>☐ Redness<br>☐ Induration/hardness<br>☐ Rash<br>☐ Other
3.2. Other	--
4. Do you have minor systemic reactions	◯ Yes ◯ No<br>(Please click "Yes" to review the options)
4.1. Minor systemic reactions	☐ Mild Fever < 38 deg C<br>☐ Mild Headache<br>☐ Mild body Aches<br>☐ Mild pain (to touch/on movement, but not interfering with daily activities)<br>☐ Fainting<br>☐ Other
4.2. Other systemic reactions	--
Section D:Trigger Event: Severe Reactions	

5. Do you have a severe reaction'?	◯ Yes<br>◯ No
5.1. Reporters Name & Surname	________________
5.2. Designation	◯ Doctor<br>◯ Clinical Associate<br>◯ Nurse<br>◯ Pharmacist<br>◯ Other
(Removed) Reporter's Designation	◯ Self - report<br>◯ Health Care Professional<br>◯ Other
5.2.1. Other reporter's designation	________________
5.3 Institution & Department	________________
5.4. Telephone Number	________________
5.5 Mobile Number	________________
5.6. Email	________________
6. Do you have severe injection site reactions	◯ Yes ◯ No<br>(Please click "Yes" to review the options)
6.1. Severe injection site reactions	☐ Pain, redness and /or swelling >3 days<br>☐ Swelling > 5cm<br>☐ Swelling beyond nearest joint<br>☐ Lymhadenitis<br>☐ Abscess<br>☐ Necrosis at vaccination site<br>☐ Other
6.2. Other severe local reactions	________________
7. Do you have severe systemic reactions	◯ Yes ◯ No<br>(Please click "Yes" to review the options)

7.1. Severe systemic reactions	☐ Hospitalisations<br>☐ Fever =/> 38 deg C<br>☐ Seizures<br>☐ Toxic Shock Syndrome<br>☐ Death<br>☐ Thrombocytopaenia<br>☐ Encephalopathy<br>☐ Vomiting<br>☐ Collapse/shock - like state<br>☐ Anaphylaxis<br>☐ Sepsis<br>☐ Diarrhoea<br>☐ Other
7.2. Other severe systemic reactions	--
8. Adverse Event of Special Interest	◯ Yes ◯ No ◯ Not sure<br>(Please click "Yes" to review the options)
8.1. Please select adverse event of special interest	☐ Acute septic arthritis<br>☐ Acute cardiovascular injury<br>☐ Acute disseminated encephalomyelitis<br>☐ Acute liver injury<br>☐ Anaphylaxis<br>☐ Chilblain - like lesions<br>☐ Coagulation Disorders (thrombo - embolisn, haemorrhage)<br>☐ Enhanced disease following immunisation<br>☐ Erythema multiforme<br>☐ Generalised convulsions<br>☐ Guillian Barre Syndrome<br>☐ Meningoencephalitis<br>☐ Single organ cutaneous vasculitis<br>☐ Thrombocytopaenia<br>☐ Anosmia/ageusia<br>☐ Acute Respiratory Distress Syndrome<br>☐ Multisystem inflammatory syndrome in children
9. Is this event a serious adverse event following immunisation (AEFI)	◯ Yes ◯ No<br>(Please click "Yes" to review the options)
9.1. Select Seriousness Criteria	◯ Death<br>◯ Hospitalisation<br>◯ Disability/Incapacity<br>◯ Life Threatening<br>◯ Congenital anomaly in off - spring of vaccine recipient<br>◯ Other
9.2. Other serious criteria	--
Section E: Past Medical History	
1. Have you had a similar reaction following vaccinations and other medications	◯ Yes<br>◯ No
1.2 Name vaccination &/or medication and describe reaction	--

2. Are you taking any other medications	◯ Yes<br>◯ No
2.1 List medications and last date of administration (do not include medications taken to treat reaction)	______________________
3. Insert additional Information	______________________
Section F: What was the outcome of the case following the AEFI in Vaccinee	
1. Outcome	◯ Recovering<br>◯ Recovered (fully)<br>◯ Recovered with sequelae<br>◯ Not recovered<br>◯ Died<br>◯ Hospitalisation
1.1 Received medical management and observation in the Emergency Department	◯ Yes<br>◯ No
1.2 Admitted to ward	◯ Admitted to ward =< 24 hours<br>◯ Admitted to ward > 24 hours
1.3. Date of admission	______________________
1.4. Name of Hospital	______________________
1.5. Have you been discharged	◯ Yes<br>◯ No
1.5.1. Discharge date	______________________
2. Outcome date	______________________<br>(dd-mm-yyyy)
3. Date of death	______________________
4. Did the symptoms interfere with usual social & functional activities	◯ No<br>◯ A little bit, however I did not take treatment<br>◯ A little bit and I took treatment<br>◯ I was unable to perform my usual activities<br>◯ I needed hospitalization<br>◯ Potentially life-threatening symptoms
5. Additional comments	______________________

6. For office use only	--
Section G: Doctor review of event	
1. Is this event an SAE/AESI?	◯ Yes ◯ No
2. Age of vaccine at time of reporting event	--
3. AE Term	--
4. Final outcome	◯ On-going<br>◯ Recovering<br>◯ Recovered<br>◯ Died<br>◯ Lost to follow-up
4.1. Final outcome date	--
5. Doctors comments	--
Upload Case Record	
