# Supplementary material for: Safety evaluation of the single-dose Ad26.COV2.S vaccine among healthcare workers in the Sisonke study in South Africa: A phase 3b implementation trial
Source: PLoS Med. 2022 Jun 21;19(6):e1004024. doi: 10.1371/journal.pmed.1004024 (PMC9212139; doi:10.1371/journal.pmed.1004024)
Supplement: S3 Appendix — (PDF) [file pmed.1004024.s003.pdf]

# Statistical Analysis Plan

| | |
|---|---|
| TRIAL FULL TITLE | Open-label, single-arm phase 3B implementation study to monitor the effectiveness of the single-dose Ad26.COV2.S COVID-19 vaccine among health care workers in South Africa (VAC31518COV3012) |
| SAP VERSION | Version 1 |
| SAP VERSION DATE | 07 May 2021 |
| TRIAL STATISTICIANS | Nonhlanhla Yende-Zuma and Tarylee Reddy |
| Protocol Version (SAP associated with) | Version 3.0 |
| TRIAL PRINCIPAL INVESTIGATORS | Glenda Gray, Linda Gail Bekker, Ameena Goga, Nigel Garett, Ian Sanne, Simbarashe Takuva |
| SAP AUTHOR(s) | Nonhlanhla Yende-Zuma, Tarylee Reddy and Cheryl Cohen |

I give my approval for the attached SAP dated 07 May 2021:

| Designation | Name | Date | Signature |
|---|---|---|---|
| Statistician author | Tarylee Reddy | 27 July 2021 | [signature] |
| Protocol Statistician and co-author | Nonhlanhla Yende-Zuma | 27 July 2021 | [signature] |
| Epidemiologist, Statistician Reviewer and co-author | Cheryl Cohen | | |
| Principal Investigator | Glenda Gray | | |

**Modifications to Statistical Analysis Plan after Trial Initiation**

| Version | Date | Changes from the previous version |
|---|---|---|
| 2 | 07 May 2021 | |
| 3 | 27 July 2021 | A comparator group of unvaccinated healthcare workers, essential, first responders and frontline workers who are members of different medical aid schemes will be included in the analyses. This comparator group will enable us to produce a robust and a more generalisable vaccine effectiveness estimate. |

Table of Contents


1 Abbreviations and Definitions ..... 3
2 Introduction ..... 4
2.1 Preface ..... 4
2.2 Scope of the analyses ..... 4
3 Study Objectives and Endpoints ..... 4
3.1 Study Objectives ..... 4
3.2 Endpoints ..... 5
4 Study Methods ..... 5
4.1 General Study Design and Plan ..... 5
4.2 Study Assessments ..... 6
5 General Analysis Considerations ..... 6
5.1 Timing of Analyses ..... 6
5.2 Analysis Populations ..... 7
5.2.1 Full Analysis Population (or Intention to Treat or Modified Intention to Treat) ..... 7
5.2.2 Safety Population ..... 7
5.3 Covariates and subgroups ..... 7
5.4 Missing Data ..... 7
5.5 Derived variables ..... 8
5.6 Demographic and baseline variables ..... 9
5.7 Monitoring of adverse events ..... 9
6 Effectiveness Analyses ..... 9
6.1 Analysis of primary endpoint ..... 9
6.1.1 Retrospective matched cohort analysis ..... 10
6.1.2 Unmatched or matched test negative case-control (TNCC) ..... 11
6.2 Analysis of secondary endpoints ..... 13
7 Safety analyses ..... 13
8 Quality Assurance of Statistical Programming (As Applicable) ..... 13
9 References ..... 14
10 Listing of Tables, Listings and Figures ..... 15

# 1 Abbreviations and Definitions

| | |
|---|---|
| AE | Adverse Event |
| CRF | Case Report Form |
| IMP | Investigational Medical Product |
| SAP | Statistical Analysis Plan |
| HCW | Healthcare worker |

# 2 Introduction

## 2.1 Preface

Healthcare workers (HCW) and other essential, frontline and first responders provide essential services, particularly with regard to the COVID-19 pandemic. As frontline workers they risk daily exposure to SARS-CoV-2. Despite the extensive use of non-pharmaceutical interventions, such as personal protective equipment (PPE), HCW continue to contract SARS-CoV-2, with a number of HCW developing severe disease resulting in hospitalisation or death. In South Africa HCWs are 4-7 times more likely to get COVID-19 infection. To date more than 55 000 HCW have already tested positive for COVID-19. Even HCW who remain asymptomatic or only develop mild disease are forced to isolate – this has exacerbated staff shortages and undermined the ability of the health sector to respond to the high demand for hospital based care due to the ongoing pandemic. Other frontline workers who were unable to work from home during periods of lockdown are also at increased risk of infection from SARS-CoV-2 (e.g. teachers, police force) and have since been prioritized for vaccination from end June. The South African Government Covid-19 Vaccination Strategy prioritised the vaccination of HCW in phase 1 of the vaccine rollout, underscoring the national agreement that HCW constitutes a priority group. The proposed study will be conducted in collaboration with the National Department of Health. Implementation lessons learnt with vaccine rollout to HCW will be used to inform vaccine rollout to the public in subsequent phases.

## 2.2 Scope of the analyses

These analyses will assess the effectiveness of Ad26.COV2.S vaccine on all COVID-19 infections, especially severe COVID-19, hospitalizations and deaths in HCWs (some who were part of the Sisonke study), first responders and other essential and frontline workers in South Africa. For ease of reference, all workers will be referred to as "essential workers" in line with the Disaster Management Act's definition of essential workers.

# 3 Study Objectives and Endpoints

## 3.1 Study Objectives

*Primary*

To assess the effectiveness of Ad26.COV2.S vaccine on severe COVID, hospitalizations and deaths in HCWs , first responders and other essential and frontline workers in South Africa.

*Secondary*

- To estimate the incidence of symptomatic SARS CoV-2 infections in vaccinated HCW
- To estimate vaccine uptake among HCWs in South Africa as part of the Sisonke programme
- To monitor the genetic diversity of breakthrough SARS CoV-2 infections among HCW vaccinated as part of the Sisonke programme
- To monitor immunological responses (neutralising, non neutralising antibodies and T cell responses) in HCWs with breakthrough infections
- To measure baseline SARS CoV-2 antibody testing to evaluate pre-existing immunity in up to 100 000 HCWs

In a subgroup of participants (approx 400-450 people) including representative sub-populations of interest, e.g. elderly, immune compromised, pregnant:

- To compare serum neutralization and immune responses before and after vaccination at 6 weeks and up to 6 months.
- To monitor for asymptomatic infection

*Exploratory*
To establish a link between the national pharmacovigilance system to assist with monitoring safety and any unexpected adverse effects

## 3.2 Endpoints

*Primary*
Rates of laboratory-confirmed SARS CoV-2 related hospitalizations and deaths among vaccinated versus unvaccinated HCWs, first responders and other essential and frontline workers.

*Secondary*

- Incidence rate of laboratory-confirmed SARS CoV-2 infection on PCR or antigen test as indicated by self-report, health insurance claims and records and validation through linkage to national laboratory records.
- Rates of severe disease in vaccinated HCW who are found to be RT-PCR positive vaccination as measured by hospitalization, ICU admission and death
- Genetic diversity of breakthrough infection virus as determined by whole genome sequencing.
- Prevalence of SARS CoV-2 seropositivity at baseline
- Levels of neutralising antibodies, non neutralising antibodies and T cell immunity in the blood samples of health care workers who have breakthrough COVID-19 infection. This will be measured as soon as possible after the time of infection.
- Anti-SARS-CoV-2neutralizing antibody titres and T cell responses among vaccinees in groups of interest before and after vaccination
    - Groups of interest: approximate sub-set of 100 vaccinees aged 20-55 years; 100 vaccinees aged >55 years; 100 stable HIV positive vaccinees; 50 with comorbidities, etc)

- Rates of asymptomatic infection at baseline and follow up using SARS CoV-2 PCR and antibody testing in a subset of HCWs.
- The proportion of HCWs who registered for vaccination on the EVDS and the rate of vaccination in HCWs per week of the study

*Exploratory*

- Numbers and rates of safety events and/or unexpected adverse effects reported to the safety desk
- Monitor pregnancies and pregnancy outcomes reported to the safety desk

# 4 Study Methods

## 4.1 General Study Design and Plan

This is multi-centre open-label, single-arm phase 3B implementation study in HCW in South African individuals at least 18 years of age. This study will be conducted by TOGETHER (VAC31518COV3012)

sites in collaboration (where appropriate) with routine National Department of Health vaccination centres in South Africa. All HCWs who register on the National Vaccination Registry will be eligible for enrolment. Participants will receive appointments for vaccination using the registry. Vaccination will be overseen by trained personnel linked to ENSEMBLE trial sites with a follow-up duration of up to 2 years for surveillance. The end-of-study will be considered as at least 6 months and up to 2 years of follow up for the last participant enrolled in the study. Participants will receive IM injection of Ad26.COV2.S at enrolment at a dose level of $5\times10^{10}$ vp. Surveillance for vaccine effectiveness (VE) may continue for up to 2 years post vaccination.

## 4.2 Study Assessments

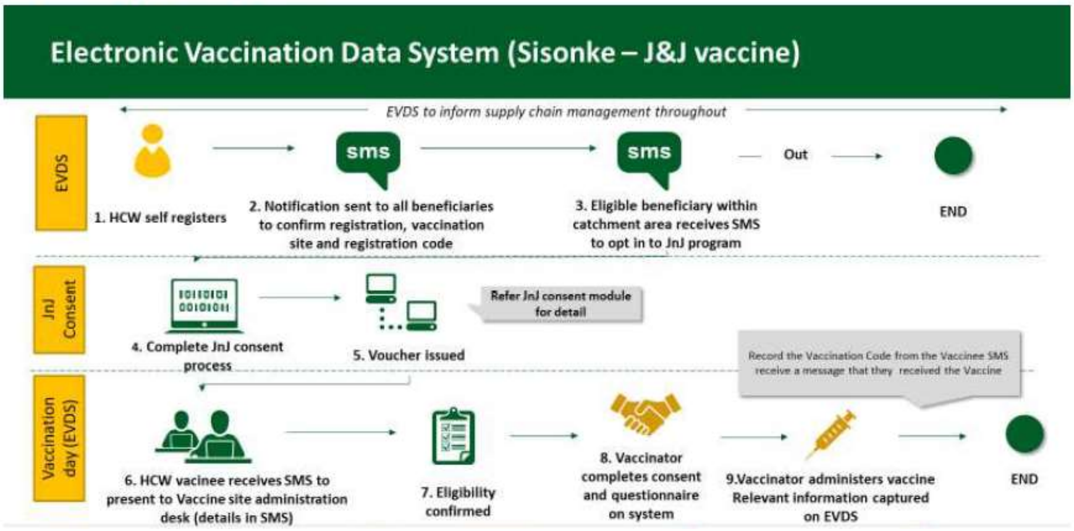


At outset the plan was to compare outcomes for HCWs first vaccinated as part of Sisonke (around 500K) with the balance of HCW estimated in the country (750K). This has not been possible because 1) the estimate of 1.25K appears to have overestimated the number of HCWs in the country; 2) almost all patient-facing HCW were vaccinated as part of Sisonke; and 3) unvaccinated health workers were quickly vaccinated after Sisonke as part of Phase 1B and so cannot contribute follow-up time, importantly during the third wave, to the analysis. Therefore, a comparator group of unvaccinated essential workers who are members of different medical aid schemes, and for whom importantly test results (both positive and negative, the latter being a pre-requisite for a test-negative case control analysis) and COVID hospitalisations are available in South Africa will be included in the analyses.

# 5 General Analysis Considerations

## 5.1 Timing of Analyses

- *The final analysis will be performed either (i) after the last HCW to be vaccinated under the*

*Sisonke programme has contributed a minimum of two months in the study, or (ii) when the PI(s) decide to stop the study.*

## 5.2 Analysis Populations

This section is designed to identify the characteristics needed for inclusion in particular populations used in the analyses.

### 5.2.1 Full Analysis Population (or Intention to Treat or Modified Intention to Treat)

- All HCWs in the Sisonke study who could be matched to any unvaccinated matched pair across different medical aid schemes
- Vaccinated and unvaccinated HCWs , first responders and other essential and frontline workers who are members of contributing medical aid schemes, who could be matched to create a 1:1 or 1:3 vaccinated to unvaccinated pair depending on the availability of unvaccinated individuals.
- Vaccinated and unvaccinated HCWs , first responders and other essential and frontline workers who are members of contributing medical aid schemes, who could be matched to create a 1:1 or 1:3 case to control pair depending on the availability of controls (i.e., individuals without the disease of interest).

### 5.2.2 Safety Population

- All vaccinated HCWs who participated in the Sisonke study. Safety data will be analysed for this population and no VE estimate will be calculated

## 5.3 Covariates and subgroups

The following covariates are expected to influence the primary and some of the secondary endpoints:
Age, gender, health status (comorbidities), geographical location, exposure risk and calendar time of vaccination and follow up since the incidence of COVID-19 varies over time and HCWs working in COVID-19 wards were prioritized in the Sisonke study and this might introduce bias in the overall analyses.

An exploratory subgroup analysis will be carried out provided the number of events:

- Analyses stratified by age group ( >55 years vs. ≤ 55 years)
- Analyses stratified by health status (this will be specific to each comorbidity- HIV, diabetes, hypertension, etc . If data on comorbidities is too sparse, it will be aggregated to form a binary variable - yes vs. no)
- Analyses stratified by calendar time of vaccination and follow up
- Analyses stratified by worker exposure risk (as reflected by number of pre-existing COVID tests and proportion with a prior infection).

## 5.4 Missing Data

For analyses of primary or secondary endpoints with missing data, multiple imputed data analysis will be performed using Multivariate Imputation by Chained Equations; complete data analysis will

be performed as a sensitivity analysis to check for inconsistencies. The percentage of participants followed for each outcome data parameter will be reported for all predefined outcomes (primary and all secondary). Exploratory outcome analyses will be planned by the study group on suggestions from the reviewers/editors etc. Percentage with followed/
missing data will also be reported for these outcomes.

## 5.5 Derived variables

Outcomes will be defined as per FDA guidelines (accessible at https://www.fda.gov/media/137926/download). For the purpose of analysing medical scheme data severe COVID-19 infections will be defined as evidence of a hospital admission for COVID, and for critical COVID evidence of critical care or intensive care admission.

Mild COVID-19

- Positive testing by standard RT-PCR assay or an equivalent testing including antigens in HCW with symptoms
- Symptoms of mild illness with COVID-19 that could include fever, cough, sore throat, malaise, headache, muscle pain, nausea, vomiting, diarrhea, and loss of taste or smell, without shortness of breath or dyspnea
- No clinical signs indicative of moderate, severe, or critical severity

Severe COVID-19

- Positive testing by standard RT-PCR assay or an equivalent test
- Symptoms suggestive of severe systemic illness with COVID-19, which could include any symptom of moderate illness or shortness of breath at rest, or respiratory distress
- Clinical signs indicative of severe systemic illness with COVID-19, such as respiratory rate ≥ 30 per minute, heart rate ≥ 125 per minute, SpO2 ≤ 93% on room air at sea level or PaO2/FiO2 < 300
- No criteria for Critical Severity

Critical COVID-19

- Positive testing by standard RT-PCR assay or equivalent test
- Evidence of critical illness, defined by at least one of the following:
    - Respiratory failure defined based on resource utilization requiring at least one of the following:
        - Endotracheal intubation and mechanical ventilation, oxygen delivered by high flow nasal cannula (heated, humidified, oxygen delivered via reinforced nasal cannula at flow rates > 20 L/min with fraction of delivered oxygen ≥ 0.5), non-invasive positive pressure ventilation, ECMO, or clinical diagnosis of respiratory failure (i.e., clinical need for one of the preceding therapies, but preceding therapies not able to be administered in setting of resource limitation)
    - Shock (defined by systolic blood pressure < 90 mm Hg, or diastolic blood pressure < 60 mm Hg or requiring vasopressors)
    - Multi-organ dysfunction/failure

## 5.6 Demographic and baseline variables

Baseline demographics and clinical data will be summarised using descriptive statistics; normally distributed data by the mean and standard deviation (SD) and skewed distributions by the median and interquartile range (IQR). Binary and categorical variables will be presented using counts and percentages.

## 5.7 Monitoring of adverse events

Safety events/unexpected adverse events will be monitored passively through several systems:

- A vaccination card will be provided to participants, with a telephone number. Participants will be asked to call the number if they experience any adverse events
- The EVDS and other systems will be used to track safety events / adverse events, through a linked pharmacovigilance system (see Clinical Safety section of the protocol)
- A patient alert card will be provided to participants separately or as part of the vaccination card, with a helpline number on the card for reporting safety /adverse events

# 6 Effectiveness Analyses

## 6.1 Analysis of primary endpoint

World Health Organization provided a guide on different study design that can be used to calculate vaccine effectiveness in the context of observational studies. Compared to randomized controlled trials, most observational studies have weaknesses due to the fact that vaccinated and unvaccinated people potentially differ in key characteristics such as risk of infection, access to testing and health seeking behaviour. Therefore, measures should be taken to minimize differences either among vaccinated and unvaccinated people or among cases and controls.

Two of the studies that are easy to implement and have been used in other countries (England, Scotland, Israel, USA, Qatar) are matched cohort study and test-negative case control study (TNCC). Although not without biases, WHO recommends TNCC design as an efficient an accurate method to be used in low and middle income countries to assess VE against severe and symptomatic COVID-19.

*Primary endpoint:*
Rates of hospitalizations and deaths among vaccinated versus unvaccinated workers
Population: Full analysis population

### 6.1.1 Retrospective matched cohort analysis

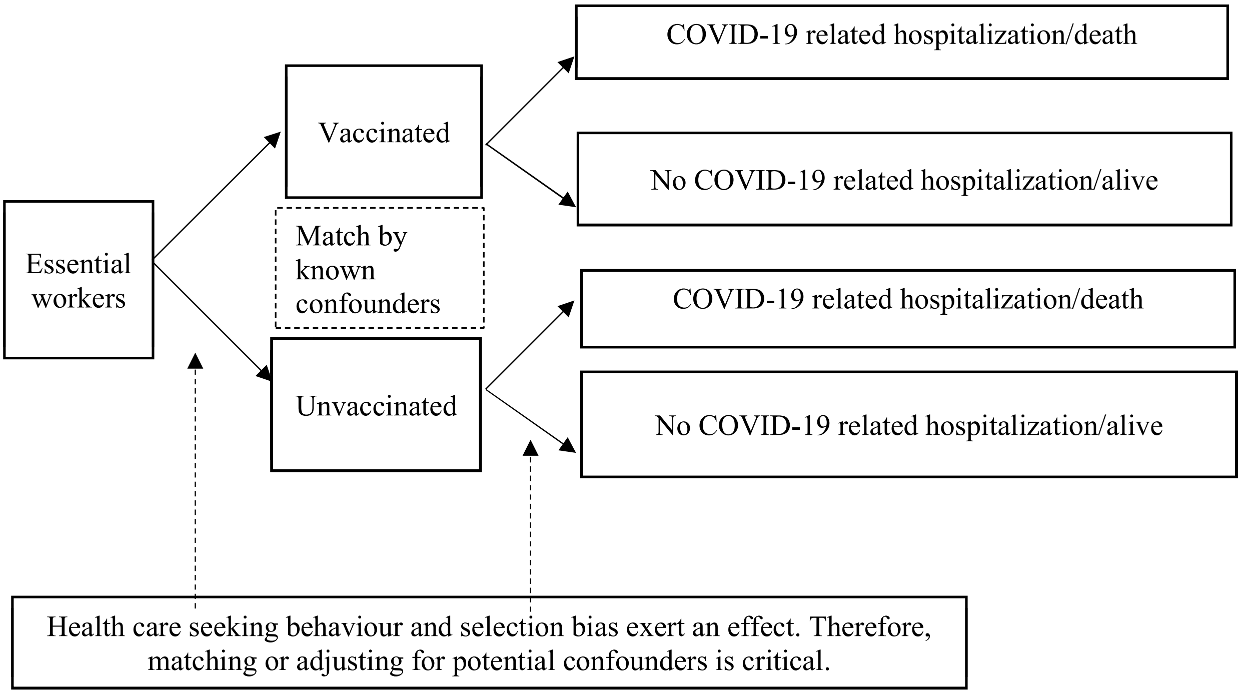


Figure 1: Flow diagram showing the matched cohort study design.

Vaccinated and unvaccinated individuals were **matched** on factors that were expected to confound the effect of vaccine on each outcome. Those factors were age, gender, , geographic location and number of comorbidities. Exposure risk will be managed depending on the number of unvaccinated members available for analysis. Matching will be performed until a sample provides balance in the number of prior COVID-19 tests and proportion with previous documented infection between groups. If unachievable, previous exposure will be used as a matching criterion.
A person can contribute both unvaccinated and vaccinated person-time, if vaccinated during the course of follow-up. Data for vaccinated and unvaccinated matched pair will be censored when the unvaccinated individual eventually receives vaccine.

In this matched cohort design, a range of different outcomes (including SARs-CoV-2 PCR confirmed infection) will be used in the analyses. It will not be limited to COVID-19 related hospitalisations or death.

Importantly, vaccinated people often differ in key characteristics from unvaccinated people, leading to biases, and these patterns are likely to change over time during the course of a prioritized vaccine rollout. This bias can or should be addressed by matching and also calculating the VE for negative control outcomes (e.g. time period or for a disease when no effectiveness expected) .

A Poisson regression model with a robust variance will be used to estimate the effectiveness of the vaccine against hospitalisations or death, where VE is defined as (1-risk ratio)*100. The 95%

confidence intervals will be calculated using the percentile bootstrap method with 500 repetitions where the matching process forms each iteration. The same principle will be applied to the symptomatic SARS-CoV-2 infection outcome.

Given that it takes approximately 14 days post vaccination to build immunity, VE will be assessed at the following intervals: 0-13, 14-20, 21-27, >27 days post vaccination.

The censoring time will be defined as 17 July 2021. The aforementioned analysis will be conducted separately for the following subgroups (provided there will be enough events):

1) Younger vs older individuals
2) Health status (comorbidities vs. no comorbidities)
3) Analyses stratified by calendar time

Descriptive statistics to assess the distribution of covariates among vaccinated and unvaccinated individuals will be conducted.
Based on the preliminary data, the proportion of HCWs who were hospitalized among those infected with SARS-CoV-2 ranges from 10% to 15%. A single-dose Ad26.COV2.S COVID-19 vaccine provided 89% protection against severe COVID-19. Table 1 shows the minimum sample size needed (number of vaccinated and unvaccinated essential workers: ratio 1:1) to detect VE ranging from 70% to 85%, with a precision of 10% or 20%.

Table 1: Minimum sample size needed to detect various VE estimates under different attack rates

| | | VE at 10% precision | | | | | VE at 20% precision | | | |
|---|---|---|---|---|---|---|---|---|---|---|
| | | 70% | 75% | 80% | 85% | | 70% | 75% | 80% | 85% |
| % hospitalized among cases | 5% | 11 818 | 9 538 | 7 404 | 5 422 | | 3 034 | 2 476 | 1 958 | 1 488 |
| | 10% | 5 770 | 4 672 | 3 640 | 2 676 | | 1 482 | 1 214 | 963 | 734 |
| | 15% | 3 754 | 3 050 | 2 384 | 1 760 | | 964 | 792 | 632 | 484 |
| | 20% | 2 746 | 2 240 | 1 758 | 1 302 | | 706 | 582 | 465 | 358 |

### 6.1.2 Unmatched or matched test negative case-control (TNCC)

WHO recommends TNCC to assess VE for symptomatic SARS-CoV-2 (PCR confirmed) infection. This design is mostly used for estimating influenza or rotavirus VE.

In this analytical approach, cases will be defined as **symptomatic** essential workers who test positive for SARS-CoV-2. The control group will comprise all symptomatic essential workers who test negative for SARS-CoV-2 infection. When cases and controls are tested for similar set of symptoms, this reduces differences due to health seeking behavior or access to care. In the absence of symptoms data, a proxy variable that measures health seeking behavior or exposure risk can be used to match cases and controls into 1;1, 1:2 or 1:3 case to control ratio. Matching in case-control studies, can be performed to reduce variance and improve precision but the matching variable should be accounted for in the analyses in order to avoid bias. Cases and controls will either be matched by time of enrolment (or this will be adjusted for in the analysis), as SARS-CoV-2 force of

infections constantly changing and different strains are emerging. Therefore, controls should be selected at a similar time period as cases.

Under this design, the odds of vaccination will be compared between cases and controls adjusting or after matching for potential confounders.

Descriptive statistics to assess the distribution of covariates among cases and controls will be conducted. Multivariable logistic regression will be used to estimate the vaccine effectiveness, defined as (1-AOR)*100 with adjustment for the following key variables of interest: age, gender, calendar time of vaccination and follow up, geographical location, race, socio-economic status and comorbidities.

Table 2 shows the minimum sample size needed to detect VE of 70% with a precision of 20% when vaccination coverage is either 30% or 60% among controls. Detecting VE estimates higher than 70% will require a slightly lower sample size.

Table 2: Minimum sample size needed (i.e. cases and controls) to detect various VE estimates under different attack rates

| | VE=70%, precision=20%, vaccine coverage among controls=30% | | | | VE=70%, precision=20%, vaccine coverage among controls=60% | | | |
|---|---|---|---|---|---|---|---|---|
| | Case: control ratio | | | | Case: Control ratio | | | |
| | 1:1 | 1:2 | 1:3 | 1:4 | 1:1 | 1:2 | 1:3 | 1:4 |
| N cases | 526 | 441 | 412 | 398 | 317 | 243 | 218 | 205 |
| N controls | 526 | 882 | 1236 | 1592 | 317 | 486 | 654 | 820 |

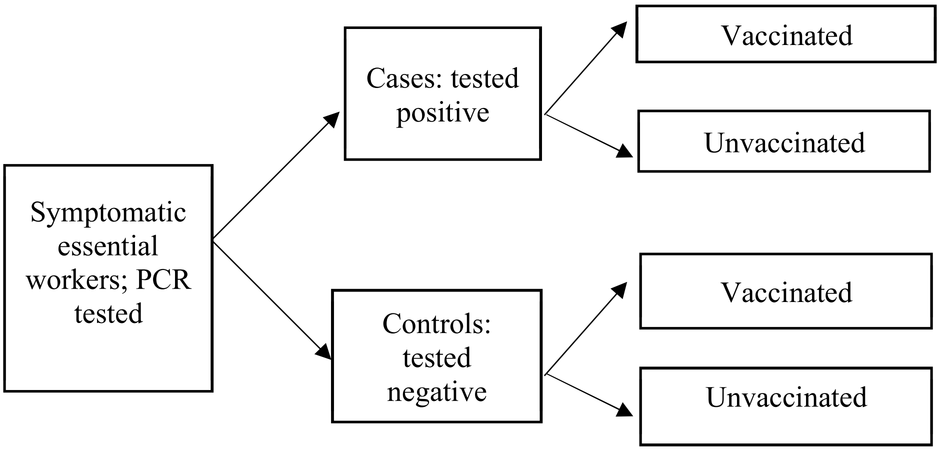


Figure 2: Test-negative case control design

## 6.2 Analysis of secondary endpoints

*Secondary endpoints:*

(i) *Rates of severe disease in HCW who are found to be RT-PCR positive at anytime up to 2 years post vaccination*

The rate of severe disease in vaccinated individuals will be expressed as a proportion with 95% binomial confidence intervals.

# 7 Safety analyses

Population: Safety population comprised vaccinated HCWs and the assessment will commence soon after vaccination.

A proportion of HCWs who reported at least one adverse event (AEs) irrespective of its severity, will be reported. Moreover, the rates of different types of AEs, expressed per 1000 vaccinated HCWs will be. The number of HCWs experiencing serious, rare but severe or life threatening events such as the development of severe blood clots will be summarised. These analyses will be stratified by age groups and gender.

# 8 Quality Assurance of Statistical Programming (As Applicable)

*A second review statistician will independently reproduce the primary analyses and summary statistics.*

To provide high quality code that is understandable, and allows for the reproduction of the analysis the following points will be followed.

The population to be used in a table or figure will be explicitly set at the start of a block of code that computes the output, ideally by looking up the population from the table of tables.

Any outputs will have the

- date and time included
- the name of the code file that produced the analysis
- the author

At the start of any code file there will be a set of comments that give

- the author
- the date and time of writing
- references to inputs and outputs
- reference to any parent code file that runs the child code file

# 9 References

World Health Organization. Evaluation of COVID-19 vaccine effectiveness: Interim guidance. March 2021

Dagan, N., Barda, N., Kepten, E., Miron, O., Perchik, S., Katz, M.A., Hernán, M.A., Lipsitch, M., Reis, B. and Balicer, R.D., 2021. BNT162b2 mRNA Covid-19 vaccine in a nationwide mass vaccination setting. New England Journal of Medicine.

Amit, S., Regev-Yochay, G., Afek, A., Kreiss, Y. and Leshem, E., 2021. Early rate reductions of SARS-CoV-2 infection and COVID-19 in BNT162b2 vaccine recipients. The Lancet, 397(10277), pp.875-877.

Mansournia MA, Hernan MA, Greenland S. Matched designs and causal diagrams. Int J Epidemiol. 2013;42:860-9. doi: 10.1093/ije/dyt083.

[1]WHO Case Definition guide: Accessed at https://www.who.int/publications/i/item/WHO-2019-nCoV-Surveillance_Case_Definition-2020.2 on 27 April 2021.

# 10 Listing of Tables, Listings and Figures
