# Supplementary material for: Safety evaluation of the single-dose Ad26.COV2.S vaccine among healthcare workers in the Sisonke study in South Africa: A phase 3b implementation trial
Source: PLoS Med. 2022 Jun 21;19(6):e1004024. doi: 10.1371/journal.pmed.1004024 (PMC9212139; doi:10.1371/journal.pmed.1004024)
Supplement: S4 Appendix — (DOCX) [file pmed.1004024.s004.docx]

**Supplementary Appendix 4:** Background incidence rates from health insurance claimant data

| **Age group** | **Exposure (years)** | **PE claimants** | **DVT claimants** | **PE claimants per 1000 patient years** | **DVT claimants per 1000 patient years** |
| --- | --- | --- | --- | --- | --- |
| <1 | 450579 | 0 | 2 | 0.00000 | 0.05326 |
| 01 to 04 | 2007637 | 2 | 13 | 0.01195 | 0.07770 |
| 05 to 09 | 2616480 | 1 | 16 | 0.00459 | 0.07338 |
| 10 to 14 | 2389197 | 11 | 27 | 0.05525 | 0.13561 |
| 15 to 19 | 1900395 | 46 | 74 | 0.29047 | 0.46727 |
| 20 to 24 | 1636939 | 76 | 130 | 0.55714 | 0.95300 |
| 25 to 29 | 2495220 | 213 | 297 | 1.02436 | 1.42833 |
| 30 to 34 | 3054384 | 326 | 431 | 1.28078 | 1.69330 |
| 35 to 39 | 3076714 | 357 | 552 | 1.39239 | 2.15295 |
| 40 to 44 | 2704038 | 408 | 689 | 1.81063 | 3.05765 |
| 45 to 49 | 2475798 | 426 | 703 | 2.06479 | 3.40739 |
| 50 to 54 | 2003489 | 375 | 677 | 2.24608 | 4.05493 |
| 55 to 59 | 1798185 | 463 | 775 | 3.08978 | 5.17188 |
| 60 to 64 | 1514325 | 490 | 763 | 3.88292 | 6.04626 |
| 65 to 69 | 1210257 | 485 | 733 | 4.80890 | 7.26788 |
| 70 to 74 | 976280 | 567 | 771 | 6.96931 | 9.47679 |
| 75 to 79 | 638018 | 483 | 563 | 9.08438 | 10.58904 |
| 80 to 84 | 358001 | 335 | 359 | 11.22902 | 12.03349 |
| 85+ | 222370 | 215 | 239 | 11.60228 | 12.89742 |
|  |  |  |  |  |  |

Abbreviations: PE - pulmonary embolism; DVT - deep vein thrombosis.

The data illustrates pulmonary embolism and deep vein thrombosis incidence among clients in a South African managed care organization (medical insurance scheme) prior to the COVID-19 pandemic.
