# Supplementary material for: Safety evaluation of the single-dose Ad26.COV2.S vaccine among healthcare workers in the Sisonke study in South Africa: A phase 3b implementation trial
Source: PLoS Med. 2022 Jun 21;19(6):e1004024. doi: 10.1371/journal.pmed.1004024 (PMC9212139; doi:10.1371/journal.pmed.1004024)
Supplement: S1 Table — (DOCX) [file pmed.1004024.s008.docx]

S1 Table: Twenty commonest non-reactogenicity adverse events reports

**Supplementary Table 1: Twenty commonest non-reactogenicity adverse events reports**

| **Adverse event** | **Frequency** |
| --- | --- |
| Nausea and /or Vomiting | 592 |
| Dizziness | 450 |
| Flu-like symptoms | 344 |
| Chills and rigors | 336 |
| Diarrhoea | 294 |
| Chest pain | 262 |
| Allergy like symptoms | 250 |
| Abdominal symptoms with or without diarrhoea | 190 |
| Cough | 169 |
| Numbness and Paraesthesia | 168 |
| Tachycardia | 114 |
| Allergy like symptoms | 102 |
| Itchiness | 93 |
| Tight chest or shortness of breath | 61 |
| Loss of taste or smell | 41 |
| Bruised on injection site | 35 |
| Lymphadenopathy | 33 |
| Insomnia | 31 |
| Elevated Blood pressure | 23 |
| Asthma exacerbation or bronchial spasm | 13 |
| Blister/s | 13 |
| Breast pain/swelling | 13 |

The 20 commonest non-reactogenicity AEs are shown in S1 Table
