# Supplementary material for: Safety evaluation of the single-dose Ad26.COV2.S vaccine among healthcare workers in the Sisonke study in South Africa: A phase 3b implementation trial
Source: PLoS Med. 2022 Jun 21;19(6):e1004024. doi: 10.1371/journal.pmed.1004024 (PMC9212139; doi:10.1371/journal.pmed.1004024)
Supplement: S2 Table — (DOCX) [file pmed.1004024.s009.docx]

S2 Table: Observed versus expected analysis of selected serious adverse events within 28 days of vaccination.

**S2 Table: Observed versus expected (O/E) analysis of selected serious adverse events within 28 days of vaccination.**

**Total person-years: 37 915**

| **Adverse event** | **Observed count** | **Observed incidence rate per 100,000 PY** | **Expected count** | **Expected incidence rate per 100,000 PY** | **O/E ratio (95% CI)** |
| --- | --- | --- | --- | --- | --- |
| **Vascular disorders** |  |  |  |  |  |
| Ischaemic stroke | 8 | 21.10 (10.55 -42.19) | 41.18 | 108.60 [*1] | 0.19 (0.08 – 0.38) |
| Pulmonary embolism | 6 | 15.82 (7.11 -35.22) | 8.44 | 22.26 [*S4 Table] | 0.71 (0.26 – 1.54) |
| Deep vein thrombosis | 4 | 10.55 (3.96 -28.11) | 12.20 | 32.19 [*S4 Table] | 0.33 (0.08 – 0.84) |
| Acute coronary syndrome | 1 | 2.64 (0.37 -18.72) | 85.69 | 226.00 [*2] | 0.01 (0.00 – 0.07) |
| Thrombotic thrombocytopenic syndrome | 1 | 2.64 (0.37 -18.72) | 0.33 | 0.88 [*3] | 3.0 (0.08-16.70) |
| **Neurological disorders** |  |  |  |  |  |
| Bell’s palsy | 2 | 5.27 (1.32 -21.09) | 8.53 | 22.5 [*4] | 0.23 (0.03-0.85) |
| Guillain-Barré syndrome | 4 | 10.55 (3.96 -28.11) | 0.31 | 0.83 [*4] | 12.71 (3.46-32.54) |
| Transverse myelitis | 0 | - | 28.14 | 29.7 [*4] | 0.08 (0.01 – 0.27) |
| Seizure | 1 | 2.64 (0.37 -18.72) | 27.79 | 73.3 [*4] | 0.04 (0.00-0.20) |
| **Cardiac disorders** |  |  |  |  |  |
| Myocarditis | 1 | 2.64 (0.37 -18.72) | 8.34 | 22 [*4] | 0.12 (0.003 – 0.67) |
|  |  |  |  |  |  |

Abbreviations: O/E - observed incidence divided by expected incidence; PY -person-years; 95% CI - 95% confidence interval.

*Reference to literature from which the background incidence was derived.

**References**

1. Walker R, Whiting D, Unwin N, Mugusi F, Swai M, Aris E, et al. Stroke incidence in rural and urban Tanzania: a prospective, community-based study. Lancet Neurol. 2010 Aug;9(8):786-92. doi: 10.1016/S1474-4422(10)70144-7.

2. Pitsavos C, Panagiotakos DB, Antonoulas A, Zombolos S, Kogias Y, Mantas Y, et al. Epidemiology of acute coronary syndromes in a Mediterranean country; aims, design and baseline characteristics of the Greek study of acute coronary syndromes (GREECS). BMC Public Health. 2005;5(1):23. doi:10.1186/1471-2458-5-23.

3. Burn E, Li X, Kostka K, et al. Background rates of five thrombosis with thrombocytopenia syndromes of special interest for COVID-19 vaccine safety surveillance: Incidence between 2017 and 2019 and patient profiles from 38.6 million people in six European countries. Pharmacoepidemiol Drug Saf. 2022;31(5):495-510. doi:10.1002/pds.5419.

4. Brighton Collaboration. Brighton Collaboration Case Definitions. (Cited 16 November 2021). Available from: https://brightoncollaboration.us/category/pubs-tools/case-definitions/
