# Supplementary material for: Safety evaluation of the single-dose Ad26.COV2.S vaccine among healthcare workers in the Sisonke study in South Africa: A phase 3b implementation trial
Source: PLoS Med. 2022 Jun 21;19(6):e1004024. doi: 10.1371/journal.pmed.1004024 (PMC9212139; doi:10.1371/journal.pmed.1004024)
Supplement: S3 Table — (DOCX) [file pmed.1004024.s010.docx]

S3 Table: Mortality in the Sisonke study

**S3 Table: Mortality in the Sisonke study**

|  | **Overall (n=157)** | **Cause known (n=66)** | **Unknown (n=91)** |
| --- | --- | --- | --- |
| Male sex, n (%) | 60 (38.0%) | 28 (41.8%) | 32 (35.2%) |
| Age (years), median (IQR) | 48 (40 – 47) | 48 (41 – 58) | 48 (40 – 57) |
| Time to death, median (IQR) |  |  |  |
| ≤ 28 days, n (%) | 53 (33.7%) | 19 (35.8%) | 34 (64.2%) |
| 28 days to ≤ 3 months | 88 (56.1%) | 42 (47.7%) | 46 (52.3%) |
| > 3 months | 16 (10.2%) | 5 (31.2%) | 11 (31.2%) |
| Cause listed as non-natural, n (%) | 42 (26.6%) | 15 (22.4%) | 27 (29.7%) |
| Comorbidities, n (%) |  |  |  |
| Hypertension | 48 (30.4%) | 21 (31.3%) | 27 (29.7%) |
| Diabetes | 32 (20.3%) | 13 (19.4%) | 19 (20.9%) |
| HIV | 20 (12.7%) | 12 (17.9%) | 8 (8.8%) |
| Total number comorbidities | 90 (57.0%) | 44 (65.7%) | 46 (50.6%) |

 Abbreviations: IQR - interquartile range; HIV - Human Immunodeficiency Virus infection.,
