# Supplementary material for: Safety evaluation of the single-dose Ad26.COV2.S vaccine among healthcare workers in the Sisonke study in South Africa: A phase 3b implementation trial
Source: PLoS Med. 2022 Jun 21;19(6):e1004024. doi: 10.1371/journal.pmed.1004024 (PMC9212139; doi:10.1371/journal.pmed.1004024)
Supplement: Attachment — Submitted filename: takuva.pdf [file pmed.1004024.s011.pdf]

This paper describes the profile of adverse events in health care workers in South Africa following vaccination against COVID–19.

I was asked for a statistical report and I interpret that to include all aspects of the design and conduct of the study.

Points of detail

Page 7 With the sort of numbers involved here almost any difference is going to achieve statistical significance so I think it would be helpful to present effect sizes with confidence intervals.

Page 9 Although it is true that the figure for TTS (2.4) is greater than one I think the confidence interval from 0.3 to 8.7 should lead to a more cautious statement.

Page 10 I imagine it probably is the largest study but inserting 'to our knowledge' would be safer.

Page 16, Table 1 I found I kept losing track of which p–value belonged with which frequencies. Removing the horizontal rules except between characteristics would make it clearer.

Page 18, Table 2 What does the asterisk after Sagittal sinus thrombosis mean?

Page 22, Table 3 What does the double asterisk after Pulmonary embolism mean? It also appears in Supplementary Table 2.

Page 23, Figure 1 It is rather hard to distinguish all of the curves here. I would be inclined to use nine sub–plots. That would also have the advantage of being more friendly for readers with colour vision anomalies. It is true that some of the detail would be harder to see in smaller plots but I think it is the visual impression we are interested in here.

Page 24, Figure 2 The two blues are quite hard to distinguish, I assume the RMS one is the upper in the plot as well as the legend. I suggest using less saturation for one of them.

Points of more substance

Serious adverse events

If we assume that the primary interest for people contemplating vaccination is the serious adverse events then the disproportionality analysis will be im-

portant. I think we could use more detail here. For each of the conditions listed in Table 3 we need to know which sources were available for generating the expected count and if there was more than one then what each was and what the combined values was. The caption states that the mid–point was used, does this mean the value half–way between the minimum and the maximum, known in some older texts as the centre? I was not sure what the force of the caption comment that stratification by age group was not used. Did the calculation of expected not take into account the age–sex breakdown of the cohort here and the population sourced being used?

Other adverse events

Some of the people who experienced an event not classified as serious appear to have been quite seriously affected. On page 8 we learn that 32% were unable to perform usual activities and 2% had to receive some form of hospital care. I think we need more detail here. For instance Supplementary Table 1 could show us each event broken down by the effect on the participant and not just the frequency of any event however mild. The 8% who had not recovered by day seven are also a concern. The clinical reviewers may have a view on this but as a lay person who has received numerous vaccinations I expect sore arms and mild fever but something which took me to hospital would be quite alarming.

Categorising age

usually I would comment critically on categorising an essentially continuous variable which wastes information (Altman and Royston, 2006; Royston et al., 2006) and leads to models which are often implausible as they predict the effect remaining flat within categories and then jumping to a new value at the category boundary. In this case the authors are not fitting complicated models so the point is less important but the categories used do not seem consistent for instance between Table 1 and Figure 2. Is there some reason here which I am missing?

Summary

Some details and also a request for more comprehensive reporting. Data–sets of this size are going to be hard to come by and the community needs to be able to extract the maximum from it.

Michael Dewey

References

D G Altman and P Royston. The cost of dichotomising continuous variables. *British Medical Journal*, 332:1080, 2006.

P Royston, D G Altman, and W Sauerbrei. Dichotomizing continuous predictors in multiple regression: a bad idea. *Statistics in Medicine*, 25:127–141, 2006.
