# Supplementary material for: Safety evaluation of the single-dose Ad26.COV2.S vaccine among healthcare workers in the Sisonke study in South Africa: A phase 3b implementation trial
Source: PLoS Med. 2022 Jun 21;19(6):e1004024. doi: 10.1371/journal.pmed.1004024 (PMC9212139; doi:10.1371/journal.pmed.1004024)
Supplement: Attachment — Submitted filename: Response to Reviewers_ Sisonke PLOS Med.docx [file pmed.1004024.s012.docx]

Caitlin Moyer, Ph.D.	25 March 2022

Associate Editor

PLOS Medicine

Dear Dr Caitlin Moyer,

**Re: Manuscript Number PMEDICINE-D-21-04884R1 – Response to Reviewers**

We are grateful to you and the Reviewers for giving us the opportunity to revise our manuscript, now entitled *‘Safety evaluation of the single-dose Ad26.COV2.S vaccine among healthcare workers in the Sisonke study in South Africa: a phase 3b implementation trial’*

Please find below our response to the Editor’s and Reviewers’ comments, with additional text highlighted in **bold**. We have also uploaded a marked-up copy and an unmarked version of the revised manuscript. Line numbers are for the clean copy of the revised manuscript:

**Requests from the Editor**
1. Title: Please revise your title according to PLOS Medicine's style. Your title must be nondeclarative and not a question. It should begin with main concept if possible. "Effect of" should be used only if causality can be inferred, i.e., for an RCT. Please place the study design ("A randomized controlled trial," "A retrospective study," "A modelling study," etc.) in the subtitle (ie, after a colon).

**Response:** This has been revised. The manuscript is now entitled: *‘****Safety evaluation of the single-dose Ad26.CoV2.S vaccine among healthcare workers in the Sisonke study in South Africa: a phase 3b implementation trial’***

2. Financial disclosure: Thank you for indicating that the funders had no role in the study design, data collection and analysis, decision to publish, or preparation of the manuscript. Please add to your statement in the Financial Disclosure section of the submission form: the full names of each funder, specific grant numbers, the initials of authors who received each award, and URLs to sponsors’ websites.

**Response:** We have added additional detail to the financial disclosure section, as requested.

3. Main text: Please provide line numbers running continuously through the manuscript with the revised version.

**Response:** Line numbers have been included.

4. Abstract: Please structure your abstract using the PLOS Medicine headings (Background, Methods and Findings, Conclusions) Please combine the Methods and Findings sections into one section, “Methods and findings”.

**Response:** We have reformatted the abstract accordingly.

5. Abstract: Background: Provide a sentence or two of context of why the study is important. The final sentence should clearly state the study question.

**Response:** We have added the following sentence to the background to provide context, and we have clearly stated the aim of the study at the end of this section [Page 3, Line 65, section: Abstract].

***‘Real-world evaluation of the safety profile of vaccines post licensure is crucial to accurately characterize safety beyond clinical trials, support continued use and thereby improve public confidence.’***

6. Abstract: Methods and Findings: Please describe the study design, and population and setting.

**Response:** The study design, population and setting are included in this section of the abstract, as requested.

7. Abstract: Methods and Findings: For the following two statements, please provide the numbers/percentages associated with these reported AEs: “The commonest reactogenicity events were headache and body aches, followed by injection site pain and fever…” and “Serious AEs and AEs of special interest including vascular and nervous system events, immune system disorders and deaths occurred at lower than the
expected population rates.”

**Response:** We have added additional numbers to the ‘Methods and Findings’ section of the abstract [Page 3, Line 81, section: Abstract].

8. Abstract: Methods and Findings: In the last sentence of the Abstract Methods and Findings section, please describe the main limitation(s) of the study's methodology.

**Response:** We have added a limitation sentence to the abstract [Page 3, Line 88, section: Abstract]: ***‘A limitation of the study was the single-arm design, with limited routinely collected morbidity comparator data in the study setting.’***

9. Abstract: Conclusions: Please address the study implications without overreaching what can be concluded from the data; the phrase "In this study, we observed ..." may be useful.

**Response:** We have revised the ‘Conclusion’ in the abstract accordingly [Page 32, Line 91, section: Abstract]: *‘****We observed similar patterns of AEs as in phase 3 trials. AEs were mostly expected reactogenicity signs and symptoms. Furthermore, SAEs were rare and occurred below expected rates.*** *The single-dose Ad26.COV2.S vaccine demonstrated an acceptable safety profile supporting the continued use of this vaccine in our setting.’*

10. Author summary: At this stage, we ask that you include a short, non-technical Author Summary of your research to make findings accessible to a wide audience that includes both scientists and non-scientists. The Author Summary should immediately follow the Abstract in your revised manuscript. This text is subject to editorial change and should be distinct from the scientific abstract. Please see our author guidelines for more information: <https://journals.plos.org/plosmedicine/s/revising-your-manuscript#loc-author-summary>

**Response:** We have added an Author Summary as per the guidance [Page 32, Line 96, section: Author Summary].

11. Main text: Please use square brackets for in-text citations of references, placed before the sentence punctuation, for example [1,2].

**Response:** The in-text references have been reformatted.

12. Background: Please title this section of the main text “Introduction” and please conclude the Introduction with a clear description of the study question or hypothesis.

**Response:** This has been revised. The last sentence of the first paragraph of the introduction now includes the following sentence [Page 5, Line 137, section: Introduction].

: ***‘The study aimed to assess the safety and effectiveness of the Janssen Ad26.CoV2.S vaccine among HCWs in South Africa.’***

The last sentence of the Introduction section has been modified to avoid repetition [Page 62, Line 1635, section: Introduction]. It now reads as: *‘The Sisonke study enrolled almost half a million HCWs, providing an opportunity to further evaluate the safety of the Ad26.COV.S2 vaccine in an expanded population. Here we present the safety data.’*

13. Methods: Please report your study according to the relevant guideline, which can be found here: <http://www.equator-network.org/>
Please ensure that the study is reported according to the CONSORT guidelines, and please complete the CONSORT checklist and ensure that all components of CONSORT are present in the manuscript.
When completing the checklist, please use section and paragraph numbers, rather than page numbers.
Please add the following statement, or similar, to the Methods: "This study is reported as per the Consolidated Standards of Reporting Trials (CONSORT) guideline (S1 Checklist)."

**Response:** We have carefully reviewed the CONSORT checklist and have ensured that all relevant sections have been addressed in the manuscript. The completed CONSORT checklist is also attached.

14. Methods: Please provide additional description of inclusion and exclusion criteria as indicated in more detail online (PACTR/clinical [trials.gov](http://trials.gov/) registries). Please explicitly how participants were recruited, and mention that informed written consent of participants was obtained. Please comment on whether it is possible there were individuals included who had received other vaccines to prevent SARS-CoV-2 infection. Please mention/provide numbers to inform if there were eligible individuals who were contacted but declined to participate. Please include a participant flow diagram.

**Response:** We have included additional description of recruitment as follows [Page 6, Line 174, section: Methods]: **‘*All 1 250 000 HCWs targeted by phase 1 of the national COVID-19 Vaccine Rollout Strategy, were invited for vaccination. To participate HCWs were required to register on the national Electronic Vaccination Data System (EVDS) and provide electronic consent if they were age 18 and older.’***

Considering no COVID-19 vaccines were available in South Africa at the time of the Sisonke study, and vaccines were also registered on the centralized EVDS system, we think it is highly unlikely that participants may have received another vaccine during the enrolment period. We have now also provided a link to the study protocol that further details the eligibility criteria. Out of the estimated 1 250 000 HCWs invited to participate, 477,234 volunteered to participate.

15. Methods: Please include the study protocol document and the analysis plan, with any amendments, as Supporting Information to be published with the manuscript if accepted.

**Response:** These documents have now been included.

16. Methods: Please include a copy of the electronic case report form designed for the study as a supporting information document. Please provide additional information for the verification of SAEs and AEs of medical concern, and for spontaneous reports via unsolicited HCW communication.

**Response:** The CRF is now attached as an appendix. We have also added the following description [Page 7, Line 212, section: Methods]: ‘***Telephonic follow up to the participant and attending health care provider was established as part of case investigation. Case reports for safety events of concern were collated from these telephonic interviews, medical records and results from laboratory and imaging investigations.’***

17. Methods: Please provide a list of those events considered as serious AEs/AEs of special interest.

**Response:** The definition of Serious AEs has been included in the text.

*‘Adverse event reports were processed daily and screened for serious AEs (SAE)* [Page 7, Line 229, section: Methods]*:* ***any AE that results in death, is life-threatening, requires inpatient hospitalization or prolongation of existing hospitalization, results in persistent or significant disability/incapacity, or is a congenital anomaly/birth defect, is considered serious by the investigator.’***

The pre-specified list of AESIs is referenced in the methods section (link included). In the Results section, Table 2 lists the SAE/AESIs reported in the study and the frequencies.

18. Methods: Missing data have the potential to introduce bias in your study. Please explain how you have dealt with missing data.

**Response:** There is no formal statistical method that was used to handle missing data. We have included this statement in the statistical analysis section [Page 8, Line 265, section: Methods]: ***‘There were no formal statistical methods that were used to handle missing data.’***

We acknowledge a limitation of reporting bias however we did not measure missingness of the data. In the limitations sections we further expand on this limitation.

19. Methods: Please describe how individual SAEs were selected for this analysis: “For selected SAEs, disproportionality analysis was conducted…”

**Response:** Individual SAEs such as Thrombosis with Thrombocytopaenia Syndrome and Guillain-Barré Syndrome were selected for the disproportionality analysis based on frequency of occurrence and safety signal reports in previous and ongoing studies and surveillance systems like VAERS.

20. Methods: Statistical analysis: “Participants reporting and not
reporting AEs were compared by baseline characteristics.” Please describe the collection of baseline characteristic data, and please describe how these data were incorporated into analyses. Please explain the categorization for age.

**Response:** Baseline data were collected mainly from participant self-report questionnaires completed at time of expression of interest to participate in Sisonke and through linkage with the vital registration system using the unique national ID number.

For age categorization we considered that as this was a working population, the age would range from 18 to 65 years. Age 65 is the age of retirement in South Africa. To enable balanced numbers within different age categories, the age groups used in the manuscript were adopted.

21. Methods: Statistical analysis: “Available background incidence rates were used including a medically insured population in South Africa (pulmonary embolism and deep venous thrombosis), Tanzanian population-based cohort study (neurological events such as stroke)
and European population databases (10, 15–18).” Please provide more information, including how expected count/incidence were obtained for Table 3.

**Response:** We have included in the statistical analysis section the following text to further clarify this analysis [Page 8, Line 253, section: Methods]: *‘the observed (O) number of reported cases was compared to the expected (E) number based on background incidence rates and the O/E ratio with 95% confidence interval was calculated.* ***The 95% confidence intervals for the O/E ratio were calculated as follows: (i) the 95% confidence interval of the observed number of events was calculated assuming a Chi square distribution; (ii) each of the lower and upper limits of the confidence interval in (i) were divided by the number of expected events to produce the 95% confidence interval for the O/E ratio****.’*

22. Results: Please clarify here if all post-vaccination follow-up contacts provided additional data (on whether AEs resolved or not), and if not, please mention numbers lost to follow up. “Follow up at day seven post vaccination indicated that 92% of participants reporting AEs had either completely recovered or were recovering. The remaining 8% of participants were contacted by the safety team and, if required, referred for care.”

**Response:** All participants received a text with an electronic CRF link to report AEs soon after they were vaccinated. All reported AEs were clinically screened and if indicated contact was made by the safety team for further evaluation. A participant not reporting was not considered lost to follow-up but as a participant who did not report an AE.

23. Results: Please present numerators and denominators for percentages, at least in the Tables if not in the main text of the Results [not necessarily each time they're mentioned].

**Response:** Denominators and percentages are now included in the Tables. For Table 1, the Total N is the denominator. For Table 2 we have reported person-years as the denominator and we are not reporting proportions. In Table 3, person-years are also used.

24. Results: “One in five (19%) AEs were not consistent classified as reactogenicity events (Supplementary Table 1).” Please clarify in the text what is meant here (for example, if these were classified as serious adverse events).

**Response:** Further clarification has been added to the text as follows [Page 7, Line 202, section: Methods]: ***‘Reactogenicity events are pre-specified common AEs expected soon after vaccination and include systemic events such as headache, fever, myalgia, arthralgia, malaise, nausea, chills and local events such as pain, erythema and induration.’***

25. Results/ Figure 2: Please provide additional details regarding the comparison with the local employee group life assurance data.

**Response:** To be eligible for group life benefits there is an implicit actively-at-work selection effect. A comparison for Sisonke mortality rates therefore warrants careful consideration. The mortality of the general population as measured via the Rapid Mortality Surveillance is expected to be higher than that of the members of group life assurance due to what is known as ‘the healthy worker effect’. Since the Sisonke cohort is comprised of healthcare workers, the mortality rates of the group-life insured population (i.e., working population) provides a better comparator group.

26. Discussion: Please be sure to present and organize the Discussion as follows: a short, clear summary of the article's findings; what the study adds to existing research and where and why the results may differ from previous research; strengths and limitations of the study; implications and next steps for research, clinical practice, and/or public policy; one-paragraph conclusion.

**Response:** Thank you, the Discussion section has been reorganized to follow the above arrangement.

**Reviewers’ Comments**

**Reviewer #1:**

Reviewer 1 provided comments in an attachment. We have included his comments and responses in this document:

This paper describes the profile of adverse events in health care workers in South Africa following vaccination against COVID–19. I was asked for a statistical report and I interpret that to include all aspects of the design and conduct of the study.

**Points of detail**

**Page 7** With the sort of numbers involved here almost any difference is going to achieve statistical significance so I think it would be helpful to present effect sizes with confidence intervals.

**Response:** We concur with Reviewer 1 that a large sample size will always produce statistically significant p-values for differences that are not always clinically meaningful. We have therefore removed the p-values from Table 1 and have added 95% confidence intervals instead.

**Page 9** Although it is true that the figure for TTS (2.4) is greater than one I think the confidence interval from 0.3 to 8.7 should lead to a more cautious statement.

**Response:** We have revised this statement to read as follows [Page 11, Line 347, section: Results]: *‘Out of the AEs examined,* ***TTS seemed to occur at a rate greater than the baseline comparison although the 95% confidence interval was wide and crossed one (O/E ratio 2.4; 95%CI 0.3-8.7)*** *and GBS occurred at rates greater than the baseline comparison population (O/E ratio 5.1; 95%CI 1.4-13.0 respectively).’*

**Page 10** I imagine it probably is the largest study but inserting ‘to our knowledge’ would be safer.

**Response:** As suggested, we have added this language to the beginning of the discussion section [Page 12, Line 401, section: Discussion].

**Page 16, Table 1** I found I kept losing track of which *p*–value belonged with which frequencies. Removing the horizontal rules except between characteristics would make it clearer.

**Response:** As mentioned above, we have decided to remove p-values and add 95% confidence intervals instead to Table 1.

**Page 18, Table 2** What does the asterisk after Sagittal sinus thrombosis mean?

**Response:** The asterixis has been removed as it was a note for the authors in the draft versions during the writing stage.

**Page 22, Table 3** What does the double asterisk after Pulmonary embolism mean? It also appears in Supplementary Table 2.

**Response:** Thank you! The asterixis has now been removed as it was a note for the authors in the draft versions during the writing stage.

**Page 23, Figure 1** It is rather hard to distinguish all of the curves here. I would be inclined to use nine sub–plots. That would also have the advantage of being more friendly for readers with colour vision anomalies. It is true that some of the detail would be harder to see in smaller plots but I think it is the visual impression we are interested in here.

**Response:** Instead of plotting nine sub-plots, we have used broken lines to make the curves more distinguishable. We hope that this is acceptable to the reviewer. We wanted to ensure that all common reactogenicity events can be compared and interpreted in one figure.

**Page 24, Figure 2** The two blues are quite hard to distinguish, I assume the RMS one is the upper in the plot as well as the legend. I suggest using less saturation for one of them.

**Response:** Thank you. We have decided to change one shade of blue to black.

**Points of more substance**

**Serious adverse events**

If we assume that the primary interest for people contemplating vaccination is the serious adverse events then the disproportionality analysis will be important. I think we could use more detail here. For each of the conditions listed in Table 3 we need to know which sources were available for generating the expected count and if there was more than one then what each was and what the combined values was. The caption states that the mid–point was used, does this mean the value half–way between the minimum and the maximum, known in some older texts as the centre? I was not sure what the force of the caption comment that stratification by age group was not used. Did the calculation of expected not take into account the age–sex breakdown of the cohort here and the population sourced being used?

**Response:** The sources used to generate the expected counts are cited as references. The mid-point was used, meaning the value half-way between the minimum and the maximum of the estimates. Stratification by age-group and sex was desired however despite an extensive literature search, it was a challenge to find expected values stratified by these parameters.

**Other adverse events**

Some of the people who experienced an event not classified as serious appear to have been quite seriously affected. On page 8 we learn that 32% were unable to perform usual activities and 2% had to receive some form of hospital care. I think we need more detail here. For instance Supplementary Table 1 could show us each event broken down by the effect on the participant and not just the frequency of any event however mild. The 8% who had not recovered by day seven are also a concern. The clinical reviewers may have a view on this but as a lay person who has received numerous vaccinations I expect sore arms and mild fever but something which took me to hospital would be quite alarming.

**Response:** To clarify further, the text has been revised to read ‘***The remaining 8% of participants were contacted by the safety team. Attempts were made to contact all these participants individually by the safety team (three telephonic calls at least one day apart). Those that were contactable were captured and if indicated, referred for further care***.***’*** [Page 9, Line 299, section: Results].

As the study did not provide specific clinical care, participants were instructed to seek further treatment for AEs in addition to reporting. A day-7 follow-up contact was then done subsequent to the AE report via a short electronic CRF link sent by text. At this day-7 contact, 8% reported not having recovered and attempt was made to contact all these participants individually by the safety team (three telephonic calls at least one day apart). Those that were contactable were captured and referred for further care.

**Categorising age**

Usually I would comment critically on categorising an essentially continuous variable which wastes information (Altman and Royston, 2006; Royston et al., 2006) and leads to models which are often implausible as they predict the effect remaining flat within categories and then jumping to a new value at the category boundary. In this case the authors are not fitting complicated models so the point is less important but the categories used do not seem consistent for instance between Table 1 and Figure 2. Is there some reason here which I am missing?

**Response:** Thank you, we take note of this shortcoming when categorising continuous variables. The categories in Table 1 were defined by the study team based on that this was a working population, where the age would range from 18 to 65 years. Age 65 is the age of retirement in South Africa. To also enable balanced numbers within different age categories, these age groups used in the manuscript were adopted. However, the age categories in Figure 2 have been historically used to produce age-standardised rates in South Africa. Therefore, in Sisonke we had to calculate age-specific rates for these pre-specified categories.

**Summary**

Some details and also a request for more comprehensive reporting. Data–sets of this size are going to be hard to come by and the community needs to be able to extract the maximum from it.

Michael Dewey

**Response:** Thank you for this summary and helpful comments, Michael!

**Reviewer #2:**

S. Takuva et al., reported here the results of an open label implementation study that assess the safety (and the effectiveness) of one shot of Ad26.CoV2.S vaccine of janssen among Healthcare workers (HCWs) in South Africa. This manuscript is focused on the presentation of safety data only.
It is a very impressive implementation study that included in real life conditions an half of a million of HCWs.
The manuscript is well written and easy to read.
The introduction is concise and presented well the situation.

**Response:** Thank you for this positive feedback!

I have few comments:
-in the methods section, (p 5) please provide details about the exclusion of pregnant women (there is a pregnancy test performed? what could be challenging in study of this size; if not just precise that women with a known pregnancy were excluded).

**Response:** We agree it is more accurate to refine the language. The statement now reads as follows [Page 6, Line 179, section: Methods]: *‘****Known pregnant women and breastfeeding women were excluded due to a lack of sufficient safety data at that time.’***

-in the results section, since the numerators are not notified in the text, please propose to the reader to see in the table 1, as it is only the percentage is available ("the majority were women (74.9%)").

**Response:** At the end of the first paragraph of the Results section, we now refer to ***‘Table 1’***, where the numerators and denominators are now included. [Page 92, Line 278, section: Results].

In the section "SAE" of the results section page 8, it is written "114 reported by women and 25 by men" 114+25= 139, only 138 SAEs excluding death were reported, please change.  In this same paragraph, please specify at which time after vaccination the SAEs outcome were evaluated?

**Response:** Thank you for picking up this mistake. The figures have been revised: 113 reports for females and 25 reports for males (113+25=138). We have also adjusted the percentages accordingly. In the paragraph we have added the statement [Page 10, Line 315, section: Results]: ‘**SAEs were followed up by safety staff until resolution.’**

For the 9 deaths (6.5%), I suggest to precise that it represented 9/10, 279 (0.09%) of people that declared an AE.

**Response:** We have revised the sentence accordingly. It now reads [Page 11, Line 359, section: Results]: ‘***A total of 157 (1.5% of participants reporting an AE) non-COVID-19 related deaths (167 per 100,000 person-years) were identified via the active linkage system with the national population registry.***’

-references in the reference list are sometime not completed

**Response:** We have reviewed the references and have made required edits. We have also updated references with the published effectiveness results from the Sisonke study: Bekker LG, Garrett N, Goga A, Fairall L, Reddy T, Yende-Zuma N, et al. Sisonke Protocol Team; Sisonke Study Team. Effectiveness of the Ad26.COV2.S vaccine in health-care workers in South Africa (the Sisonke study): results from a single-arm, open-label, phase 3B, implementation study. Lancet. 2022 Mar 19;399(10330):1141-1153.

This work is very interesting and indeed shows that, including in low and middle income countries, surveillance at large scale is possible, what is crucial to achieve confidence in vaccines.

**Response:** Thank you for this positive feedback!

**Reviewer #3:**

I thoroughly enjoyed reading the "Safety of the single-dose Ad26.CoV2.S vaccine among healthcare workers in the phase 3b Sisonke study in South Africa" by Takuva et al. It was clearly written in excellent English, with a format and logic that was cohesive and easy to follow.  Their sampling and selection criteria is clear, and their analysis follows standard methods.  The reporting of their results is clear, and in their discussion and limitations they follow a logical expansion of the topic.

**Response:** Thank you for this great feedback!

The only suggestions that I have to improve the quality of their excellent work are as follows:
1- Given the high percentage of females in the study, a brief explanation of whether they over sampled for females would be clarifying.

**Response:** The demographics in the Sisonke study reflect the sex balance in the health care worker population in South Africa, so we do not think that we oversampled women.

2- In Tables 2 and 3, if there could be an additional column which indicates the incidence of adverse events in other large studies of the safety Ad26.CoV2.S (perhaps Ref. 7 & 8) it would allow for rapid comparison of results.

**Response:** We have discussed the clinical trial adverse events data in paragraphs 2 and 3 of the discussion section.

3- Several minor typo mistakes, which fall under the editorial corrections.

**Response:** Additional proof-reading has been conducted. Thank you.
