# Supplementary material for: Safety evaluation of the single-dose Ad26.COV2.S vaccine among healthcare workers in the Sisonke study in South Africa: A phase 3b implementation trial
Source: PLoS Med. 2022 Jun 21;19(6):e1004024. doi: 10.1371/journal.pmed.1004024 (PMC9212139; doi:10.1371/journal.pmed.1004024)
Supplement: Attachment — Submitted filename: Responses to reviewers.RV2.docx [file pmed.1004024.s013.docx]

Caitlin Moyer, Ph.D.	12 May 2022

Associate Editor

PLOS Medicine

Dear Dr Caitlin Moyer,

**Re: Manuscript Number PMEDICINE-D-21-04884R2 – Response to editorial and remaining reviewer’s comments**

We are grateful to you and the Reviewers for providing additional comments on our manuscript, entitled *‘Safety evaluation of the single-dose Ad26.COV2.S vaccine among healthcare workers in the Sisonke study in South Africa: A phase 3b implementation trial’.*

Please find below our responses to the comments. We have uploaded a marked-up copy and a clean version of the revised manuscript. Line numbers refer to the clean version.

**Requests from the Editor**
1. Response to Reviewers: Please completely address the remaining point of Reviewer 1 by providing additional details on effects on participants for adverse events reported in Supporting Information Table 1.

**Response:** We understand the importance of Reviewer 1’s question. Unfortunately, we do not have adequate data to provide detailed severity grades for each and every adverse event reported. We are however able to provide aggregate severity grades for all AEs reported (see line 300 to 304, page 9) and specific severity grades for SAEs and AESIs. The database and collection was designed to interrogate further those AEs classified as serious or of special interest.

2. Title: Please capitalize the first letter of the first word of the subtitle, and please update this in both the manuscript text and the manuscript submission system: “Safety evaluation of the single-dose Ad26.COV2.S vaccine among healthcare workers in the Sisonke study in South Africa: A phase 3b implementation trial”

**Response:** The subtitle has been revised accordingly.

3. Data availability statement: The Data Availability Statement (DAS) requires revision. If the data are owned by a third party (The National Department of Health, in this case), please note whether the data may be made available upon request or application, and state the owner of the data set and contact information for data requests (as you have done already: Office of the Director-General, E-mail: [DG@health.gov.za](mailto:DG@health.gov.za)).

We request that the analytical code be made available, without restrictions on access, in a public repository or included as Supporting Information at the time of article publication, provided it is legal and ethical to do so. We note that the Protocol and Analysis Plan are included as supporting information files already, and these files do not need to be mentioned here.

Please update this statement with information needed to access the analytical code. Please note that a study author may not serve as the point of contact for access. Please see the policy at <http://journals.plos.org/plosmedicine/s/data-availability> and FAQs at
<http://journals.plos.org/plosmedicine/s/data-availability#loc-faqs-for-data-policy>

**Response:** As suggested, we have now updated the DAS and have provided the analytical code as supplemental information.

4. Abstract: Methods and Findings: Please provide the trial registration information in the Abstract.

**Response:** This has now been provided.

5. Abstract: Methods and Findings: Please clarify “...among all eligible HCWs in South Africa registered in the national Electronic Vaccination Data System (EVDS)…” or similar.

**Response:** We have added this clarification to the abstract.

6. Abstract: Methods and Findings: At line 77, the number of serious AEs is reported as 139, and at line 85, this is reported as 138.

**Response:** This has now been revised to 138 (1.3%). Thank you for picking this up.

7. Abstract: Conclusions: Line 91-93: Given the finding in Table 3 for TTS and GBS, it may be more accurate to phrase this as “most SAEs occurred below expected rates.”

**Response:** This has been revised as suggested.

8. Author summary: Line 108-111: Please revise to: “The majority of adverse events reported…” and please revise the following sentence to “Most serious adverse events…”

**Response:** The sentence has been revised as suggested.

9. Methods: Please add the following statement, or similar, to the Methods: "This study is reported as per the Consolidated Standards of Reporting Trials (CONSORT) guideline (S1 Checklist)."

**Response:** This statement has been added. See line 180, page 6.

10. Methods: Line 173-174: Please reference the copy of the study protocol included as a supporting information file and refer to it here (Supplementary Appendix 2). Please cite the online version of the protocol in the reference list, rather than including a web link here.

**Response:** The reference to the protocol (Supplementary Appendix 1) has now been included and the web link has been removed. The protocol now becomes Supplementary Appendix 1 as it is referenced first, and the Case Report Form is now Supplementary Appendix 2.

11. Methods: Statistical analysis: Line 239:

Please mention If a prospective analysis plan was used in designing the study, for example, Supplementary Appendix 3, and please reference that file here. Please describe in the text if any changes to the analysis plan were made, and when.

**Response:** As suggested, we have revised the first paragraph of the statistical analysis section and provide a reference to the Statistical Analysis Plan:

***‘A prospective analysis plan was used in designing the study (see Supplementary Appendix 3).*** *For descriptive statistics, counts and proportions were used for categorical variables, and medians and interquartile ranges for quantitative variables.* ***The baseline characteristics sex, age, previous COVID-19 status and presence of comorbidities were compared between participants reporting AEs and those not reporting AEs using descriptive statistics.’*** See 247, page 8.

12. Methods: Line 241-242: “Participants reporting and not reporting AEs were compared by baseline characteristics.” Please provide more details of this analysis.

**Response:** The sentence has been revised to read as follows: ***‘The baseline characteristics sex, age, previous COVID-19 status and presence of comorbidities were compared between participants reporting AEs and those not reporting AEs using descriptive statistics.’*** See line 249 - 251, page 8.

13. Methods: Line 257-258: “COVID-related deaths were excluded in this report and published in a separate effectiveness report.” Please provide a reference to the publication if these data have been published.

**Response:** A reference to the manuscript by Bekker, et al. published in the *Lancet* on 19 March 2022 has been included (<https://www.thelancet.com/journals/lancet/article/PIIS0140-6736(22)00007-1/fulltext>).

14. Results: Line 266-268: We suggest rephrasing to: “...(all 1 250 000 HCWs in the country were invited to participate).” or similar.

**Response:** This sentence has been rephrased as suggested.

15. Results: Line 279: Please change “less” to “fewer” AEs.

**Response:** This change has been made.

16. Results: Line 292-296: “Follow up at day seven post vaccination indicated that 92% of participants reporting AEs had either completely recovered or were recovering. The remaining 8% of participants were contacted by the safety team. Attempts were made to contact all these participants individually by the safety team (three telephonic calls at least one day apart). Those that were contactable were captured and if indicated, referred for further care.” Please include numbers in addition to reporting percentages here. Please clarify what is meant by “Those that were contactable were captured…” and please re-phrase this if possible.

**Response:** The statement has been revised to read: ***‘Attempts were made to contact all these participants individually by the safety team (three telephonic calls at least one day apart). Those that were contactable were interviewed via phone and if clinically indicated, referred for further care.’*** See line 307 - 309, page 9.

Further, we have revised the section for additional clarity and also now include numbers. It reads as follows: ***‘Follow up at day seven post vaccination indicated that of the 3,831 that had responded by dataset closure, 92% (3,524) of participants reporting AEs had either completely recovered or were recovering. The remaining 8% (324) of participants were contacted by the safety team.’*** See line 304 - 307, page 9-10.

17. Results: Line 296-297: “One in five (19%) AEs were not consistent classified as reactogenicity events” Please provide the actual number of AEs not classified as reactogenicity events.

**Response:** This sentence now includes the numbers.

18. Results: Line 307-308: “SAE outcomes were: 48 (34.8%) recovered, 36 (26.1%) recovering, 45 (32.6%) ongoing and 9 (6.5%) deceased.” Please mention the duration of follow up here, in light of the 32% of reports classified as ongoing and the following sentence mentioning events were followed up until resolution.

**Response:** This has now been clarified further by revising the sentences to reflect the duration of follow-up and provide additional clarity: ***‘SAE outcomes were: 48 (34.8%) recovered, 36 (26.1%) recovering and 9 (6.5%) deceased. All SAEs are followed until resolution. However, at the time of dataset closure on 15 June 2021 (28 days after the last vaccination), 45 SAEs (32.6%) were still ongoing.’*** See line 319 - 322, page 10.

19. Discussion: Line 392-394: We suggest phrasing this as: “A previous study of this vaccine supported its effectiveness against severe COVID-19 disease and COVID-19-related death after vaccination, and against both beta and delta variants [20].

**Response:** This sentence has been revised as suggested. Thank you.

20. Discussion: Line 397-398: We suggest revising to: “Furthermore, most SAEs were rare and occurred below expected rates. However, we did observe very rare events of TTS and GBS in this study at apparently higher than expected rates, though confidence intervals for these estimates were wide.” or similar.

**Response:** The revision has been made as suggested. Thank you.

21. Discussion: Line 405: We suggest “was lower with increasing age” instead of “reduced” here.

**Response:** The change has been made as suggested.

22. References: Please check the formatting of each reference, and please use the "Vancouver" style for reference formatting, and see our website for other reference guidelines <https://journals.plos.org/plosmedicine/s/submission-guidelines#loc-references>

**Response:** References have been reformatted.

23. When providing a DOI (e.g. for Reference 3), please give both the label and full DOI at the end of the reference. Do not provide a shortened DOI or the URL.
Please remove “[Internet]” throughout.
Please update the citation information for any preprints (e.g. reference 16).

**Response:** References have been revised and citations to published pre-prints updated.

24. Table 1: Please present p values for all comparisons. Please define all abbreviations used (AEs, IQR, HIV) in the legend.

**Response:** P-values are now presented in Table 1. The abbreviations are now also defined in the legend.

25. Table 2: Please define abbreviations used (PY, DRESS, GI) in the legend.

**Response:** The abbreviations have been defined.

26. Table 3: Please define abbreviations used (PY) in the legend. In the legend, please clarify if this should be “The rates are for adults (males and females combined) and are not stratified by age-group.”

**Response:** The suggested sentence reads better, and we have revised the current statement. The abbreviation for PY has also been added.

27. Figure 1: Please provide a descriptive legend for the graph.

**Response:** The descriptive legend is now provided.

28. Figure 2: Please describe “UL” and “LL” as well as indicating 2018 Medical Research Council Rapid Mortality Surveillance Report (RMS2018) and pre-COVID-19 local employee group life assurance data (Group assured) in the legend.

**Response:** This has been included.

29. CONSORT Checklist: Thank you for including the checklist. Please revise the checklist to refer to locations within the text with section and paragraph numbers (e.g. Methods, paragraph 1). Please do not refer to page numbers.

**Response:** The checklist has been revised.

30. Supplementary Table 1: Please spell out the abbreviation “BP” in the legend.

**Response:** Blood pressure (BP) has now been written in full.

31. Supplementary Table 2: Please define abbreviations (PY, CI, O/E) in the legend.

**Response:** Abbreviations for O/E, PY and CI have been included in the legend.

32. Supplementary Table 3: Please define abbreviations (IQR, HIV) in the legend.

**Response:** The abbreviations are fully defined now.

33. Supplementary Appendix 2: Please note that pages 2-6 contain contact information (addresses/phone numbers/email addresses), please remove this information if it should not be made publicly available.

**Response:** This information has been redacted. Thank you.


Comments from Reviewers:

Reviewer #1: The authors have addressed my points but there remains one minor issue outstanding. I asked for Supplementary Table 1 to give us more details about these other adverse events broken down by the effect on the participant (unable to perform usual activities, hospitalised). I perhaps was not clear enough as the authors' response just outlines what response the clinical and research team made to the events. This is valuable to know but not what I was asking for.

Michael Dewey

**Response:** Response: We understand the importance of Reviewer 1’s question. Unfortunately, we do not have adequate data to provide detailed severity grades for each and every adverse event reported. We are however able to provide aggregate severity grades for all AEs reported (see line 300 to 304, page 9) and specific severity grades for SAEs and AESIs. The database and collection was designed to interrogate further those AEs classified as serious or of special interest.

Reviewer #2: Authors correctly addressed the comments of reviewers

**Response:** Thank you for your review.
