# Supplementary material for: Safety evaluation of the single-dose Ad26.COV2.S vaccine among healthcare workers in the Sisonke study in South Africa: A phase 3b implementation trial
Source: PLoS Med. 2022 Jun 21;19(6):e1004024. doi: 10.1371/journal.pmed.1004024 (PMC9212139; doi:10.1371/journal.pmed.1004024)
Supplement: Attachment — Submitted filename: Responses to reviewers.RV3.docx [file pmed.1004024.s014.docx]

Caitlin Moyer, Ph.D.	17 May 2022

Associate Editor

PLOS Medicine

Dear Dr Caitlin Moyer,

**Re: Manuscript Number PMEDICINE-D-21-04884R2 – Response to editorial and remaining reviewer’s comments**

We are grateful to you and the Reviewers for providing additional comments on our manuscript, entitled *‘Safety evaluation of the single-dose Ad26.COV2.S vaccine among healthcare workers in the Sisonke study in South Africa: A phase 3b implementation trial’.*

Please find below our responses to the comments. We have uploaded a marked-up copy and a clean version of the revised manuscript. Line numbers refer to the tracked version.

**Requests from the Editor**

1. Line 69-70: Please move the clinical trial information to the end of the abstract. “ClinicalTrials.gov number NCT04838795; Pan-African Clinical Trials registry number PACTR202102855526180.”

**Response:** The trial registration details have been moved to the end of the abstract. Line 97-98.

2. Abstract: Line 86-87: Please revise to “Most serious AEs and AEs of special interest (n=138) occurred at lower than the expected population rates.”

**Response:** The revision has been made.

3. Line 90: Please spell out “SAE” at first use in the abstract.

**Response:** As SAE is also mentioned earlier as an abbreviation, we have spelt out the earlier mention on line 85.

4. Author summary: Line 116: Please re-phrase or clarify what is meant by “the large roll-out products.”

**Response:** This was a typo. Thank you. The sentence is revised to read “This supports continued use of this vaccine in the large roll-out programs.”

5. Line 299-300: Please remove the extraneous text from this paragraph. “One in five (19%, 2,109) AEs were not consistently classified as reactogenicity events” Please provide the actual number of AEs not classified as reactogenicity events.”

**Response:** The following revision has been made:” A total of 2,109 AEs were not classified as reactogenicity events.”

6. Table 3, Supplementary Table 2: In the table legends, please define the numbers in parentheses in the expected incidence rate columns.

**Response:** This has been revised in both tables. Included in the legend is that this is a “Reference to literature from which the background incidence was derived.” The references have also been updated.

7. Reference list: Please remove reference 17 or replace with another reference that is accessible. The reference list may not include unavailable and unpublished work. If relevant you may include the information or data as supplementary material or deposit the data in a publicly available database.

**Response:** The medical insurance data has now been included in aggregate form as Supplementary Appendix 4 and is referenced to in line 263 in the Methods section.

8. Table 3, Supplementary Table 2: In the table legends, please define the numbers in parentheses in the expected incidence rate columns.

**Response:** This has been revised in both tables. Included in the legend is that this is a “Reference to literature from which the background incidence was derived.” The corresponding references have also been updated.

9. Page 17: Please also list the CONSORT Checklist, Protocol, CRF, and SAP as supporting information files.

**Response:** These have been included.
